# Highly chemoselective synthesis of hindered amides via cobalt-catalyzed intermolecular oxidative hydroamidation

**DOI:** 10.1038/s41467-021-22373-z

**Published:** 2021-05-05

**Authors:** Yun-Nian Yin, Rui-Qi Ding, Dong-Chen Ouyang, Qing Zhang, Rong Zhu

**Affiliations:** Beijing National Laboratory for Molecular Sciences, Key Laboratory of Bioorganic Chemistry and Molecular Engineering of Ministry of Education, College of Chemistry and Molecular Engineering, Peking University, Beijing, China

**Keywords:** Homogeneous catalysis, Synthetic chemistry methodology

## Abstract

α-Tertiary amides are of great importance for medicinal chemistry. However, they are often challenging to access through conventional methods due to reactivity and chemoselectivity issues. Here, we report a single-step approach towards such amides via cobalt-catalyzed intermolecular oxidative hydroamidation of unactivated alkenes, using nitriles of either solvent- or reagent-quantities. This protocol is selective for terminal alkenes over groups that rapidly react under known carbocation amidation conditions such as tertiary alcohols, electron-rich alkenes, ketals, weak C−H bonds, and carboxylic acids. Straightforward access to a diverse array of hindered amides is demonstrated, including a rapid synthesis of an aminoadamantane-derived pharmaceutical intermediate.

## Abstract (web summary)

α-Tertiary amides are common in bioactive natural products and pharmaceuticals, but challenging to access by conventional methods. Here, the authors report a single-step approach toward α-tertiary amides via cobalt-catalyzed intermolecular oxidative hydroamidation of unactivated alkenes.

## Introduction

**Fig. 1:**
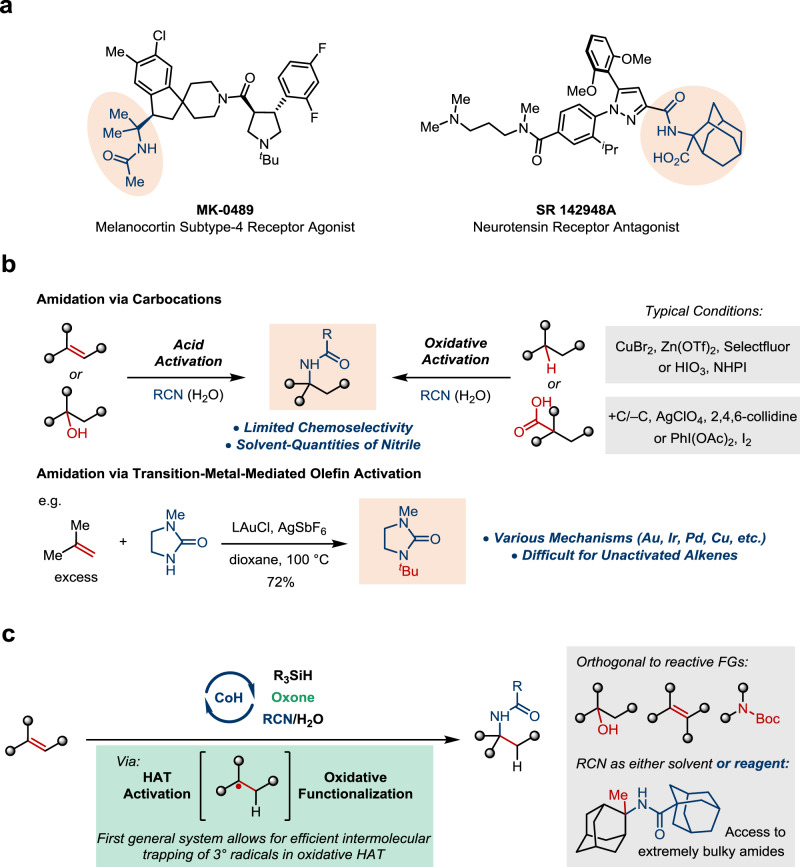
Importance of α-tertiary amides and synthetic strategies. **a** Representative pharmaceutically relevant α-tertiary amides. **b** Previous work of α-tertiary amides synthesis. **c** Chemoselective Co-catalyzed oxidative hydroamidation.

α-Tertiary (*N*-tertiary alkyl-substituted) amines and amides are widely found in biologically active natural products and pharmaceuticals (Fig. 1a)^1–4^. The extensive substitution of the C–N bond makes such motifs highly valuable for medicinal chemistry because of the improved lipophilicity and metabolic stability^5^. However, for the same reason, they are challenging targets synthetically. The most straightforward approach involves the addition of a carbon nucleophile to a ketimine, which often lacks adequate reactivity^6^. Alternatively, intramolecular transformations^7^ and radical amination^8–15^ have been explored. It is noteworthy that many α-tertiary amine derivatives obtained by the aforementioned methods are converted to the corresponding amides by multistep downstream processes while constructing such hindered amide bonds in an intermolecular setting is equally challenging^16^. In this context, single-step approaches leading to α-tertiary amides are highly desired.

A potential solution involves intercepting a tertiary carbocation with a nitrile, known as the Ritter-type amidation (Fig. 1b). Traditionally, it requires strong Brønsted or Lewis acids for substrate activation^17,18^. Recent advances highlight oxidative activation as an alternative. For example, Baran and coworkers demonstrated Ritter-type amidation initiated by C–H abstraction or electrochemical oxidative decarboxylation^19,20^. Similar processes mediated by I(III) have been reported^21–24^. Related trapping processes can be found in a number of alkene radical difunctionalization reactions, but mostly limited to styrenes^25–28^. Nonetheless, the chemoselectivity issue remains largely unsolved and could be further complicated by the presence of multiple weak C–H bonds or oxidizable sites. Besides, the high-energy nature of carbocation species necessitates using solvent quantities of the nitrile nucleophile, which significantly limits the synthetic application. As a second and conceptually distinct approach, intermolecular hydroamidation via transition metal-mediated π-bond activation has been pursued. However, for years limited success has been achieved with the Markovnikov-selective functionalization of unactivated olefins, and examples of α-tertiary amide synthesis are particularly rare^29–33^.

Inspired by these achievements, we proposed a strategy that merges the key concepts from both approaches discussed above, namely carbocation chemistry and metal-mediated olefin activation. More specifically, we hypothesized an intermolecular hydroamidation process of non-activated alkenes leveraging a hydrogen-atom-transfer (HAT)/oxidative trapping sequence (Fig. 1c)^34–36^. Along with the work from Mukaiyama^37^, Carreira^38^, Norton^39^, Shenvi^40^, Herzon^41^, Shigehisa^42–46^, and many others^47–56^, we expected that exceptional chemoselectivity could be realized by using HAT for activation. Such a process specifically targets alkenes and should be orthogonal to functional groups that are reactive to acid or oxidation. Moreover, alkenes could be differentiated by their steric hindrance in addition to electronic natures, which enables even more precise control. The key challenge lies in the second stage. Intermolecular oxidative trapping of a tertiary alkyl radical in Co-catalyzed hydrofunctionalization reactions has been essentially unsuccessful so far, as a result of facile alkene isomerization or hydration^36,45^.

Here, we report the realization of this design by a system featuring a combination of a CoSalen catalyst and oxone, a green oxidant. This modification successfully overcomes the tertiary radical trapping problem in previously reported systems promoted by either I(III) or *N*-fluorocollidiniums^36,45^. Meanwhile, it leads to a significantly more eco- and economically friendly process. Importantly, we show that nitriles can be employed in reagent quantities. Syntheses of a range of α-tertiary amides are demonstrated with high chemoselectivity, including hindered structures that are difficult to access otherwise.

## Results

### Investigation of reaction conditions

**Fig. 2:**
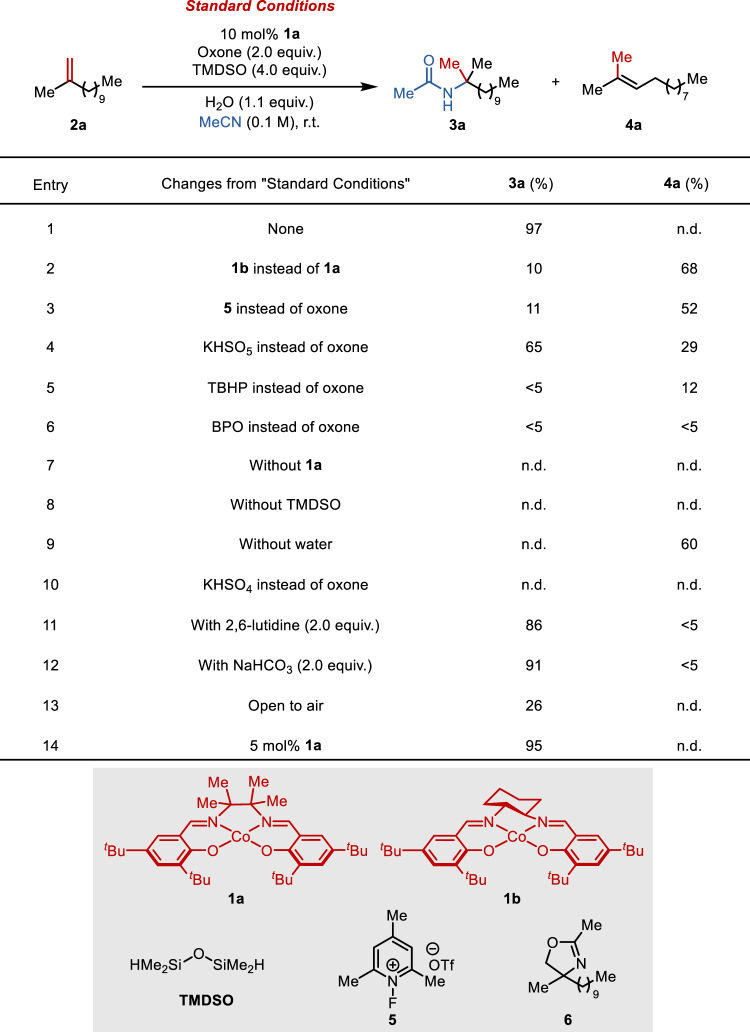
Evaluation of reaction conditions. Standard conditions: **2a** (0.10 mmol), **1a** (10 mol%), oxone (2.0 equiv.), TMDSO (4.0 equiv.), H_2_O (1.1 equiv.) in 1.0 mL MeCN at r.t. for 18 h. Yields were determined by ^1^H NMR analysis of the crude reaction mixture. TBHP   *tert*-butyl hydroperoxide, BPO   benzoyl peroxide.

We commenced our investigation by studying the oxidative hydroamidation reaction of 2-methyldodec-1-ene (**2a**) in acetonitrile as the solvent (Fig. 2). The combination of a catalytic quantity of CoSalen complex **1a**, tetramethyldisiloxane (TMDSO) as a hydride source, and oxone as a stoichiometric oxidant successfully promoted the desired transformation, affording the desired α-tertiary acetamide **3a** in over 95% yield (entry 1). In contrast, CoSalen **1b** bearing a less sterically bulky backbone furnished mostly isomerized alkene **4a** (entry 2). As mentioned earlier, isomerization dominated when oxone was replaced by *N*-fluorocollidinium salt **5**, a specialized oxidant previously found particularly effective for several intramolecular transformations by Shigehisa et al. (entry 3)^46^. Possible reasons include: (1) rapid unproductive decomposition of **5** in polar solvents catalyzed by **1a** could gradually divert the reaction to the HAT-type isomerization pathway due to a lack of oxidant supply, while the heterogeneity of oxone may alleviate this problem; (2) the reduction product derived from **5**, namely a collidine, is quite basic and may facilitate isomerization by promoting the deprotonation of a carbocation or its equivalent, while oxone would be reduced to much less basic bisulfates. Indeed, adding a super excess of 2,6-lutidine (5.0 or 10 equiv.) to the standard reaction led to an increase in isomerization (Supplementary Fig. 3). Interestingly, KHSO_5_, the active ingredient of oxone, was found much less effective by itself (entry 4). A few peroxy-containing oxidants were next tested and all found inferior (entries 5–6).

Control experiments established the essential roles of the catalysts, silane, and water, respectively (entries 7–9). In the absence of the silane, cobalt-mediated epoxidation took place and eventually furnished oxazoline **6** (~10% yield). This suggests that the hydride transfer from the silane to the oxidized cobalt species is likely rapid so that competing oxygen atom transfer can be avoided. Anhydrous conditions drove the reaction toward isomerization (the amount of degassed water added was studied (Supplementary Fig. 4). Essentially, no hydration product was observed at H_2_O <20 eq. With 100 μL water added, hydration (12%) was observed with the desired amidation completely inhibited). Neither amidation nor isomerization was observed when oxone was substituted by KHSO_4_ (entry 10). In addition, the hydroamidation process was not affected by the presence of an excess base (entries 11–12). Therefore, Brønsted acid-mediated amidation is unlikely involved in this system. Performing the reaction under ambient atmosphere resulted in substantially decreased yield due to oxygen trapping (entry 13). Finally, the catalyst loading could be lower to 5 mol% without compromising the yield (entry 14).

### Substrate scope

**Fig. 3:**
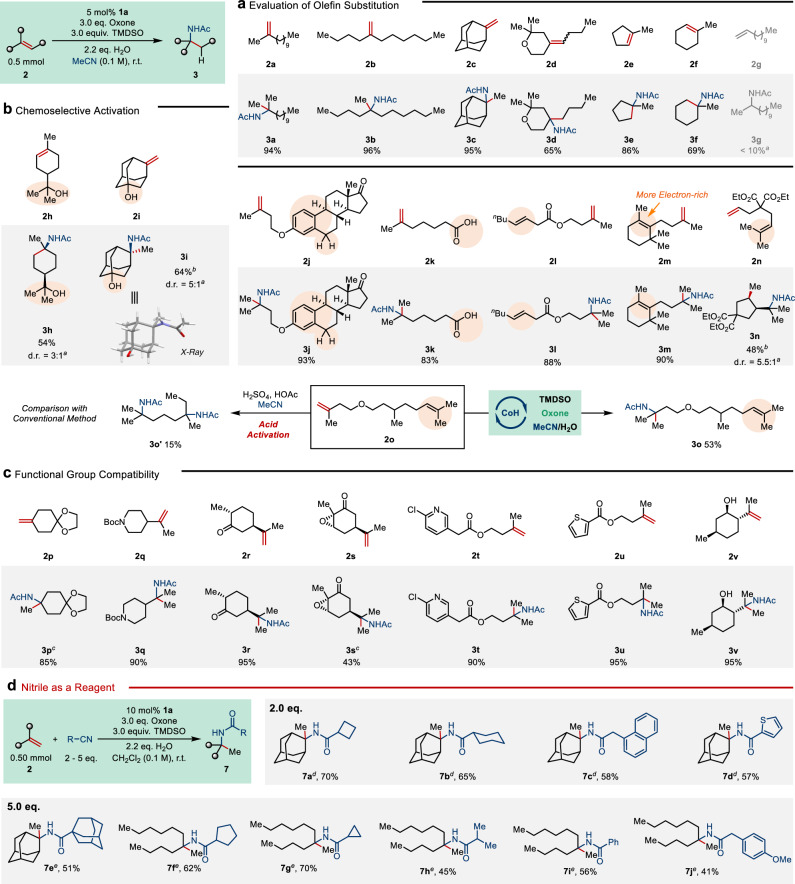
Scope of the Co-catalyzed hydroamidation. **a** Evaluation of olefin substitution. **b** Demonstration of chemoselective activation. **c** Evaluation of Functional group compatibility. **d** Using reagent quantities of nitrile. Unless noted otherwise, yields correspond to isolated, analytically pure material. Conditions: **2** (0.50 mmol), **1a** (5 mol%), oxone (3.0 equiv.), TMDSO (3.0 equiv.), H_2_O (2.2 equiv.) in 5.0 mL MeCN at r.t. for 18 h. ^*a*^Determined by ^1^H NMR analysis of the crude reaction mixture. ^*b*^Isolated yield of the major diastereomer. ^*c*^With additional 2,6-lutidine (2.0 equiv). ^*d*^**2** (0.50 mmol), RCN (1 mmol), **1a** (10 mol%), oxone (3.0 equiv.), TMDSO (3.0 equiv.), H_2_O (2.2 equiv.) in 5.0 mL CH_2_Cl_2_ at r.t. for 18 h. ^*e*^**2** (0.50 mmol), RCN (2.5 mmol), **1a** (10 mol%), oxone (3.0 equiv.), TMDSO (3.0 equiv.), H_2_O (2.2 equiv.) in 5.0 mL CH_2_Cl_2_ at r.t. for 18 h.

With the optimized protocol in hand, we sought to evaluate the scope and limitation of this method (Fig. 3). First, the effect of alkene substitution was examined (Fig. 3a). 1,1-dialkyl substituted olefins gave nearly quantitative yields (**2a**–**c**). Acyclic trisubstituted alkenes such as **4a** were found unreactive, but their exo- and endo-cyclic counterparts are viable substrates (**2d**–**f**). While focusing on α-tertiary amides synthesis, we tested monoalkyl-substituted alkenes and found them substantially less reactive (**2g**) (isomerization (3%) and hydrogenation (55%) were found as major products for the reaction of **2g** (conversion 64%). This could be attributable to the slower oxidation of a secondary alkyl radical via the corresponding organocobalt(III) intermediate.).

Next, highly chemoselective hydroamidation of terminal olefins was demonstrated, which highlights its selectivity for terminal alkenes over groups that undergo facile Ritter-type amidation upon either acid or oxidative activation (Fig. 3b). Tertiary alcohols were well-tolerated including an admantane-1-ol (**2h, i**). Weak benzylic C–H bonds associated with an electron-rich arene and carboxylic acids also remained intact during the reaction (**2j, k**). Notably, it has been reported that **2j** underwent Friedel–Crafts-type cyclization using *N*-fluorocollidinium **5** as an oxidant for cobalt^44^. The absence of such cyclization here clearly indicates the mechanistic distinction of our CoSalen/oxone system that turns on the intermolecular trapping pathway.

The advantage of HAT activation is best illustrated by the complete selectivity for terminal alkenes (**2l**–**2o**). Tolerating a tetrasubstituted olefin (**2m**) is particularly non-trivial as it is substantially more reactive toward electrophiles or oxidation. The proposed HAT mechanism was further supported by the formation of a radical cyclization product (**3n**) (an additional radical clock experiment is shown in SI-34. The absence of uncyclized trapping products suggests that the radical–polar crossover process under these conditions is probably slower than a typical 5-*exo*-trig radical cyclization.). A head-to-head comparison was then made between the protocol and the acid-mediated process. While subjecting diene **2o** to conventional conditions only produced a complex mixture containing degradation products derived from ether bond cleavage, the Co-catalyzed method smoothly afforded selective mono-hydroamidation on the more accessible alkene moiety.

We continued looking into the chemoselectivity with particular emphasis on acid-sensitive protecting groups, for instance, ketal and *N*-Boc groups (Fig. 3c, **3p**, **q**). It is noted that the HSO_4_^−^ (p*K*_a_ = 2.0) that was generated as a byproduct is relatively acidic so an additional base (2,6-lutidine) was necessary to neutralize the excess protons in the reaction of **3p**, while the *N*-Boc group in **3q** was compatible without any buffering. Additional examples were tested containing ketones, epoxides, six- and five-membered heterocycles (**3r**–**u**). Excellent compatibility was observed in all cases. In the presence of a homoallylic hydroxy group, dihydrooxazine was initially formed via intramolecular nitrilium trapping, which opened up to give the amide product in high yield upon basic aqueous workup (**3v**).

Whereas the result shown above are encouraging, we were aware of the severe limitation of using solvent quantities of nitriles, which the vast majority of the examples regarding Ritter-type amidation relies on. It has proven quite difficult to use nitriles as a reagent, even in large excess^27^. Nonetheless, a few groups including our own have recently collected evidence supporting the intermediacy of organocobalt(IV) species in oxidative hydrofunctionalization reactions^36,43,53^. Thus, we wondered if such species, though likely being short-lived due to the weak 3^°^ alkyl−Co bond, could still affect some control on the generation of the carbocations (alkyl migration to the ligand in a [RCo(IV)Salen]^+^ complex could serve as an alternative mechanism for such control, see ref. ^57^). Such a process might enable the desired transformation at a lower concentration of the nucleophile.

To our delight, switching to dichloromethane as the solvent, a modified protocol was found amenable for hydroamination using reagent quantities of the nitriles. Although excess nitrile (2–5 equiv.) was still necessary for obtaining decent yields, this has allowed us to explore a variety of nitriles for α-tertiary amide synthesis (Fig. 3d). Alkyl, aryl, and heteroaryl nitriles were all found suitable for hydroamidation. Either an increase in the steric bulk of the nitrile or a decrease in the catalyst loading led to diminished yields. For reactions with acyclic alkene substrates (**7f**–**j**), the remaining mass balance was primarily isomerized alkene. As a case of severe steric congestion, a formidable amide linking two adamantyl groups was synthesized in over 50% yield (**7e**).

### Gram-scale experiment

**Fig. 4:**
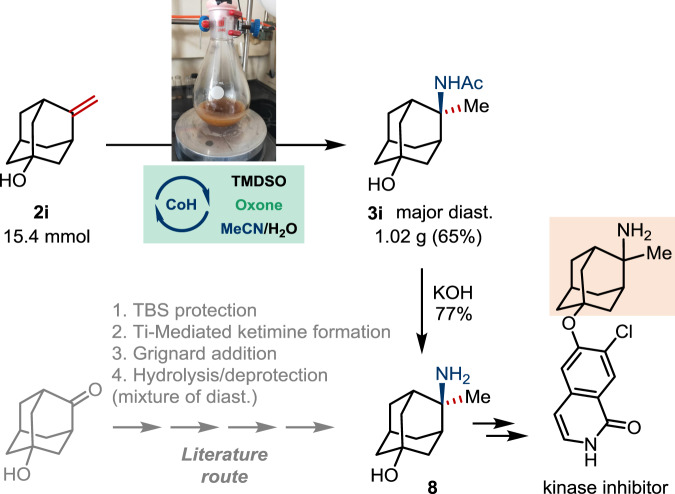
Synthetic application. Gram-scale hydroamidation of **2i**.

To further demonstrate the synthetic utility of this protocol, we targeted aminoadamantane derivative **8**, an intermediate en route to a Rho kinase inhibitor (Fig. 4)^58^. The adamantane structure has been extensively employed as a lipophilic “add-on” for pharmacophore modification, which improves the pharmacokinetics of a drug. Monofunctional adamantane building blocks are readily available through carbocation chemistry. In contrast, the synthesis of equally important bifunctional analogs like **8** often involves protecting group manipulations or hazardous materials (e.g., azides)^5^.

To this, we show that the gram-scale hydroamidation of **2i** proceeded smoothly under the standard conditions. The major diastereomer of the resulting amide **3i** was isolated in decent yield, which was converted to the free amine **8** upon basic hydrolysis. This provides a more rapid alternative to the literature route, where a mixture of diastereomers was delivered through a multistep sequence involving silyl protecting groups, cryogenic operations, and stoichiometric Ti(IV) salts.

### Mechanistic studies

**Fig. 5:**
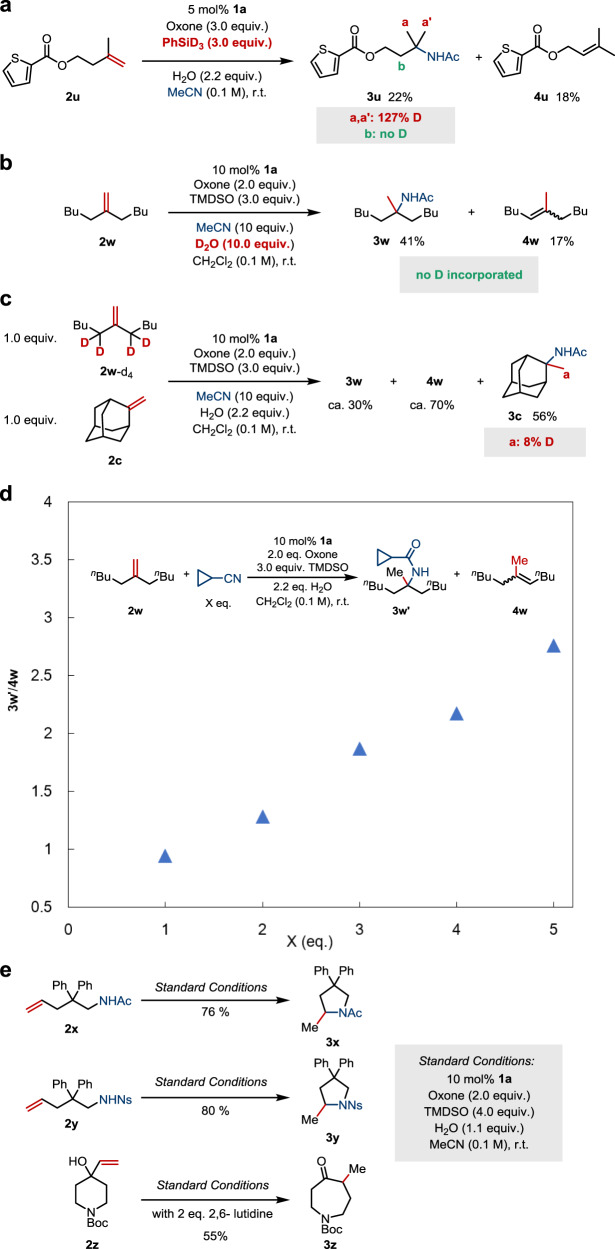
Mechanistic investigation. **a** With deuterium-labeled silane. **b** Test for proton activation. **c** Test for reversibility of HAT. **d** Effect of the amount of nitrile. **e** Tests of alternative nucleophiles.

A set of deuterium-labeling experiments were performed to shed light on the mechanism of this reaction (Fig. 5). First, PhSiD_3_ was employed in place of TMDSO in the hydroamidation reaction of **2u** (Fig. 5a). At the outset, 127% D was incorporated at C(a, a′) of **3u**, confirming that the silane serves as the hydride source for HAT. Deuterium incorporation of higher than 100% supports equilibrium intermediacy of an alkene after HAT that causes H/D scrambling. Similar observations were made in related Mn-catalyzed hydrogenation reactions reported by Shenvi^59^. No D was found at C(b), indicating neglectable HAT of trisubstituted alkene **4u**. The reaction in the presence of a large excess of D_2_O produced corresponding acetamide **3w** and isomerized olefin **4w** without detectable deuteration at any carbons (Fig. 5b). This suggests the absence of proton-mediated alkene activation in the formation of either product, which corroborates the control experiment results shown in Fig. 2 and excellent chemoselectivity observed.

Next, we carried out a crossover reaction using a mixture of **2w**-d_4_ and **2c** (Fig. 5c). A substantial amount of the alkene isomer **4w** was formed under these conditions, and deuterium crossover was detected in the amide product **3c** (8% D at C(a)). This indicates the involvement of Co−D species, which is likely derived from the HAT-type isomerization of **2w**-d_4_.

It is noted that carbocation deprotonation might be an alternative or co-existing pathway for isomerization, which has been proposed to account for the different reaction outcomes observed in Co-catalyzed alkene isomerizations under redox-neutral and -oxidative conditions^60^. To probe this possibility, we performed the reactions with varying amounts of nitrile (Fig. 5d). Full conversion of **2w** was reached, and a good mass balance was achieved in each reaction. A strong positive correlation was observed between the concentration of the nucleophile and the yield of the desired transformation (**3w’**) over isomerization (**4w**). This implies that while HAT-type isomerization exists, **3w’** and a significant portion of **4w** could be derived from a common intermediate, either a carbocation or its equivalent.

Finally, the scope of the CoSalen-oxone system was expanded to encompass several intramolecular cyclizations involving nitrogen- and oxygen-based nucleophiles (Fig. 5e). Decent yields of *N*-protected pyrolidine (**3x**,**y**) and an azacycloheptanol derivative (**3z**) were obtained without optimization, which demonstrates the potential of this system as green and general alternative to existing methods^55,61^.

**Fig. 6:**
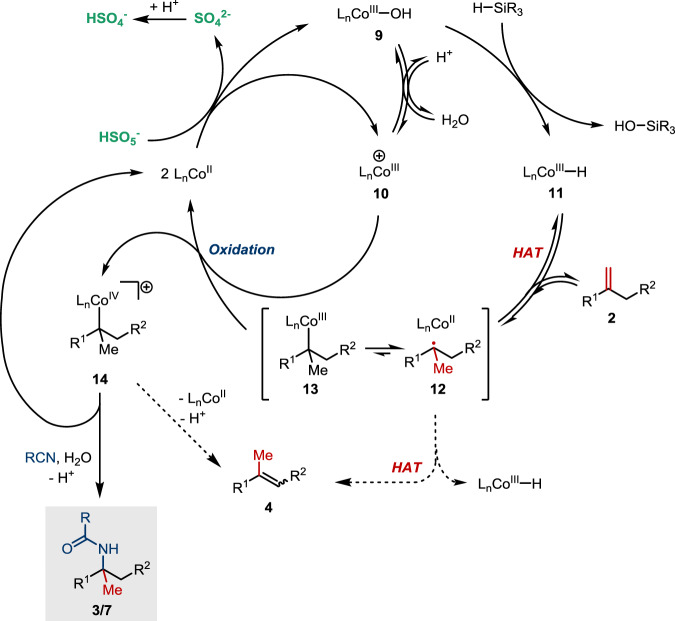
Proposed catalytic cycle. Organocobalt(IV) species as a carbocation equivalent leads to amidation.

A plausible catalytic cycle is depicted in Fig. 6. The persulfate could serve as a two-electron oxidant to deliver a Co(III)–OH species **9** and a cationic Co(III) complex **10**. Such group/atom transfer process is consistent with the mass spectroscopic data of a mixture of **1a** and oxone in acetonitrile (Supplementary Fig. 5), as well as literature reactions between peroxides and relevant Co(II) complexes^62^. It has been proposed that Co(III) species bearing a silicophilic ligand such as an alkoxide or fluoride could react with a silane to give a Co−H species **11**, although the mechanistic pathways of the hydride transfer are not clear^50^. Therefore, it is expected that **9** could behave similarly to give a Co−H species **11**, which then undergoes HAT with an alkene **2** in a reversible fashion. The resulting tertiary radical **12** might be trapped to form an organocobalt species **13** containing a very weak Co−C bond. Oxidation of **13** by the cationic species **10** would affect radical–polar crossover to generate the corresponding organocobalt(IV) species **14**, which might serve as a carbocation reservoir or equivalent that is subsequently trapped by a nitrile in the presence of water to complete the amidation. The alkene isomerization could take place from either radical **12** or organocobalt(IV) **14**, via HAT or deprotonation, respectively.

## Discussion

In conclusion, we have developed a general and scalable Co-catalyzed hydroamidation protocol using unactivated alkenes and nitriles of either solvent- or reagent quantities. An optimal system composed of CoSalen/oxone/silane was found crucial to enable efficient HAT followed by oxidative functionalization of the resulting 3^°^ alkyl radical intermediates, which delivers a wide range of α-tertiary amides with excellent chemoselectivity. This method provides an alternative approach toward challenging amides that are difficult to access due to steric hindrance or sensitive functional groups.

## Methods

### General procedures for cobalt-catalyzed intermolecular oxidative hydroamidation

An oven-dried 25-mL re-sealable Schlenk tube equipped with a Teflon-coated magnetic stir bar was charged with Co catalyst **1a** (15 mg, 0.025 mmol, 0.05 equiv.) and oxone (100 mesh, 922.2 mg, 1.5 mmol, 3.0 equiv.). The reaction vessel was then briefly evacuated and backfilled with nitrogen (this sequence was repeated a total of three times). Anhydrous acetonitrile (5.0 mL), **2** (0.50 mmol, 1.0 equiv.), H_2_O (20 μL, 1.1 mmol, 2.2 equiv.) and 1,1,3,3-tetramethyldisiloxane (275 μL, 1.5 mmol, 3.0 equiv.) were added to the reaction vessel via syringe sequentially. The reaction mixture was stirred at r.t. for 18 h. The mixture was filtered through a short pad of silica gel with CH_2_Cl_2_/MeOH (20/1) as an eluent. The solvents were removed in vacuo and the residue was purified by silica gel column chromatography to afford amide product **3**.

An oven-dried 25 mL re-sealable Schlenk tube equipped with a Teflon-coated magnetic stir bar was charged with Co catalyst **1a** (30 mg, 0.05 mmol, 0.1 equiv.) and oxone (100 mesh, 922.2 mg, 1.5 mmol, 3.0 equiv.). The reaction vessel was then briefly evacuated and backfilled with nitrogen (this sequence was repeated a total of three times). Anhydrous dichloromethane (5.0 mL), nitrile (1.0 mmol or 2.5 mmol), **2** (0.50 mmol, 1.0 equiv.), H_2_O (20 μL, 1.1 mmol, 2.2 equiv.) and 1,1,3,3-tetramethyldisiloxane (275 μL, 1.5 mmol, 3.0 equiv.) were added to the reaction vessel via syringe sequentially. The reaction mixture was stirred at r.t. for 18 h. The mixture was filtered through a short pad of silica gel with CH_2_Cl_2_/MeOH (20/1) as an eluent. The solvents were removed in vacuo and the residue was purified by silica gel column chromatography to afford amide product **8**.

## Supplementary information

Supplementary Information

## Data Availability

The authors declare that the data supporting the findings of this study are available within the paper and its Supplementary Information file. The X-ray crystallographic coordinates for structures of **3****h** and **3i** have been deposited at the Cambridge Crystallographic Data Centre (CCDC) under deposition numbers CCDC 2026144 [10.5517/ccdc.csd.cc260ch3] and CCDC 2025806 [10.5517/ccdc.csd.cc2600lv]. The data can be obtained free of charge from the Cambridge Crystallographic Data Centre via http://www.ccdc.cam.ac.uk/data_request/cif. The experimental procedures and characterization of all new compounds are provided in Supplementary Information.
