# Supplementary material for: Highly chemoselective synthesis of hindered amides via cobalt-catalyzed intermolecular oxidative hydroamidation
Source: Nat Commun. 2021 May 5;12:2552. doi: 10.1038/s41467-021-22373-z (PMC8100129; doi:10.1038/s41467-021-22373-z)
Supplement: Supplementary file 1 — Supplementary Information [file 41467_2021_22373_MOESM1_ESM.pdf]

Supplementary Information

Highly Chemoselective Synthesis of Hindered Amides via Cobalt-Catalyzed Intermolecular Oxidative Hydroamidation

Yun-Nian Yin, Rui-Qi Ding, Dong-Chen Ouyang, Qing Zhang and Rong Zhu*

Beijing National Laboratory for Molecular Sciences (BNLMS), Key Laboratory of Bioorganic Chemistry and Molecular Engineering of Ministry of Education, College of Chemistry and Molecular Engineering, Peking University, Beijing 100871, China

*Corresponding author: rongzhu@pku.edu.cn

Table of Contents

I. General Information S3

II. Procedures and Characterizations S4

II-A. Synthesis and Characterizations of non-Commercial Compounds.......................... S4

II-B. General Procedures for Cobalt-Catalyzed Intermolecular Oxidative Hydroamidation.. S7

II-C. Characterizations of Compounds.. S9

III. Gram-scale Experiment.. S27

IV. Mechanistic Studies... S29

IV-A. With Deuterium-Labeled Silane (Figure 5a).. S29

IV-B. Test for Proton-Activation (Figure 5b).. S29

IV-C. Test for Reversibility of HAT (Figure 5c)... S30

IV-D. Effect of the Amount of Nitrile (Figure 5d).. S31

IV-E. Tests of Alternative Nucleophiles (Figure 5e)... S31

IV-F. Effect of the Amount of 2,6- lutidine... S33

IV-G. Effect of the H_2O.. S33

IV-H. HRMS Analysis of Possible Intermediates.. S34

IV-I. Radical Clock Experiment.. S34

V. X-Ray Crystallography Analysis.. S36

VI. Supplementary References... S39

VII. NMR Spectra S41

I. General Information

All reactions were carried out with dry solvents under anhydrous conditions, unless otherwise noted. Reagents were purchased at the highest commercial quality and used without further purification, unless otherwise stated. Anhydrous dichloromethane, 1,1,3,3-tetramethyldisiloxane (CAS # 3277-26-7), *N*-fluoro-2,4,6-trimethylpyridinium triflate (**5**) (CAS # 107264-00-6) and 2,6-lutidine (CAS # 108-48-5) were purchased from J&K. (*R*,*R*)-(−)-*N*,*N*'-bis(3,5-di-*tert*-butylsalicylidene)-1,2-cyclohexanediaminocobalt(II) (CAS # 176763-62-5) was purchased from Strem and Energy Chemical. 1-Methylcyclopentene (**2e**) (CAS # 693-89-0) was purchased from 3A Chemical. 1-Methyl-cyclohexen (**2f**) (CAS # 591-49-1) was purchased from Alfa. 1-Dodecene (**2g**) (CAS # 112-41-4) and (+)-dihydrocarvone (**2r**) (CAS # 5524-05-0) were purchased from Energy Chemical. Alpha-terpineol (**2h**) (CAS # 98-55-5) was purchased from Bidepharm. $CDCl_3$ was purchased from Innochem. Oxone (CAS # 37222-66-5) was purchased from J&K and ground into fine powder (100 mesh). Anhydrous acetonitrile was purchased from Fisher and purified by passing through Vigor solvent purification system and stored under N_2. "PE" refers to petroleum ether. All chemicals were weighed on the bench top, in the air. Yields refer to chromatographically and spectroscopically (^{1}H NMR) homogeneous materials, unless otherwise stated. Reactions were monitored by ^{1}H NMR spectroscopy and thin-layer chromatography (TLC) carried out on silica gel plates (HSGF254) supplied by Yantai Chemicals (China), using UV light as a visualizing agent and phosphomolybdic acid in ethanol or $KMnO_4$ solution and heat as developing agents. Flash silica gel chromatography was performed using silica gel (200-300 mesh) supplied by Tsingtao Haiyang. ^{1}H and ^{13}C NMR spectra were recorded on a Bruker 400 spectrometer and were calibrated using $CDCl_3$ as internal reference ($CDCl_3$: 7.26 ppm for ^{1}H NMR and 77.2 ppm for ^{13}C NMR). The following abbreviations were used to explain the multiplicities: s = singlet, d = doublet, t = triplet, q = quartet, p = pentet, m = multiplet, br = broad. IR spectra were recorded on a Thermo Scientific Nicolet iS5 FT-IR spectrometer (iD5 ATR). High-resolution mass data was recorded by a Solarix XR Fourier Transform Ion Cyclotron Resonance Mass Spectrometer.

II. Procedures and Characterizations

II-A. Synthesis and Characterizations of non-Commercial Compounds

O-3-Methylbut-3-en-1-yl-estrone (**2j**),[1] 6-methylhept-6-enoic acid (**2k**),[2] (1*S*,4*S*,6*S*)-1-methyl-4-(prop-1-en-2-yl)-7-oxabicyclo[4.1.0]heptan-2-one (**2s**),[3] 3-methylbut-3-en-1-yl thiophene-2-carboxylate (**2u**),[4] [*N*,*N*'-(1,1,2,2-tetramethylethylene)bis(3,5-di-*tert*-butylsalicylideneiminato)]cobalt(II) (**1a**),[5] $PhSiD_3$[6] were prepared according to literature procedures.

General Procedure for Substrate Preparation

To a 250 mL oven-dried round bottom flask equipped with a magnetic stir bar was added methyltriphenylphosphonium bromide (10 g, 28 mmol, 1.4 equiv.) and anhydrous THF (35 mL). Potassium *tert*-butoxide (3.4 g, 30 mmol, 1.5 equiv.) was added and the reaction mixture was stirred for 30 min at r.t., during which time the reaction mixture turned yellow. A solution of ketone (20 mmol, 1.0 equiv.) in anhydrous THF (20 mL) was added drop-wise to the reaction mixture. The reaction mixture was stirred for an additional 1.5 h at r.t. before quenched by the addition of 50 mL H_2O. The resulting mixture was extracted with ethyl acetate and the combined organic layers were dried over Na_2SO_4, filtered, concentrated *in vacuo* and purified by silica gel chromatography to get 1,1-disubstituted alkenes. Following this procedure, **2a**,[7] **2b**,[8] **2c**,[9] **2i**,[10] **2m**,[11] **2p**,[12] **2q**[13], **2w**[14] were prepared, and their spectroscopic data matches literature reports.

4-Butylidene-2,2-dimethyltetrahydro-2H-pyran (2d)

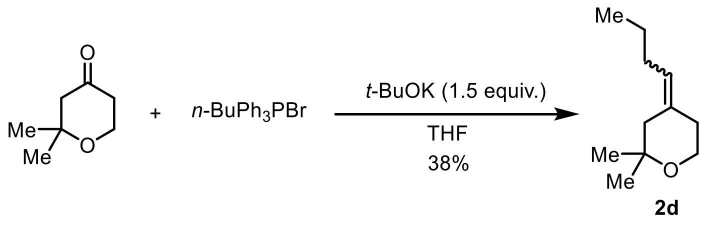

Following the general procedure for substrates preparation with slight modification, **2d** was synthesized from butyltriphenylphosphonium bromide (11.2 g, 28 mmol, 1.4 equiv.) and 2,2-dimethyltetrahydro-4H-pyran-4-one (2.6 g, 20 mmol, 1.0 equiv.). Purification by silica gel chromatography afforded **2d** (510 mg, 38% yield, *E*:*Z* = 1:1) as a colorless oil.

^{1}H NMR (400 MHz, $CDCl_3$) δ 5.28 (tt, J = 7.3, 1.3 Hz, 0.5H), 5.13 (tt, J = 7.4, 1.3 Hz, 0.5H), 3.68 (dt, J = 12.3, 5.7 Hz, 2H), 2.22 – 1.91 (m, 6H), 1.36 (hd, J = 7.3, 6.1 Hz, 2H),

1.18 (d, J = 6.0 Hz, 6H), 0.90 (t, J = 7.3 Hz, 3H).
^{13}C NMR (101 MHz, $CDCl_3$) δ 133.07, 132.92, 124.60, 124.45, 73.12, 72.90, 63.06, 62.02, 47.83, 40.46, 36.46, 29.19, 29.05, 29.00, 26.38, 26.21, 23.18, 23.14, 13.88, 13.73.
HRMS (ESI) $[M + H]^+$ calculated for $C_{11}H_{21}O$: 169.1587, found: 169.1587;
FTIR (cm^{-1}): 2958.7, 2927.9, 2862.4, 1363.5, 1257.4, 1186.8, 1083.3, 1048.6, 853.7, 744.4;
TLC: R_f = 0.95 (PE: ethyl acetate = 10:1).

3-Methylbut-3-en-1-yl (*E*)-oct-3-enoate (2l)

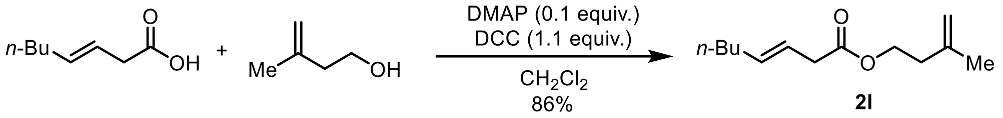

To a solution of 3-octenoic acid (1.4 g, 10.0 mmol, 1.0 equiv.) in CH_2Cl_2 (50 mL) at 25 °C was added 3-methylbut-3-en-1-ol (1.0 g, 11.0 mmol, 1.1 equiv.), followed by 4-dimethylaminopyridine (DMAP, 122.2 mg, 1.0 mmol, 0.10 equiv.) and *N*, *N*'-dicyclohexylcarbodiimide (DCC, 2.3 g, 11 mmol, 1.1 equiv.). The resulting mixture was stirred at r.t. overnight. The reaction mixture was filtered through celite and the filter cake was washed with ethyl acetate (3 × 10 mL). The resulting filtrate was concentrated and purified by flash column chromatography to afford **2l** (1.8 g, 86% yield) as a colorless oil.

^{1}H NMR (400 MHz, $CDCl_3$) δ 5.60 – 5.44 (m, 2H), 4.79 (s, 1H), 4.72 (s, 1H), 4.19 (t, J = 6.9 Hz, 2H), 3.01 (d, J = 5.7 Hz, 2H), 2.33 (t, J = 6.9 Hz, 2H), 2.02 (q, J = 6.4, 5.9 Hz, 2H), 1.75 (s, 3H), 1.32 (tdt, J = 8.5, 3.6, 2.2 Hz, 4H), 0.88 (t, J = 7.0 Hz, 3H).
^{13}C NMR (101 MHz, $CDCl_3$) δ 172.16, 141.61, 134.80, 121.49, 112.25, 62.73, 38.12, 36.67, 32.14, 31.29, 22.49, 22.16, 13.91.
HRMS (ESI) $[M + H]^+$ calculated for $C_{13}H_{23}O_2$: 211.1693, found: 211.1690;
FTIR (cm^{-1}): 2928.1, 2854.8, 2117.4, 1736.3, 1240.2, 1156.9, 967.8, 889.9, 842.1;
TLC: R_f = 0.85 (PE: ethyl acetate = 4:1).

2,6-Dimethyl-8-((3-methylbut-3-en-1-yl)oxy)oct-2-ene (2o)

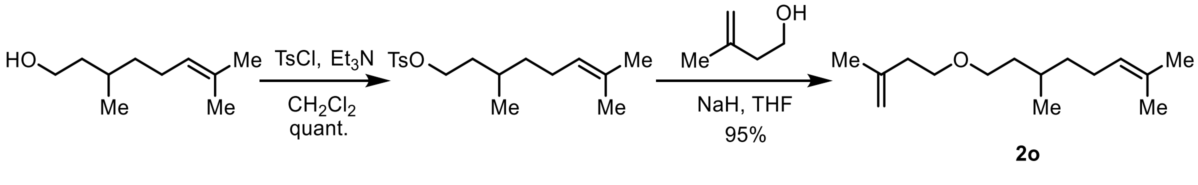

To a solution of citronellol (3.2 g, 20.0 mmol, 1.0 equiv.), Et_3N (3.4 mL, 24.0 mmol, 1.20 equiv.) in dry CH_2Cl_2 (40 mL) was added TsCl (4.2 g, 22.0 mmol, 1.1 equiv.) and the

resulting mixture stirred at r.t. overnight. The reaction mixture was quenched with saturated aqueous $NaHCO_3$ (25 mL) and extracted with dichloromethane (3 × 20 mL). The combined organic layers were dried over Na_2SO_4, filtered and concentrated *in vacuo* and purified via column chromatography (PE: ethyl acetate = 20:1) to yield 3,7-dimethyloct-6-en-1-yl 4-methylbenzenesulfonate as a colorless oil (quant.).[15]

In a 100-mL round-bottom flask equipped with a dropping funnel, a suspension of NaH (420 mg, 60% in mineral oil, 10.5 mmol) in THF (10 mL) was prepared under nitrogen atmosphere. A solution of 3-methyl-2-buten-1-ol (861 mg, 10 mmol) in THF (5 mL) was added dropwise under an ice bath, the mixture was stirred for 1 h, and 3,7-dimethyloct-6-en-1-yl 4-methylbenzenesulfonate was added. The resulting reaction mixture was stirred at r.t. overnight. The reaction mixture was quenched by H_2O, followed by addition of aqueous $NaHCO_3$ (20 mL) and extracted with Et_2O. The combined organic layers were dried over Na_2SO_4, filtered, concentrated *in vacuo* and purified by silica gel chromatography (PE: ethyl acetate = 15:1) to get **2o** (1.8 g, 95% yield) as a colorless oil.

^{1}H NMR (400 MHz, $CDCl_3$) δ 5.09 (tdd, J = 6.8, 2.8, 1.4 Hz, 1H), 4.76 (s, 1H), 4.72 (s, 1H), 3.54 – 3.41 (m, 4H), 2.34 – 2.26 (m, 2H), 1.96 (m, 2H), 1.74 (s, 3H), 1.67 (s, 3H), 1.65 – 1.50 (m, 5H), 1.44 – 1.28 (m, 2H), 1.20 – 1.10 (m, 1H), 0.89 (d, J = 6.5 Hz, 3H).
^{13}C NMR (101 MHz, $CDCl_3$) δ 141.99, 130.07, 123.83, 110.27, 68.35, 68.21, 36.83, 36.21, 35.65, 28.57, 24.70, 24.46, 21.75, 18.55, 16.60.
HRMS (ESI) $[M + H]^+$ calculated for $C_{15}H_{29}O$: 225.2213, found: 225.2213;
FTIR (cm^{-1}): 3411.2, 2986.4, 1716.6, 1458.2, 1377.2, 1275.3, 1259.0, 1105.3, 749.8;
TLC: R_f = 0.95 (PE: ethyl acetate = 10:1).

3-Methylbut-3-en-1-yl 2-(6-chloropyridin-3-yl)acetate (2t)

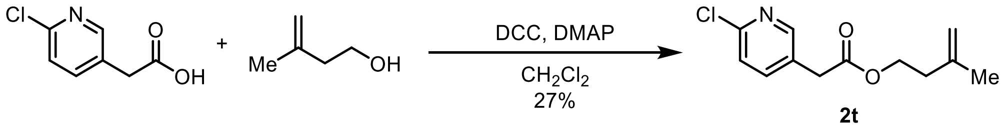

Following procedure for **2l**. **2t** was synthesized from 2-(6-chloropyridin-3-yl)acetic acid (2.6 g, 15.0 mmol). Purification by silica gel chromatography afforded **2t** (972 mg, 27% yield) as a colorless oil.

^{1}H NMR (400 MHz, $CDCl_3$) δ 8.28 (d, J = 2.5 Hz, 1H), 7.62 (dd, J = 8.2, 2.5 Hz, 1H), 7.30 (d, J = 8.2 Hz, 1H), 4.78 (s, 1H), 4.69 (s, 1H), 4.23 (t, J = 6.7 Hz, 2H), 3.61 (s, 2H), 2.33 (t, J = 6.8 Hz, 2H), 1.73 (s, 3H).
^{13}C NMR (101 MHz, $CDCl_3$) δ 170.18, 150.30, 150.13, 141.27, 139.75, 128.74, 124.04, 112.54, 63.35, 37.62, 36.60, 22.31.

HRMS (ESI) $[M + H]^+$ calculated for $C_{12}H_{15}ClNO_2$: 240.0786, found: 240.0783;
FTIR (cm^{-1}): 2968.4, 1731.5, 1650.9, 1587.9, 1566.5, 1459.2, 1384.8, 1335.2, 1139.4;
TLC: R_f = 0.90 (PE: ethyl acetate = 1:1).

2w-d_4 (6-Methyleneundecane-5,5,7,7-d_4)

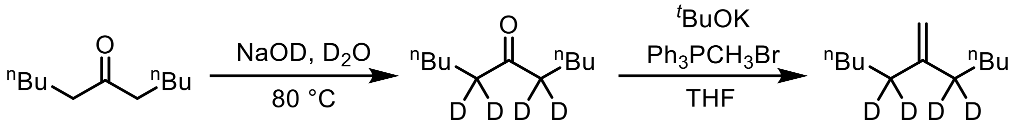

2w-d_4

To a 25 mL oven-dried round bottom flask containing a magnetic stir bar was added undecan-6-one (1 mL, 4.90 mmol), NaOD (40% w/w in D_2O, 1 mL). The reaction mixture was refluxed overnight. The resulting mixture was dried over Na_2SO_4, concentrated *in vacuo*. This sequence was repeated a total of three times to afford undecan-6-one-5,5,7,7-d_4 (267 mg, 31%) as the literature reported.[16]

To a 50 mL oven-dried round bottom flask equipped with a magnetic stir bar was added methyltriphenylphosphonium bromide (765 mg, 2.0 mmol, 1.4 equiv.) and anhydrous THF (15 mL). Potassium *tert*-butoxide (22.3 mg, 1.9 mmol, 1.3 equiv.) was added and the reaction mixture was stirred for 30 min at r.t., during which time the reaction mixture turned yellow. Undecan-6-one-5,5,7,7-d_4 (267 mg, 1.5 mmol, 1.0 equiv.) in anhydrous THF (3 mL) was added dropwise to the reaction mixture. The reaction mixture was stirred for an additional 1.5 h at r.t. before quenched by the addition of 5 mL H_2O. The resulting mixture was extracted with ethyl acetate and the combined organic layers were dried over Na_2SO_4, filtered, concentrated *in vacuo* and purified by silica gel chromatography to get **2w**-d_4 (> 97% D) as a colorless oil.

^{1}H NMR (400 MHz, $CDCl_3$) δ 4.70 (s, 2H), 1.45 – 1.38 (m, 4H), 1.38 – 1.23 (m, 8H), 0.91 (t, J = 7.0 Hz, 6H).
^{13}C NMR (101 MHz, $CDCl_3$) δ 150.09, 108.28, 35.33 (quintet, J = 19.2 Hz), 31.50, 27.22, 22.48, 13.90.

HRMS (EI) calculated for $C_{12}H_{20}D_4$: 172.2129, found: 172.2124;

FTIR (cm^{-1}): 2956.3, 2922.4, 2858.9, 1637.2, 1465.8, 1377.9, 881.5.

II-B. General Procedures for Cobalt-Catalyzed Intermolecular Oxidative Hydroamidation

Evaluation of reaction conditions (Figure 2, "Standard Conditions"): An oven-dried 10 mL re-sealable screw-cap vial equipped with a Teflon-coated magnetic stir bar was charged with Co catalyst **1a** (6.0 mg, 0.010 mmol, 0.10 equiv.) and oxone (100 mesh, 123 mg, 0.20 mmol, 2.0 equiv.). The reaction vessel was then briefly evacuated and backfilled

with nitrogen (this sequence was repeated a total of three times). Anhydrous acetonitrile (1.0 mL), **2a** (18.1 mg, 0.10 mmol, 1.0 equiv.), degassed H_2O (2 μL, 0.11 mmol, 1.1 equiv.) and 1,1,3,3-tetramethyldisiloxane (73 μL, 0.40 mmol, 4.0 equiv.) were added to the reaction vessel via syringe sequentially. The reaction mixture was stirred at r.t. for 18 h before an NMR internal standard (phenanthrene) was added. The mixture was filtered through a short pad of silica gel with another 5 mL CH_2Cl_2/MeOH (20/1) as an eluent. The solvents were removed *in vacuo* and the residue was analyzed by ^{1}H NMR spectroscopy.

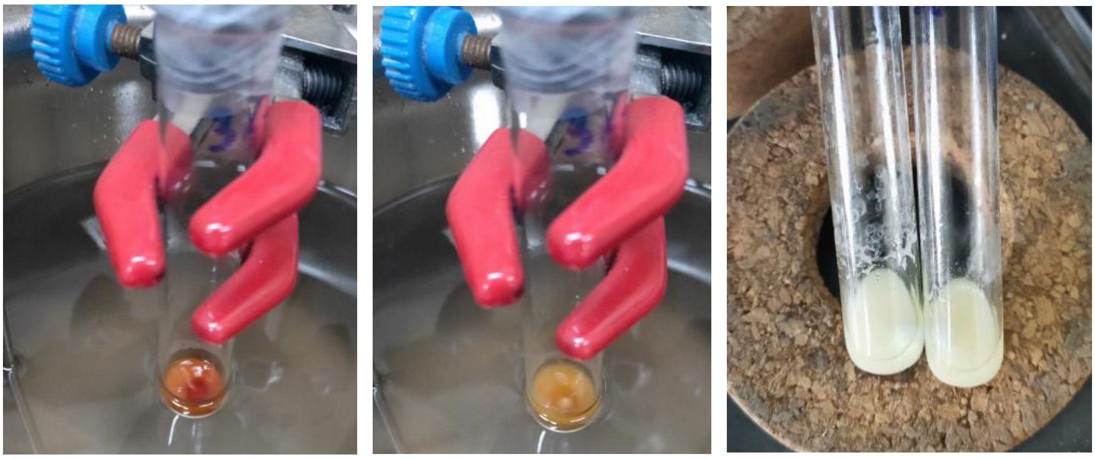

Supplementary Figure 1. Photos of the reaction mixture (Reaction time: 0 min, 5 min and 18 h)

General Procedure A: An oven-dried 25 mL re-sealable Schlenk tube equipped with a Teflon-coated magnetic stir bar was charged with Co catalyst **1a** (15 mg, 0.025 mmol, 0.050 equiv.) and oxone (100 mesh, 922 mg, 1.5 mmol, 3.00 equiv.). The reaction vessel was then briefly evacuated and backfilled with nitrogen (this sequence was repeated a total of three times). Anhydrous acetonitrile (5.0 mL), **2** (0.50 mmol, 1.0 equiv.), H_2O (20 μL, 1.1 mmol, 2.2 equiv.) and 1,1,3,3-tetramethyldisiloxane (275 μL, 1.5 mmol, 3.0 equiv.) were added to the reaction vessel via syringe sequentially. The reaction mixture was stirred at r.t. for 18 h. The mixture was filtered through a short pad of silica gel with CH_2Cl_2/MeOH (20/1) as an eluent. The solvents were removed *in vacuo* and the residue was purified by silica gel column chromatography to afford corresponding amide product **3**.

General Procedure B: An oven-dried 25 mL re-sealable schlenk tube equipped with a Teflon-coated magnetic stir bar was charged with Co catalyst **1a** (30 mg, 0.050 mmol, 0.10 equiv.) and oxone (100 mesh, 922 mg, 1.5 mmol, 3.0 equiv.). The reaction vessel was then briefly evacuated and backfilled with nitrogen (this sequence was repeated a total of three times). Anhydrous dichloromethane (5.0 mL), nitrile (1.0 mmol, 2.5 equiv.), **2** (0.50 mmol, 1.0 equiv.), H_2O (20 μL, 1.1 mmol, 2.2 equiv.) and 1,1,3,3-tetramethyldisiloxane (275 μL, 1.5 mmol, 3.0 equiv.) were added to the reaction vessel via syringe sequentially. The reaction mixture was stirred at r.t. for 18 h. The mixture was filtered through a short pad of silica gel with CH_2Cl_2/MeOH (20/1) as an eluent. The solvents were removed *in vacuo* and the residue was purified by silica gel column chromatography to afford corresponding amide product **8**.

General Procedure C: An oven-dried 25 mL re-sealable schlenk tube equipped with a Teflon-coated magnetic stir bar was charged with Co catalyst **1a** (30 mg, 0.050 mmol, 0.10 equiv.) and oxone (100 mesh, 922 mg, 1.5 mmol, 3.0 equiv.). The reaction vessel was then briefly evacuated and backfilled with nitrogen (this sequence was repeated a total of three times). Anhydrous dichloromethane (5.0 mL), nitrile (2.5 mmol, 5.0 equiv.), **2** (0.50 mmol, 1.0 equiv.), H_2O (20 μL, 1.1 mmol, 2.2 equiv.) and 1,1,3,3-tetramethyldisiloxane (275 μL, 1.5 mmol, 3.0 equiv.) were added to the reaction vessel via syringe sequentially. The reaction mixture was stirred at r.t. for 18 h. The mixture was filtered through a short pad of silica gel with CH_2Cl_2/MeOH (20/1) as an eluent. The solvents were removed *in vacuo* and the residue was purified by silica gel column chromatography to afford corresponding amide product **8**.

II-C. Characterizations of Compounds

Compound 3a (*N*-(2-methyldodecan-2-yl)acetamide)

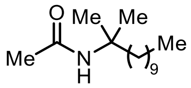

Following General Procedure A, the title compound was synthesized from 2-methyldodec-1-ene (**2a**) (90.2 mg, 0.50 mmol). The product was purified by silica gel flash column chromatography (PE: ethyl acetate = 10:1 to 1:1) to afford **3a** (114.0 mg, 94%) as a pale yellow solid. The spectroscopic data matches the literature reported.[17]

^{1}H NMR (400 MHz, $CDCl_3$) δ 5.19 (s, 1H), 1.91 (s, 3H), 1.69 – 1.58 (m, 2H), 1.32 – 1.22 (m, 22H), 0.87 (t, J = 6.8 Hz, 3H).

^{13}C NMR (101 MHz, $CDCl_3$) δ 169.39, 53.68, 40.34, 31.90, 30.01, 29.69, 29.65, 29.61, 29.33, 26.87, 24.48, 24.14, 22.67, 14.10.

Compound 3b (*N*-(5-methylundecan-5-yl)acetamide)

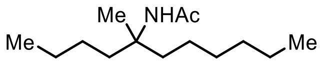

Following General Procedure A, the title compound was synthesized from 5-methyleneundecane (**2b**) (84.1 mg, 0.50 mmol). The product was purified by silica gel flash column chromatography (PE: ethyl acetate = 10:1 to 1:1) to afford **3b** (109.0 mg, 96%) as a pale yellow solid.

^{1}H NMR (400 MHz, $CDCl_3$) δ 5.30 (s, 1H), 1.86 (s, 3H), 1.67 (qd, J = 8.7, 3.6 Hz, 2H), 1.75 – 1.47 (m, 2H), 1.27 – 1.13 (m, 15H), 0.87 – 0.81 (m, 6H).

^{13}C NMR (101 MHz, $CDCl_3$) δ 169.31, 56.24, 38.33, 38.06, 31.87, 29.69, 25.90, 24.38, 24.35, 23.63, 23.08, 22.64, 14.13, 14.05.

FTIR (cm^{-1}): 3296.5, 2954.8, 2926.5, 2858.7, 2359.0, 1646.8, 1552.2, 1465.9, 1299.3;

HRMS (ESI) $[M + H]^+$ calculated for $C_{14}H_{30}NO$: 228.2322, found: 228.2321;

TLC: R_f = 0.40 (PE: ethyl acetate = 1:1).

Compound 3c (*N*-((1*r*,3*r*,5*r*,7*r*)-2-methyladamantan-2-yl)acetamide)

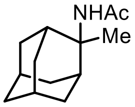

Following General Procedure A, the title compound was synthesized from 2-methylideneadamantane (**2c**) (75.0 mg, 0.50 mmol). The product was purified by silica gel flash column chromatography (PE: ethyl acetate = 10:1 to 1:1) to afford **3c** (98.5 mg, 95%) as a pale yellow solid. The spectroscopic data matches the literature reported.[18]

^1H NMR (400 MHz, $CDCl_3$) δ 5.40 (s, 1H), 2.12 (s, 2H), 1.90 – 1.97 (m, 7H), 1.77 (s, 2H), 1.70 – 1.59 (m, 4H), 1.59 – 1.52 (m, 2H), 1.47 (s, 3H).

^{13}C NMR (101 MHz, $CDCl_3$) δ 168.81, 58.08, 38.12, 34.92, 32.90, 32.79, 27.14, 26.50, 24.21, 22.68.

Compound 3d (*N*-(4-butyl-2,2-dimethyltetrahydro-2H-pyran-4-yl)acetamide)

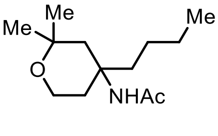

Following General Procedure A, the title compound was synthesized from 4-butylidene-2,2-dimethyltetrahydro-2H-pyran (**2d**) (85.0 mg, 0.50 mmol). The product was purified by silica gel flash column chromatography (PE: ethyl acetate = 10:1 to 1:1) to afford **3d** (74.4 mg, 65%) as a pale yellow oil.

^1H NMR (400 MHz, $CDCl_3$) δ 5.53 (s, 1H), 3.75 (td, J = 12.3, 2.2 Hz, 1H), 3.61 (ddd, J = 12.4, 4.9, 2.4 Hz, 1H), 2.34 (dd, J = 14.5, 2.3 Hz, 1H), 1.97 – 1.76 (m, 5H), 1.53 – 1.35 (m, 2H), 1.31 – 1.03 (m, 11H), 0.83 (t, J = 7.2 Hz, 3H).

^{13}C NMR (101 MHz, $CDCl_3$) δ 169.57, 70.87, 57.27, 53.54, 42.88, 39.36, 34.79, 32.07, 24.69, 24.18, 22.97, 22.54, 13.84.

FTIR (cm^{-1}): 3294.7, 2928.6, 1647.8, 1551.9, 1367.8, 1301.0, 1116.8, 1040.6, 961.4;

HRMS (ESI) $[M + H]^+$ calculated for $C_{13}H_{26}NO_2$: 228.1958, found: 228.1963;

TLC: R_f = 0.10 (PE: ethyl acetate = 1:1).

Compound 3e (*N*-(1-methylcyclopentyl)acetamide)

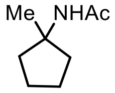

Following General Procedure A, the title compound was synthesized from 1-methylcyclopent-1-ene (**2e**) (42.0 mg, 0.50 mmol). The product was purified by silica gel flash column chromatography (PE: ethyl acetate = 10:1 to 1:1) to afford **3e** (60.3 mg, 86%) as a pale yellow solid. The spectroscopic data matches the literature reported.[19]

^{1}H NMR (400 MHz, $CDCl_3$) δ 5.66 (s, 1H), 1.95 – 1.86 (m, 5H), 1.69 – 1.56 (m, 6H), 1.36 (s, 3H).

^{13}C NMR (101 MHz, $CDCl_3$) δ 169.69, 61.19, 39.35, 25.44, 24.31, 23.69.

Compound 3f (*N*-(1-methylcyclohexyl)acetamide)

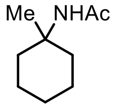

Following General Procedure A, the title compound was synthesized from 1-methylcyclopent-1-ene (**2f**) (49.0 mg, 0.50 mmol). The product was purified by silica gel flash column chromatography (PE: ethyl acetate = 10:1 to 1:1) to afford **3f** (53.6 mg, 69%) as a pale yellow solid. The spectroscopic data matches the literature reported.[20]

^{1}H NMR (400 MHz, $CDCl_3$) δ 5.26 (s, 1H), 2.01 – 1.87 (m, 5H), 1.58 – 1.17 (m,11H).

^{13}C NMR (101 MHz, $CDCl_3$) δ 169.21, 53.04, 36.37, 25.86, 25.27, 24.32, 21.72.

Compound 3h

(*N*-((1s,4s)-4-(2-hydroxypropan-2-yl)-1-methylcyclohexyl)acetamide)

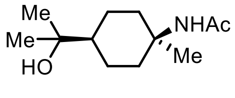

Following General Procedure A, the title compound was synthesized from alpha-terpineol (**2h**) (78.0 mg, 0.50 mmol). Diastereomeric ratio (3:1) was determined by ^{1}H NMR analysis of the crude reaction mixture. The product was purified by silica gel flash column chromatography (PE: ethyl acetate = 10:1 to 1:1) to afford **3h** (57.7 mg, 54%, mixture of diastereoisomers) as a colorless solid. The stereochemistry of the major diastereomer (shown above) was determined by X-ray single crystal diffraction of a recrystallized sample.

^{1}H NMR (400 MHz, $CDCl_3$) δ 5.27 (s, 1H), 2.26 – 2.19 (m, 2H), 1.90 (s, 3H), 1.62 (d, *J* = 9.5 Hz, 2H), 1.30 (s, 3H), 1.22 – 1.08 (m, 12H).

^{13}C NMR (101 MHz, $CDCl_3$) δ 169.46, 72.04, 52.43, 47.86, 36.17, 27.22, 26.80, 24.20, 22.28.

FTIR (cm^{-1}): 3304.8, 2933.6, 1650.6, 1548.7, 1445.7, 1371.6, 1180.4, 1133.5;

HRMS (ESI) $[M + H]^+$ calculated for $C_{12}H_{24}NO_2$: 214.1802, found: 214.1800;

TLC: R_f = 0.05 (PE: ethyl acetate = 1:1).

Compound 3i

(*rac*-*N*-((1*R*,2*R*,3*S*,5*S*,7*S*)-5-hydroxy-2-methyladamantan-2-yl)acetamide)

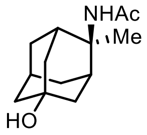

Following General Procedure A, the title compound was synthesized from *rac*-(1*s*,3*R*,5*S*,7*s*)-4-methyleneadamantan-1-ol (**2i**) (82.2 mg, 0.50 mmol). Diastereomeric ratio (5:1) was determined by ^{1}H NMR analysis of the crude reaction mixture. The product was purified by silica gel flash column chromatography (PE: ethyl acetate = 5:1 to 1:1) to afford the major diastereomer **3i** (71.5 mg, 64%, single diastereoisomer) as a pale yellow solid. The stereochemistry of the major diastereomer (shown above) was determined by X-ray single crystal diffraction of a recrystallized sample.

^{1}H NMR (400 MHz, $CDCl_3$) δ 5.28 (s, 1H), 2.61 (s, 1H), 2.37 (s, 2H), 2.16 (d, *J* = 3.1 Hz, 1H), 2.09 (p, *J* = 3.1 Hz, 1H), 1.98 – 1.93 (m, 5H), 1.91 – 1.84 (m, 2H), 1.68 (s, 2H), 1.54 – 1.50 (m, 1H), 1.48 (s, 3H), 1.23 (s, 2H).

^{13}C NMR (101 MHz, $CDCl_3$) δ 169.32, 69.55, 67.24, 57.25, 53.82, 46.11, 40.61, 37.77, 31.79, 29.73, 29.28, 24.58, 22.19.

FTIR (cm^{-1}): 3290.9, 2989.6, 2920.3, 1634.4, 1557.2, 1358.9, 1304.1, 1114.3, 1084.5

HRMS (ESI) $[M + H]^+$ calculated for $C_{13}H_{22}NO_2$: 224.1645, found: 224.1644;

TLC: R_f = 0.45 (PE: acetone = 1:1).

Compound 3j

***N*-(2-methyl-4-(((8R,9S,13S)-13-methyl-17-oxo-7,8,9,11,12,13,14,15,16,17-decahydro-6H-cyclopenta[a]phenanthren-3-yl)oxy)butan-2-yl)acetamide**

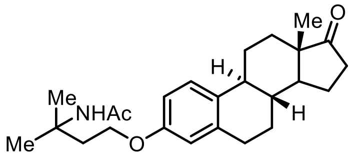

Following General Procedure A, the title compound was synthesized from *O*-3-methylbut-3-en-1-yl-estrone (**2j**) (169.3 mg, 0.50 mmol). The product was purified by silica gel flash column chromatography (PE: ethyl acetate = 5:1 to 1:1) to afford **3j** (184.9 mg, 93%) as a pale yellow solid.

^1H NMR (400 MHz, $CDCl_3$) δ 7.16 (d, *J* = 8.6 Hz, 1H), 6.67 (dd, *J* = 8.6, 2.7 Hz, 1H), 6.60 (s, 1H), 6.27 (s, 1H), 4.02 (t, *J* = 5.9 Hz, 2H), 2.86 (q, *J* = 5.1, 4.1 Hz, 2H), 2.46 (dd, *J* = 18.7, 8.7 Hz, 1H), 2.33 (dd, *J* = 9.3, 4.5 Hz, 1H), 2.24 – 1.83 (m, 10H), 1.71 – 1.15 (m, 12H), 0.86 (s, 3H).

^{13}C NMR (101 MHz, $CDCl_3$) δ 220.96, 169.61, 156.50, 137.94, 132.40, 126.49, 114.33, 112.05, 64.85, 53.26, 50.40, 48.02, 43.98, 39.65, 38.36, 35.89, 31.58, 29.69, 27.09, 27.07, 26.53, 25.93, 24.64, 21.60, 13.87.

FTIR (cm^{-1}): 2927.2, 1733.5, 1655.1, 1608.3, 1541.0, 1498.2, 1452.5, 1367.5, 1280.4;

HRMS (ESI) $[M + H]^+$ calculated for $C_{25}H_{36}NO_3$: 398.2690, found: 398.2687;

TLC: R_f = 0.30 (PE: ethyl acetate = 1:1).

Compound 3k (6-acetamido-6-methylheptanoic acid)

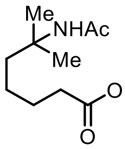

Following General Procedure A, the title compound was synthesized from 6-methylhept-6-enoic acid (**2k**) (71.1 mg, 0.50 mmol). The product was purified by silica

gel flash column chromatography (PE: ethyl acetate = 5:1 to 1:1) to afford **3k** (83.5 mg, 83%) as a pale yellow solid.

^{1}H NMR (400 MHz, CDCl$_3$) δ 10.75 (br, 1H), 5.48 (s, 1H), 2.33 (t, J = 7.3 Hz, 2H), 1.92 (s, 3H), 1.73 – 1.66 (m, 2H), 1.61 (p, J = 7.5 Hz, 2H), 1.34 – 1.21 (m, 9H).

^{13}C NMR (101 MHz, CDCl$_3$) δ 178.37, 170.30, 53.85, 39.46, 34.00, 26.95, 24.94, 24.28, 23.51.

FTIR (cm^{-1}): 2929.3, 1707.3, 1623.5, 1548.5, 1370.3, 1126.0, 1038.3, 738.0, 610.1;

HRMS (ESI) [M + H]$^+$ calculated for $C_{10}H_{20}NO_3$: 202.1438, found: 202.1437;

TLC: R_f = 0.30 (PE: acetone = 1:1).

Compound 3l (3-acetamido-3-methylbutyl (*E*)-oct-3-enoate)

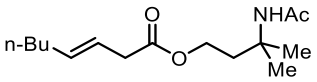

Following General Procedure A, the title compound was synthesized from 3-methylbut-3-en-1-yl (*E*)-oct-3-enoate (**2l**) (105.2 mg, 0.50 mmol). The product was purified by silica gel flash column chromatography (PE: ethyl acetate = 10:1 to 1:1) to afford **3l** (118.2 mg, 88%) as a pale yellow solid.

^{1}H NMR (400 MHz, CDCl$_3$) δ 5.61 – 5.42 (m, 3H), 4.11 (t, J = 6.8 Hz, 2H), 2.98 (d, J = 6.1 Hz, 2H), 2.07 (t, J = 6.8 Hz, 2H), 2.00 (q, J = 6.6 Hz, 2H), 1.90 (s, 3H), 1.38 – 1.21 (m, 12H), 0.86 (t, J = 6.9 Hz, 3H).

^{13}C NMR (101 MHz, CDCl$_3$) δ 172.03, 169.45, 134.84, 121.00, 61.28, 52.48, 37.99, 37.70, 31.91, 31.05, 27.13, 24.18, 21.93, 13.67.

FTIR (cm^{-1}): 3304.15, 2926.4, 1734.9, 1652.4, 1549.5, 1450.0, 1161.5, 968.3, 608.9;

HRMS (ESI) [M + H]$^+$ calculated for $C_{15}H_{28}NO_3$: 270.2064, found: 270.2062;

TLC: R_f = 0.50 (PE: ethyl acetate = 1:1).

Compound 3m

(*N*-(2-methyl-4-(2,6,6-trimethylcyclohex-1-en-1-yl)butan-2-yl)acetamide)

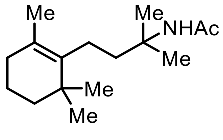

Following General Procedure A, the title compound was synthesized from 1,3,3-trimethyl-2-(3-methylbut-3-en-1-yl)cyclohex-1-ene (**2m**) (96.2 mg, 0.50 mmol). The product was purified by silica gel flash column chromatography (PE: ethyl acetate = 10:1 to 1:1) to afford **3m** (112.6 mg, 90%) as a pale yellow solid.

^{1}H NMR (400 MHz, $CDCl_3$) δ 5.48 (s, 1H), 1.95 – 1.83 (m, 7H), 1.76 – 1.67 (m, 2H), 1.58 – 1.50 (m, 5H), 1.40 – 1.33 (m, 2H), 1.29 (s, 6H), 0.93 (s, 6H).

^{13}C NMR (101 MHz, $CDCl_3$) δ 169.37, 136.33, 127.14, 53.87, 39.87, 39.85, 34.99, 32.79, 28.68, 26.71, 24.40, 22.95, 19.72, 19.50.

FTIR (cm^{-1}): 3292.4, 3077.9, 2925.1, 1644.6, 1550.9, 1472.9, 1303.17, 1176.04, 606.3;

HRMS (ESI) $[M + H]^+$ calculated for $C_{16}H_{30}NO$: 252.2322, found: 252.2321;

TLC: R_f = 0.70 (PE: ethyl acetate = 1:1).

Compound 3n (Diethyl (3*S*,4*R*)-3-(2-acetamidopropan-2-yl)-4-methylcyclopentane-1,1-dicarboxylate)

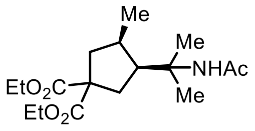

Following General Procedure A, the title compound was synthesized from diethyl (3*S*,4*R*)-3-(2-acetamidopropan-2-yl)-4-methylcyclopentane-1,1-dicarboxylate (**2n**) (134.2 mg, 0.50 mmol). The product was purified by silica gel flash column chromatography (PE: ethyl acetate = 5:1 to 1:1) to afford **3n** (78.6 mg, 48%, single diastereoisomer) as a pale yellow solid. The stereochemistry of the major diastereomer was assigned by analogy with a structurally related literature compound.[21]

^{1}H NMR (400 MHz, $CDCl_3$) δ 5.36 (s, 1H), 4.21 – 4.10 (m, 4H), 2.44 – 2.26 (m, 4H), 2.19 (d, J = 13.1 Hz, 1H), 2.03 (d, J = 13.9 Hz, 1H), 1.89 (s, 3H), 1.39 (s, 6H), 1.22 (t, J = 7.1 Hz, 6H), 0.93 (d, J = 7.0 Hz, 3H).

^{13}C NMR (101 MHz, $CDCl_3$) δ 173.07, 172.71, 169.25, 61.54, 61.51, 57.78, 55.00, 51.31, 41.89, 34.88, 32.67, 26.28, 26.02, 24.72, 17.10, 14.02.

FTIR (cm^{-1}): 3309.2, 2977.0, 1724.4, 1653.2, 1544.5, 1445.2, 1365.7, 1273.8, 1248.5;

HRMS (ESI) $[M + H]^+$ calculated for $C_{17}H_{30}NO_5$: 328.2118, found: 328.2119;

TLC: R_f = 0.2 (PE: ethyl acetate = 1:1).

Compound 3o

(*N*-(4-((3,7-dimethyloct-6-en-1-yl)oxy)-2-methylbutan-2-yl)acetamide)

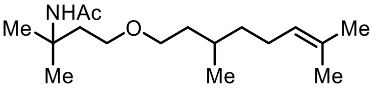

Following General Procedure A, the title compound was synthesized from 2,6-dimethyl-8-((3-methylbut-3-en-1-yl)oxy)oct-2-ene (**2o**) (112.2 mg, 0.50 mmol). The product was purified by silica gel flash column chromatography (PE: ethyl acetate = 10:1 to 1:1) to afford **3o** (75.1 mg, 53%) as a pale yellow solid.

^{1}H NMR (400 MHz, $CDCl_3$) δ 6.65 (s, 1H), 5.05 (dddd, *J* = 8.6, 5.7, 2.8, 1.3 Hz, 1H), 3.58 (t, *J* = 6.5 Hz, 2H), 3.41 (t, *J* = 6.7 Hz, 2H), 2.04 – 1.86 (m, 2H), 1.83 (s, 3H), 1.77 (t, *J* = 6.3 Hz, 2H), 1.68 – 1.42 (m, 8H), 1.41 – 1.21 (m, 8H), 1.19 – 1.07 (m, 1H), 0.90 (d, *J* = 6.6 Hz 3H).

^{13}C NMR (101 MHz, $CDCl_3$) δ 169.12, 130.95, 124.30, 69.16, 67.40, 52.96, 41.11, 36.88, 36.48, 29.18, 25.98, 25.94, 25.40, 25.10, 24.26, 19.15, 17.31.

FTIR (cm^{-1}): 3309.6, 2923.1, 1653.9, 1544.3, 1453.8, 1370.5, 1275.4, 1258.9, 1107.3;

HRMS (ESI) $[M + H]^+$ calculated for $C_{17}H_{34}NO_2$: 284.2584, found: 284.2586;

TLC: R_f = 0.75 (PE: ethyl acetate = 1:1).

Compound 3o' (*N*, *N'*-(2,6-dimethyloctane-2,6-diyl)diacetamide)

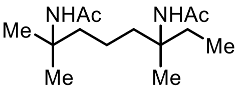

Adapted from a literature procedure.[22] An oven-dried 10 mL re-sealable screw-cap vial equipped with a Teflon-coated magnetic stir bar was charged with concentrated sulfuric acid (1 mL) and glacial acetic acid (2 mL). MeCN (1 mL) was added at 0 °C. Then **2o** (112.2 mg, 0.5 mmol in 1 mL MeCN) was added dropwise. The mixture was stirred for 5 h at r.t. before quenched with 5 mL H_2O. The pH of reaction mixture was adjusted to 7 – 8 with saturated aqueous Na_2CO_3 (10 mL) and extracted with diethyl ether (3 × 10 mL). The combined organic layers were dried over Na_2SO_4, filtered, concentrated *in vacuo* and purified via column chromatography (PE: ethyl acetate = 1:1) to yield **3o'** (19.0 mg, 15%) as a pale yellow solid.

^{1}H NMR (400 MHz, $CDCl_3$) δ 5.38 (s, 1H), 5.22 (s, 1H), 1.91 (s, 3H), 1.90 (s, 3H), 1.84 – 1.47 (m, 5H), 1.30 – 1.16 (m, 12H), 0.79 (t, *J* = 7.4 Hz, 3H).

^{13}C NMR (101 MHz, $CDCl_3$) δ 169.30, 169.25, 56.33, 53.49, 40.29, 37.96, 30.58, 26.70, 26.61, 24.22, 24.17, 23.62, 17.94, 7.73.

FTIR (cm^{-1}): 3295.5, 2988.2, 1645.6, 1551.6, 1458.6, 1275.4, 1258.9, 749.8, 479.0, 434.7;

HRMS (ESI) $[M + H]^+$ calculated for $C_{14}H_{29}N_2O_2$: 257.2224, found: 257.2222;

TLC: R_f = 0.30 (PE: ethyl acetate = 1:1).

Compound 3p (*N*-(8-methyl-1,4-dioxaspiro[4.5]decan-8-yl)acetamide)

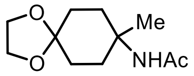

Following a slightly modified General Procedure A where additional 2.0 equiv. 2,6-lutidine was added. The title compound was synthesized from 8-methylene-1,4-dioxaspiro[4.5]decane (**2p**) (78.0 mg, 0.50 mmol). The product was purified by silica gel flash column chromatography (PE: ethyl acetate = 10:1 to 1:1) to afford **3p** (90.6 mg, 85%) as a pale yellow solid.

^1H NMR (400 MHz, $CDCl_3$) δ 5.23 (s, 1H), 3.93 (s, 4H), 2.12 – 2.05 (m, 2H), 1.93 (s, 3H), 1.72 – 1.52 (m, 6H), 1.38 (s, 3H).

^{13}C NMR (101 MHz, $CDCl_3$) δ 169.92, 108.13, 64.29, 64.28, 52.52, 34.10, 30.74, 24.57.

FTIR (cm^{-1}): 3303.0, 2930.9, 1643.9, 1544.5, 1443.7, 1365.2, 1300.7, 1095.0, 937.0;

HRMS (ESI) $[M + H]^+$ calculated for $C_{11}H_{20}NO_3$: 214.1438, found: 214.1437;

TLC: R_f = 0.10 (PE: ethyl acetate = 1:1).

Compound 3q (tert-butyl 4-(2-acetamidopropan-2-yl)piperidine-1-carboxylate)

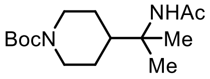

Following General Procedure A, the title compound was synthesized from tert-butyl 4-(prop-1-en-2-yl)piperidine-1-carboxylate (**2q**) (113.0 mg, 0.50 mmol). The product was purified by silica gel flash column chromatography (PE: ethyl acetate = 10:1 to 1:1) to afford **3q** (128.2 mg, 90%) as a pale yellow solid.

^1H NMR (400 MHz, $CDCl_3$) δ 5.47 (s, 1H), 4.10 (s, 2H), 2.59 (t, J = 13.0 Hz, 2H), 2.24 (tt, J = 12.3, 3.3 Hz, 1H), 1.87 (s, 3H), 1.56 (d, J = 12.9, 2H), 1.39 (s, 9H), 1.20 (s, 6H), 1.10 (td, J = 12.6, 4.4 Hz, 2H).

^{13}C NMR (101 MHz, $CDCl_3$) δ 169.46, 154.70, 79.27, 56.09, 42.15, 28.44, 26.78, 24.42, 24.25.

FTIR (cm^{-1}): 3311.9, 2972.6, 2942.9, 2855.0, 1654.8, 1547.4, 1424.8, 1364.9, 1243.3;

HRMS (ESI) $[M + H]^+$ calculated for $C_{15}H_{29}N_2O_3$: 285.2173, found: 285.2173;

TLC: R_f = 0.05 (PE: ethyl acetate = 1:1).

Compound 3r (*N*-(2-((1*R*,4*S*)-4-methyl-3-oxocyclohexyl)propan-2-yl)acetamide)

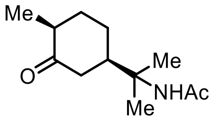

Following General Procedure A, the title compound was synthesized from (+)-dihydrocarvone (**2r**) (77.0 mg, 0.50 mmol). The product was purified by silica gel flash column chromatography (PE: ethyl acetate = 10:1 to 1:1) to afford **3r** (100.4 mg, 95%) as a pale yellow solid.

^{1}H NMR (400 MHz, $CDCl_3$) δ 5.77 (s, 1H), 2.48 (ddt, *J* = 13.6, 12.1, 3.4 Hz, 1H), 2.31 (ddd, *J* = 13.0, 3.7, 2.4 Hz, 1H), 2.21 (tdd, *J* = 12.8, 6.4, 1.5 Hz, 1H), 2.07 – 1.94 (m, 2H), 1.83 (s, 3H), 1.81 – 1.72 (m, 1H), 1.38 (qd, *J* = 12.7, 3.4 Hz, 1H), 1.30 – 1.10 (m, 7H), 0.91 (d, *J* = 6.5 Hz, 3H).

^{13}C NMR (101 MHz, $CDCl_3$) δ 212.32, 169.21, 55.44, 45.21, 44.30, 42.85, 33.89, 26.10, 23.93, 23.91, 23.61, 13.90.

FTIR (cm^{-1}): 3307.0, 2969.9, 1705.7, 1652.3, 1541.6, 1449.1, 1367.0, 966.8, 603.7;

HRMS (ESI) $[M + H]^+$ calculated for $C_{12}H_{22}NO$: 212.1645, found: 212.1645;

TLC: R_f = 0.05 (PE: ethyl acetate = 1:1).

Compound 3s

(*N*-(2-((1*S*,3*S*,6*S*)-6-methyl-5-oxo-7-oxabicyclo[4.1.0]heptan-3-yl)propan-2-yl)acetamide)

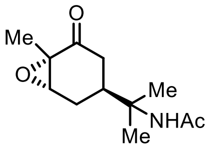

Following a slightly modified General Procedure A where additional 2.0 equiv.

2,6-lutidine was added. The title compound was synthesized from (1*S*,4*S*,6*S*)-1-methyl-4-(prop-1-en-2-yl)-7-oxabicyclo[4.1.0]heptan-2-one (**2s**) (83.1 mg, 0.50 mmol). The product was purified by silica gel flash column chromatography (PE: ethyl acetate = 10:1 to 1:1) to afford **3s** (48.0 mg, 43%) as a pale yellow solid.

^{1}H NMR (400 MHz, $CDCl_3$) δ 5.25 (s, 1H), 3.42 (d, J = 3.3 Hz, 1H), 2.83 – 2.70 (m, 1H), 2.50 (ddd, J = 17.9, 5.3, 1.5 Hz, 1H), 2.39 – 2.26 (m, 1H), 1.91 (s, 4H), 1.77 (ddd, J = 14.7, 12.0, 0.9 Hz, 1H), 1.38 (s, 3H), 1.30 (s, 3H), 1.24 (s, 3H).

^{13}C NMR (101 MHz, $CDCl_3$) δ 206.71, 170.05, 61.74, 59.33, 55.87, 39.01, 35.40, 25.40, 25.19, 24.93, 24.67, 15.58.

FTIR (cm^{-1}): 3307.8, 2974.1, 2931.6, 2359.2, 2342.5, 1702.4, 1652.6, 1540.9, 1367.5;

HRMS (ESI) [M + H]$^+$ calculated for $C_{12}H_{20}NO_3$: 226.1438, found: 226.1436;

TLC: R_f = 0.80 (PE: acetone = 1:1).

Compound 3t (3-acetamido-3-methylbutyl 2-(6-chloropyridin-3-yl)acetate)

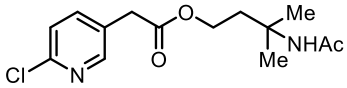

Following General Procedure A, the title compound was synthesized from 3-methylbut-3-en-1-yl 2-(6-chloropyridin-3-yl)acetate (**2t**) (119.9 mg, 0.50 mmol). The product was purified by silica gel flash column chromatography (PE: ethyl acetate = 10:1 to 1:1) to afford **3t** (134.2 mg, 90%) as a pale yellow solid.

^{1}H NMR (400 MHz, $CDCl_3$) δ 8.16 (d, J = 2.5 Hz, 1H), 7.52 (dd, J = 8.2, 2.5 Hz, 1H), 7.20 (d, J = 8.2 Hz, 1H), 5.88 (s, 1H), 4.05 (t, J = 7.0 Hz, 2H), 3.50 (s, 2H), 2.03 (t, J = 7.0 Hz, 2H), 1.79 (s, 3H), 1.19 (s, 6H).

^{13}C NMR (101 MHz, $CDCl_3$) δ 169.81, 169.38, 149.70, 149.55, 139.40, 128.23, 123.63, 61.89, 51.93, 37.03, 36.90, 26.95, 23.72.

FTIR (cm^{-1}): 2970.0, 1731.5, 1650.9, 1587.8, 1566.5, 1384.8, 1355.3

HRMS (ESI) [M + H]$^+$ calculated for $C_{14}H_{20}ClN_2O_3$: 299.1157, found: 299.1150;

TLC: R_f = 0.05 (PE: ethyl acetate = 1:1).

Compound 3u (3-acetamido-3-methylbutyl thiophene-2-carboxylate)

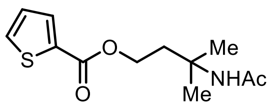

Following General Procedure A, the title compound was synthesized from 3-methylbut-3-en-1-yl thiophene-2-carboxylate (**2u**) (98.1 mg, 0.50 mmol). The product was purified by silica gel flash column chromatography (PE: ethyl acetate = 10:1 to 1:1) to afford **3u** (121.4 mg, 95%) as a pale yellow solid.

^{1}H NMR (400 MHz, CDCl$_3$) δ 7.69 (d, J = 3.8 Hz, 1H), 7.48 (d, J = 5.0 Hz, 1H), 7.01 (t, J = 5.0 Hz, 1H), 6.01 (s, 1H), 4.27 (t, J = 6.6 Hz, 2H), 2.16 (t, J = 6.6 Hz, 2H), 1.84 (s, 3H), 1.31 (s, 6H).

^{13}C NMR (101 MHz, CDCl$_3$) δ 173.75, 57.89, 40.77, 38.43, 35.33, 33.23, 33.12, 27.46, 26.82, 25.33, 23.10, 17.96.

FTIR (cm^{-1}): 2988.0, 2921.3, 1651.2, 1649.8, 1550.4, 1525.4, 1418.0, 1375.8

HRMS (ESI) [M + H]$^+$ calculated for $C_{12}H_{18}NO_3S$: 256.1002, found: 256.1000;

TLC: R_f = 0.20 (PE: ethyl acetate = 1:1).

Compound 3v

(*N*-(2-((1*S*,2*R*,4*R*)-2-hydroxy-4-methylcyclohexyl)propan-2-yl)acetamide)

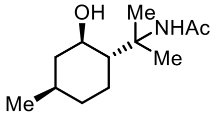

Following a slightly modified General Procedure A where 5 mL aqueous NaOH (1 M) was added during workup. The mixture was stirred for additional 10 minutes and extracted with dichloromethane (3 × 15 mL). The title compound was synthesized from (-)-isopulegol (**2v**) (78.0 mg, 0.50 mmol). The product was purified by silica gel flash column chromatography (PE: ethyl acetate = 10:1 to 1:1) to afford **3v** (101.3 mg, 95%) as a pale yellow solid. The spectroscopic data matches the literature reported.[22]

^{1}H NMR (400 MHz, CDCl$_3$) δ 7.45 (s, 1H), 3.66 (td, J = 10.4, 4.4 Hz, 1H), 3.10 (s, 1H), 1.91 (dtd, J = 12.2, 3.9, 2.2 Hz, 1H), 1.86 (s, 3H), 1.82 – 1.72 (m, 1H), 1.68 – 1.61 (m, 1H), 1.46 (s, 4H), 1.30 (s, 3H), 1.26 (s, 2H), 1.11 – 1.06 (m, 1H), 0.89 (d, J = 6.5 Hz, 4H).

^{13}C NMR (101 MHz, CDCl$_3$) δ 169.54, 72.05, 56.64, 51.37, 45.60, 34.23, 31.22, 25.36, 24.84, 21.69, 21.27.

Compound 6 (4-decyl-2,4-dimethyl-4,5-dihydrooxazole)

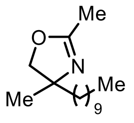

6

Compound **6** detected by ^{1}H NMR spectroscopic analysis of the crude reaction mixture (ca. 10%). The structure was assigned based on analogy with literature compounds.[23]

Characteristic ^{1}H NMR signals (protonated form): 4.55 (d, *J* = 9.3 Hz, 1H), 4.48 (d, *J* = 9.3 Hz, 1H), 2.50 (s, 3H), 1.04 (s, 3H).[22]

Compound 7a

(±)- (*N*-((1*R*,3*R*,5*R*,7*R*)-2-methyladamantan-2-yl)cyclobutanecarboxamide)

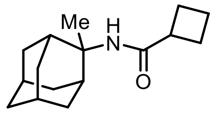

Following General Procedure B, the title compound was synthesized from 2-methylideneadamantane (**2c**) (75.0 mg, 0.50 mmol) and cyanocyclobutane (81.0 mg, 1.0 mmol). The product was purified by silica gel flash column chromatography (PE: ethyl acetate = 50:1 to 10:1) to afford **7a** (86.6 mg, 70%) as a pale yellow solid.

^{1}H NMR (400 MHz, $CDCl_3$) δ 5.16 (s, 1H), 2.93 (p, *J* = 8.5 Hz, 1H), 2.26 – 2.15 (m, 2H), 2.14 – 2.03 (m, 4H), 1.95 – 1.73 (m, 8H), 1.66 – 1.52 (m, 6H), 1.45 (s, 3H).

^{13}C NMR (101 MHz, $CDCl_3$) δ 173.47, 57.61, 40.49, 38.15, 35.05, 32.95, 32.85, 27.18, 26.55, 25.05, 22.82, 17.68.

FTIR (cm^{-1}): 3316.8, 2896.0, 2856.4, 1637.6, 1540.6, 1446.2, 1273.4, 1222.8, 1124.4;

HRMS (ESI) $[M + H]^+$ calculated for $C_{16}H_{26}NO$: 248.2009, found: 248.2005;

TLC: R_f = 0.90 (PE: ethyl acetate = 2:1).

Compound 7b

(±)- (*N*-((1*R*,3*R*,5*R*,7*R*)-2-methyladamantan-2-yl)cyclohexanecarboxamide)

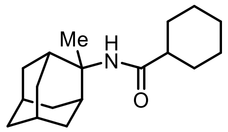

Following General Procedure B, the title compound was synthesized from 2-methylideneadamantane (**2c**) (75.0 mg, 0.50 mmol) and cyclohexanecarbonitrile (109.2 mg, 1.0 mmol). The product was purified by silica gel flash column chromatography (PE: ethyl acetate = 50:1 to 10:1) to afford **7b** (89.5 mg, 65%) as a pale yellow solid.

^{1}H NMR (400 MHz, $CDCl_3$) δ 5.26 (s, 1H), 2.12 (s, 2H), 2.03 – 1.87 (m, 5H), 1.84 – 1.71 (m, 6H), 1.66 – 1.53 (m, 7H), 1.44 (m, 2H), 1.40 – 1.10 (m, 6H).

^{13}C NMR (101 MHz, $CDCl_3$) δ 174.92, 57.82, 46.50, 38.44, 35.32, 33.24, 33.15, 29.90, 27.48, 26.85, 25.80, 23.12.

FTIR (cm^{-1}): 3316.6., 2918.4, 2854.0, 1645.3, 1533.5, 1448.3, 1368.4, 1211.3, 959.6;

HRMS (ESI) [M + H]$^+$ calculated for $C_{18}H_{30}NO$: 276.2322, found: 276.2322;

TLC: R_f = 0.80 (PE: ethyl acetate = 5:1).

Compound 7c

(±)- (*N*-((1*R*,3*R*,5*R*,7*R*)-2-methyladamantan-2-yl)-2-(naphthalen-1-yl)acetamide)

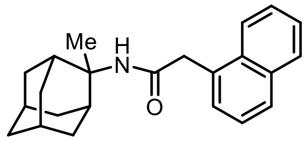

Following General Procedure B, the title compound was synthesized from 2-methylideneadamantane (**2c**) (75.0 mg, 0.50 mmol) and 1-naphthyl acetonitrile (167.2 mg, 1.0 mmol). The product was purified by silica gel flash column chromatography (PE: ethyl acetate = 50:1 to 10:1) to afford **7c** (96.7 mg, 58%) as a pale yellow solid.

^{1}H NMR (400 MHz, $CDCl_3$) δ 8.05 – 7.99 (m, 1H), 7.89 – 7.82 (m, 1H), 7.80 (d, J = 8.1 Hz, 1H), 7.55 – 7.47 (m, 2H), 7.47 – 7.37 (m, 2H), 5.09 (s, 1H), 3.98 (s, 2H), 1.92 – 1.77 (m, 4H), 1.68 (p, J = 3.1 Hz, 1H), 1.54 – 1.44 (m, 4H), 1.40 (s, 3H), 1.32 – 1.16 (m, 5H).

^{13}C NMR (101 MHz, $CDCl_3$) δ 169.22, 133.65, 131.72, 131.60, 128.45, 128.08, 127.87, 125.92, 125.35, 123.79, 57.91, 42.81, 38.03, 34.91, 32.83, 32.35, 27.04, 26.25, 22.50.

FTIR (cm^{-1}): 3309.1, 2908.0, 1643.8, 1538.0, 1448.1, 1353.2, 1129.9, 781.6;

HRMS (ESI) [M + H]$^+$ calculated for $C_{23}H_{28}NO$: 334.2165, found: 334.2163;

Compound 7d

(±)- (*N*-((1*R*,3*R*,5*R*,7*R*)-2-methyladamantan-2-yl)thiophene-2-carboxamide)

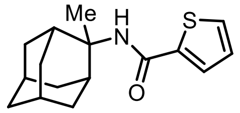

Following General Procedure B, the title compound was synthesized from 2-methylideneadamantane (**2c**) (75.0 mg, 0.50 mmol) and 2-thiophenecarbonitrile (109.2 mg, 1.0 mmol). The product was purified by silica gel flash column chromatography (PE: ethyl acetate = 50:1 to 10:1) to afford **7d** (78.5 mg, 57%) as a pale yellow solid.

^{1}H NMR (400 MHz, CDCl$_3$) δ 7.43 (dd, J = 3.7, 1.2 Hz, 1H), 7.40 (dd, J = 5.0, 1.2 Hz, 1H), 7.03 (dd, J = 5.0, 3.7 Hz, 1H), 5.85 (s, 1H), 2.27 (s, 2H), 2.07 – 1.95 (m, 4H), 1.83 (m, 2H), 1.74 – 1.58 (m, 9H).

^{13}C NMR (101 MHz, CDCl$_3$) δ 160.80, 140.71, 129.28, 127.52, 127.39, 59.14, 38.42, 35.54, 33.28, 27.45, 26.83, 23.16.

FTIR (cm^{-1}): 3338.0, 2915.8, 2857.0, 1628.0, 1539.3, 1506.6, 1300.8, 1155.3, 1130.6;

HRMS (ESI) [M + H]$^{+}$ calculated for $C_{16}H_{22}NOS$: 276.1417, found: 276.1417;

TLC: R_f = 0.85 (PE: ethyl acetate = 2:1).

Compound 7e

(±)-((3*R*,5*R*,7*R*)-*N*-((1*R*,3*R*,5*R*,7*R*)-2-methyladamantan-2-yl)adamantane-1-carboxamide)

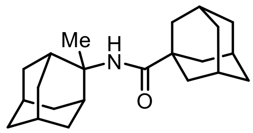

Following General Procedure C, the title compound was synthesized from 2-methylideneadamantane (**2c**) (75.0 mg, 0.50 mmol) and 1-adamantanecarbonitrile (403.2 mg, 2.5 mmol). The product was purified by silica gel flash column chromatography (PE: ethyl acetate = 50:1 to 10:1) to afford **7e** (83.6 mg, 51%) as a pale yellow solid.

^{1}H NMR (400 MHz, CDCl$_3$) δ 5.42 (s, 1H), 2.16 (s, 2H), 2.03 (s, 3H), 1.99 (m, 1H), 1.96 (m, 1H), 1.93 – 1.90 (m, 1H), 1.88 (m, 1H), 1.84 (m, 6H), 1.80 (p, J = 3.0 Hz, 1H), 1.69 (m, 9H), 1.65 – 1.56 (m, 3H), 1.47 (s, 3H), 1.28 – 1.20 (m, 1H).

^{13}C NMR (101 MHz, CDCl$_3$) δ 176.29, 57.25, 41.04, 39.33, 38.28, 36.38, 35.20, 33.09, 33.05, 28.08, 27.31, 26.69, 22.87.

FTIR (cm^{-1}): 3365.7, 2903.0, 2852.8, 1639.1, 1518.7, 1453.7, 1278.2 1180.9, 800.7;

HRMS (ESI) $[M + H]^+$ calculated for $C_{22}H_{34}NO$: 328.2635, found: 328.2634;

TLC: R_f = 0.90 (PE: ethyl acetate = 3:1).

Compound 7f (±)- (*N*-(5-methylundecan-5-yl)cyclopentanecarboxamide)

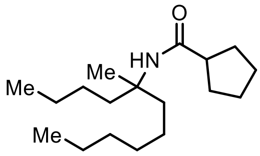

Following General Procedure C, the title compound was synthesized from 5-methyleneundecane (**2b**) (84.1 mg, 0.50 mmol) and cyanocyclopentane (238.0 mg, 2.5 mmol. The product was purified by silica gel flash column chromatography (PE: ethyl acetate = 50:1 to 10:1) to afford **7f** (87.0 mg, 62%) as a pale yellow solid.

^1H NMR (400 MHz, $CDCl_3$) δ 5.05 (s, 1H), 2.47 – 2.33 (m, 1H), 1.85 – 1.62 (m, 8H), 1.62 – 1.46 (m, 4H), 1.42 – 1.09 (m, 15H), 0.87 (t, J = 6.8 Hz, 6H).

^{13}C NMR (101 MHz, $CDCl_3$) δ 175.40, 56.06, 46.80, 38.55, 38.30, 32.00, 30.61, 29.80, 26.03, 24.64, 23.75, 23.20, 22.75, 14.27, 14.18.

FTIR (cm^{-1}): 3315.8, 2927.3, 2860.0, 1644.6, 1541.5, 1466.3, 1378.6, 1263.3, 1090.4;

HRMS (ESI) $[M + H]^+$ calculated for $C_{18}H_{36}NO$: 282.2791, found: 282.2787;

TLC: R_f = 0.50 (PE: ethyl acetate = 10:1).

Compound 7g (±)- (*N*-(5-methylundecan-5-yl)cyclopropanecarboxamide)

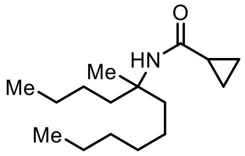

Following General Procedure C, the title compound was synthesized from 5-methyleneundecane (**2b**) (84.1 mg, 0.50 mmol) and cyclopropanecarbonitrile (167.8 mg, 2.5 mmol). The product was purified by silica gel flash column chromatography (PE: ethyl acetate = 50:1 to 10:1) to afford **7g** (88.8 mg, 70%) as a pale yellow solid.

^1H NMR (400 MHz, $CDCl_3$) δ 5.24 (s, 1H), 1.76 – 1.64 (m, 2H), 1.63 – 1.51 (m, 2H), 1.35 – 1.11 (m, 16H), 0.95 – 0.80 (m, 8H), 0.69 – 0.57 (m, 2H).

^{13}C NMR (101 MHz, $CDCl_3$) δ 172.11, 56.07, 38.39, 38.14, 31.68, 29.50, 25.73, 24.39, 23.44, 22.91, 22.46, 15.17, 13.98, 13.90, 6.34.

FTIR (cm^{-1}): 3312.0, 2954.8, 2927.1, 2858.9, 2358.8, 1643.6, 1545.1, 1466.2, 1271.9;

HRMS (ESI) $[M + H]^+$ calculated for $C_{16}H_{32}NO$: 254.2478, found: 254.2475;

TLC: R_f = 0.90 (PE: ethyl acetate = 1:1).

Compound 7h (±)- (*N*-(5-methylundecan-5-yl)isobutyramide)

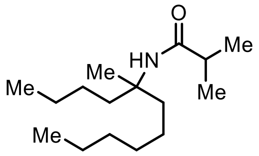

Following General Procedure C, the title compound was synthesized from 5-methyleneundecane (**2b**) (84.1 mg, 0.50 mmol) and isobutyronitrile (173.0 mg, 2.5 mmol). The product was purified by silica gel flash column chromatography (PE: ethyl acetate = 50:1 to 10:1) to afford **7h** (58.0 mg, 45%) as a pale yellow solid.

^1H NMR (400 MHz, $CDCl_3$) δ 5.02 (s, 1H), 2.23 (heptet, *J* = 6.9 Hz, 1H), 1.76 – 1.64 (m, 2H), 1.62 – 1.47 (m, 2H), 1.35 – 1.13 (m, 15H), 1.10 (d, *J* = 6.9 Hz, 6H), 0.87 (t, *J* = 6.8 Hz, 6H).

^{13}C NMR (101 MHz, $CDCl_3$) δ 176.07, 55.83, 38.40, 38.14, 36.49, 31.88, 29.68, 25.91, 24.49, 23.62, 23.08, 22.63, 19.84, 14.16, 14.07.

FTIR (cm^{-1}): 3315.2, 2956.8, 2926.4, 2858.9, 1646.4, 1541.5, 1466.6, 1379.9, 1094.6;

HRMS (ESI) $[M + H]^+$ calculated for $C_{16}H_{34}NO$: 256.2634, found: 256.2636;

TLC: R_f = 0.60 (PE: ethyl acetate = 10:1).

Compound 7i (±)- (*N*-(5-methylundecan-5-yl)benzamide)

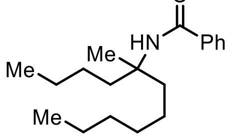

Following General Procedure C, the title compound was synthesized from 5-methyleneundecane (**2b**) (84.1 mg, 0.50 mmol) and benzonitrile (257.8 mg, 2.5 mmol). The product was purified by silica gel flash column chromatography (PE: ethyl acetate = 50:1 to 10:1) to afford **7i** (81.1 mg, 56%) as a pale yellow solid.

^{1}H NMR (400 MHz, $CDCl_3$) δ 7.78 – 7.65 (m, 2H), 7.54 – 7.32 (m, 3H), 5.78 (s, 1H), 1.86 (m, 2H), 1.71 (m, 2H), 1.39 – 1.17 (m, 15H), 0.87 (t, J = 6.8 Hz, 6H).

^{13}C NMR (101 MHz, $CDCl_3$) δ 165.72, 135.08, 129.97, 127.45, 125.64, 55.68, 37.50, 37.22, 30.85, 28.69, 24.98, 23.49, 22.72, 22.09, 21.64, 13.15, 13.07.

FTIR (cm^{-1}): 3311.5, 2954.9, 2927.1, 2858.4, 1639.9, 1534.2, 1488.9, 1466.1, 1292.3;

HRMS (ESI) $[M + H]^+$ calculated for $C_{19}H_{32}NO$: 290.2478, found: 290.2474;

TLC: R_f = 0.60 (PE: ethyl acetate = 10:1).

Compound 7j (±)- (2-(4-methoxyphenyl)-*N*-(5-methylundecan-5-yl)acetamide)

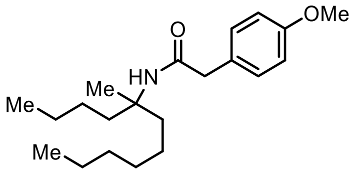

Following General Procedure C, the title compound was synthesized from 5-methyleneundecane (**2b**) (84.1 mg, 0.50 mmol) and 4-methoxybenzyl cyanide (368.0 mg, 2.5 mmol). The product was purified by silica gel flash column chromatography (PE: ethyl acetate = 50:1 to 10:1) to afford **7j** (68.4 mg, 41%) as a pale yellow solid.

^{1}H NMR (400 MHz, $CDCl_3$) δ 7.19 – 7.04 (m, 2H), 6.91 – 6.83 (m, 2H), 4.99 (s, 1H), 3.79 (s, 3H), 3.41 (s, 2H), 1.61 (m, 2H), 1.55 – 1.41 (m, 2H), 1.29 – 1.14 (m, 11H), 1.11 – 1.00 (m, 4H), 0.87 (t, J = 6.8 Hz, 6H).

^{13}C NMR (101 MHz, $CDCl_3$) δ 170.19, 158.37, 130.00, 127.21, 113.96, 55.86, 54.87, 43.71, 37.94, 37.65, 31.46, 29.22, 25.43, 23.80, 23.13, 22.63, 22.21, 13.73, 13.6

FTIR (cm^{-1}): 3308.1, 2928.1, 2858.6, 1646.00, 1511.0, 1246.7 1176.9, 1037.4, 802.4;

HRMS (ESI) $[M + H]^+$ calculated for $C_{21}H_{36}NO$: 334.2741, found: 334.2741;

TLC: R_f = 0.25 (PE: ethyl acetate = 10:1).

III. Gram-scale experiment

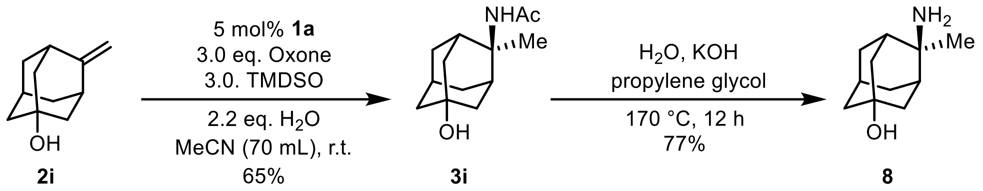

To a 250 mL oven-dried round bottom flask containing a magnetic stir bar was added **2i** (1.15 g, 7.00 mmol, 1.00 equiv.), Co catalyst **1a** (212 mg, 0.350 mmol, 0.0500 equiv.) and oxone (100 mesh, 12.9 g, 21.0 mmol, 3.00 equiv.). The reaction vessel was then briefly evacuated and backfilled with nitrogen (this sequence was repeated a total of three times). Anhydrous acetonitrile (70 mL), H_2O (280 μL, 15.4 mmol, 2.20 equiv.) and 1,1,3,3-tetramethyldisiloxane (3.80 mL, 21.0 mmol, 3.00 equiv.) were added to the reaction vessel via syringe sequentially. The reaction mixture was stirred at r.t. for 21 h. The resulting mixture was filtered and dried over Na_2SO_4, concentrated *in vacuo* and purified by silica gel chromatography to afford **3i** (major diastereomer, 1.02 g, 65%) as a yellow solid.

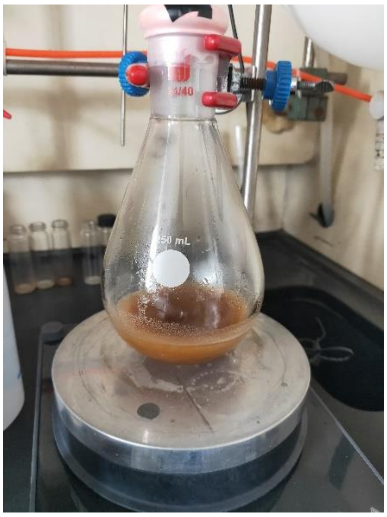

Supplementary Figure 2. Reaction mixture of the gram-scale experiment.

A mixture of potassium hydroxide (powder, 0.34 g, 6.0 mmol), water (33 μL), and propylene glycol (0.25 mL) was stirred at room temperature for 1 h, to which was added **3i** (0.12 g, 0.50 mol). The mixture was stirred at 170 °C for 12 h, cooled to r.t. followed by the addition of ice-cold water (10 mL). The reaction mixture was extracted with dichloromethane. The combined organic layers were dried over Na_2SO_4, filtered and concentrated *in vacuo* to afford **8** (69.8 mg, 77% yield) as a pale yellow solid.

^{1}H NMR (400 MHz, $CDCl_3$) δ 2.12 – 2.06 (m, 3H), 1.88 (m, 2H), 1.80 (s, 3H), 1.73 (s,

2H), 1.66 (s, 2H), 1.54 – 1.49 (m, 3H), 1.27 – 1.23 (m, 1H), 1.21 (s, 3H).

^{13}C NMR (101 MHz, $CDCl_3$) δ 67.62, 52.06, 46.26, 41.64, 40.65, 33.05, 29.52, 27.01.

FTIR (cm^{-1}): 3539.4, 2920.8, 2851.9, 1659.1, 1114.4, 832.0;

HRMS (ESI) $[M + H]^+$ calculated for $C_{11}H_{20}NO$: 182.1539, found: 182.1539;

TLC: R_f = 0.25 ($CHCl_3$: CH_3OH: acetone: $NH_3{\bullet}H_2O$ = 9:1.5:1:0.014, ninhydrin in *n*-butyl alcohol as a visualizing agent)

IV. Mechanistic Investigation

IV-A. With Deuterium-Labeled Silane (Figure 5a)

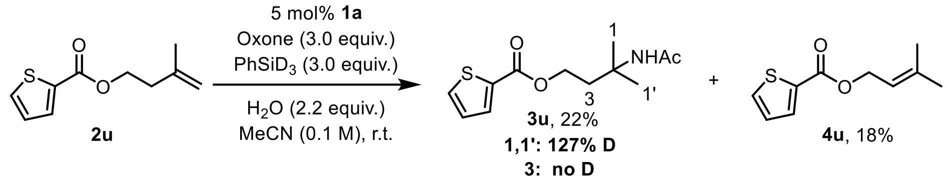

An oven-dried 10 mL re-sealable screw-cap vial equipped with a Teflon-coated magnetic stir bar was charged with Co catalyst **1a** (3.0 mg, 0.0050 mmol, 0.050 equiv.) and oxone (100 mesh, 184 mg, 0.30 mmol, 3.0 equiv.). The reaction vessel was then briefly evacuated and backfilled with nitrogen (this sequence was repeated a total of three times). Anhydrous acetonitrile (1.0 mL), **2u** (19.6 mg, 0.10 mmol, 1.0 equiv.), degassed H_2O (4 μL, 0.22 mmol, 2.2 equiv.) and $PhSiD_3$ (33 mg, 0.30 mmol, 3.0 equiv.) were added to the reaction vessel via syringe sequentially. The reaction mixture was stirred at r.t. for 18 h before an NMR internal standard (phenanthrene, 0.10 mmol, 18 mg) was added. The mixture was filtered through a short pad of silica gel with another 5 mL CH_2Cl_2/MeOH (20/1) as an eluent. The solvents were removed *in vacuo* and the residue was analyzed by ^{1}H NMR spectroscopy.

The levels of deuteration in each position (1, 1' and 3) were measured by ^{1}H NMR spectroscopy. **3u** with multiple deuterium substitution at the 1,1' position was noticed by ^{1}H and ^{13}C NMR, as well as GCMS analysis. The ^{1}H NMR spectrum of **3u** showed that 127% deuterium incorporation at C1 and no deuterium incorporation at C3.

IV-B. Test for Proton-Activation (Figure 5b)

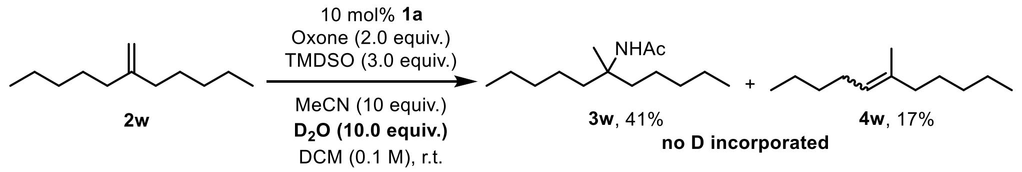

An oven-dried 10 mL re-sealable screw-cap vial equipped with a Teflon-coated magnetic stir bar was charged with Co catalyst **1a** (6.0 mg, 0.010 mmol, 0.010 equiv.) and oxone (100 mesh, 123 mg, 0.20 mmol, 2.0 equiv.). The reaction vessel was then briefly evacuated and backfilled with nitrogen (this sequence was repeated a total of three times). Anhydrous CH_2Cl_2 (1.0 mL), **2w** (17 mg, 0.10 mmol, 1.0 equiv.), degassed D_2O (20 μL, 1.0 mmol, 10 equiv.) and MeCN (54 μL, 1.0 mmol, 10 equiv.) were added to the reaction vessel via syringe sequentially. The reaction mixture was stirred at r.t. for 18 h before an NMR internal standard (phenanthrene, 0.10 mmol, 18 mg) was added. The mixture was filtered through a short pad of silica gel with another 5 mL CH_2Cl_2/MeOH (20/1) as an

eluent. The solvents were removed *in vacuo* and the residue was analyzed by ^{1}H NMR spectroscopy to obtain the yields. Purification by silica gel chromatography afforded **3w** as a colorless oil. **4w** (ca. 1:1 mixture of E/Z isomers) could not be separated from the hydrogenation byproduct. Characteristic ^{1}H NMR signals of **4w**: 1.57 (s, 3H), 1.66 (d, *J* = 1.3 Hz, 3H). A standard sample of **3w** was independently prepared following General Procedure A with slight modification, where **2w** (0.10 mmol) was used. Deuterium incorporation in **3w** and **4w** was excluded by comparing their ^{1}H NMR spectra and GCMS data with standard samples prepared under D-free conditions.

***N*-(6-methylundecan-6-yl)acetamide (3w):** ^{1}H NMR (400 MHz, $CDCl_3$) δ 5.09 (s, 1H), 1.90 (s, 3H), 1.76 – 1.63 (m, 2H), 1.56 (dt, *J* = 13.8, 6.9 Hz, 2H), 1.23 (m, 15H), 0.87 (t, *J* = 6.9 Hz, 6H).

^{13}C NMR (101 MHz, $CDCl_3$) δ 169.09, 56.16, 38.10, 32.01, 24.30, 23.16, 22.49, 13.86.

FTIR (cm^{-1}): 3298.7, 2924.9, 2857.9, 1647.6, 1553.0, 1465.7, 1370.6, 1302.3;

HRMS (ESI) $[M + H]^+$ calculated for $C_{14}H_{30}NO$: 228.2322, found: 228.2322;

TLC: R_f = 0.90 (PE: ethyl acetate = 1:1).

IV-C. Test for reversibility of HAT(Figure 5c)

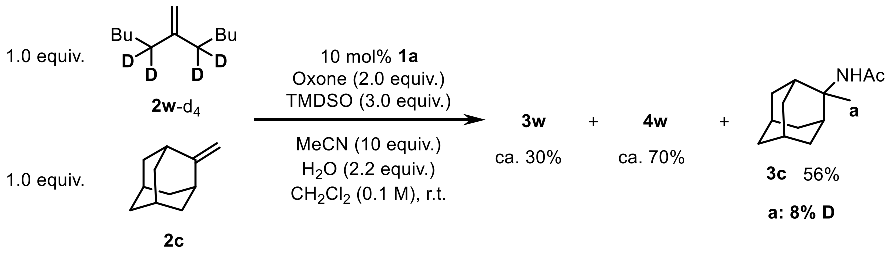

An oven-dried 10 mL re-sealable screw-cap vial equipped with a Teflon-coated magnetic stir bar was charged with Co catalyst **1a** (18 mg, 0.030 mmol, 0.010 equiv.) and oxone (369 mg, 0.60 mmol, 2.0 equiv.). The reaction vessel was then briefly evacuated and backfilled with nitrogen (this sequence was repeated a total of three times). Anhydrous CH_2Cl_2 (1.0 mL), **2w**-d_4 (52 mg, 0.30 mmol, 1.0 equiv.), **2c** (45 mg, 0.30 mmol, 1.0 equiv.), degassed H_2O (12 μL, 0.66 mmol, 2.2 equiv.) and MeCN (162 μL, 3.0 mmol, 10 equiv.) were added to the reaction vessel via syringe sequentially. The reaction mixture was stirred at r.t. for 18 h before an NMR internal standard (phenanthrene, 0.10 mmol, 18 mg) was added. The mixture was filtered through a short pad of silica gel with another 5 mL CH_2Cl_2/MeOH (20/1) as an eluent. The solvents were removed *in vacuo* and the residue was analyzed by ^{1}H NMR spectroscopy to obtain the yields. The product was purified by silica gel flash column chromatography to afford **3c**.

The level of deuteration at C(a) were measured by ^{1}H NMR spectroscopy to be 8% and confirmed by GCMS analysis.

IV-D. Effect of the amount of nitrile (Figure 5d)

An oven-dried 10 mL re-sealable screw-cap vial equipped with a Teflon-coated magnetic stir bar was charged with Co catalyst **1a** (6.0 mg, 0.010 mmol, 0.10 equiv.) and oxone (100 mesh, 123 mg, 0.20 mmol, 2.0 equiv.). The reaction vessel was then briefly evacuated and backfilled with nitrogen (this sequence was repeated a total of three times). Anhydrous dichloromethane (1.0 mL), **2w** (16.8 mg, 0.10 mmol, 1.0 equiv.), degassed H_2O (4 μL, 0.22 mmol, 2.2 equiv.), 1,1,3,3-tetramethyldisiloxane (55 μL, 0.30 mmol, 3.0 equiv.) and cyclopropanecarbonitrile (X equiv., X = 1, 2, 3, 4 or 5) were added to the reaction vessel via syringe sequentially. The reaction mixture was stirred at r.t. for 18 h before an NMR internal standard (phenanthrene) was added. The mixture was filtered through a short pad of silica gel with another 5 mL CH_2Cl_2/MeOH (20/1) as an eluent. The solvents were removed *in vacuo* and the residue was analyzed by ^{1}H NMR spectroscopy. Characteristic ^{1}H NMR signals: 0.64 – 0.56 (m, 2H).

***N*-(6-methylundecan-6-yl)cyclopropanecarboxamide (3w')**: ^{1}H NMR (400 MHz, $CDCl_3$) δ 5.33 (s, 1H), 1.73 – 1.67 (m, 2H), 1.60 – 1.55 (m, 2H), 1.31 – 1.17 (m, 16H), 0.87 – 0.82 (m, 8H), 0.64 – 0.56 (m, 2H).

^{13}C NMR (101 MHz, $CDCl_3$) δ 171.99, 55.93, 38.18, 31.88, 24.22, 22.98, 22.32, 14.95, 13.70, 6.14.

FTIR (cm^{-1}): 2930.7, 1656.5, 1511.5, 1466.8, 1258.3, 1032.4, 921.5, 801.2, 736.3;

TLC: R_f = 0.70 (PE: ethyl acetate = 3:1).

IV-E. Tests of Alternative Nucleophiles (Figure 5e)

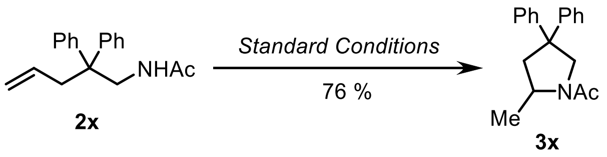

An oven-dried 10 mL re-sealable screw-cap vial equipped with a Teflon-coated magnetic stir bar was charged with Co catalyst **1a** (6.0 mg, 0.010 mmol, 0.10 equiv.) and oxone (100 mesh, 123 mg, 0.20 mmol, 2.0 equiv.). The reaction vessel was then briefly evacuated and backfilled with nitrogen (this sequence was repeated a total of three times). Anhydrous acetonitrile (1.0 mL), **2x** (28 mg, 0.10 mmol, 1.0 equiv.), degassed H_2O (2 μL, 0.11 mmol, 1.1 equiv.) and 1,1,3,3-tetramethyldisiloxane (55 μL, 0.30 mmol, 3.0 equiv.) were added to the reaction vessel via syringe sequentially. The reaction mixture was stirred at r.t. for 18 h before an NMR internal standard (phenanthrene) was added. The mixture was filtered

through a short pad of silica gel with another 5 mL CH_2Cl_2/MeOH (20/1) as an eluent. The solvents were removed *in vacuo* and the residue was analyzed by ^{1}H NMR spectroscopy. **3x** was detected in 76% yield from ^{1}H NMR spectroscopy matching literature reports[24].

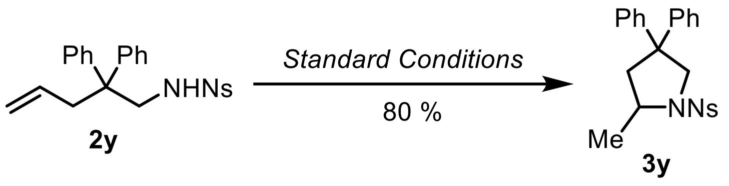

An oven-dried 10 mL re-sealable screw-cap vial equipped with a Teflon-coated magnetic stir bar was charged with Co catalyst **1a** (6.0 mg, 0.010 mmol, 0.10 equiv.) and oxone (100 mesh, 123 mg, 0.20 mmol, 2.0 equiv.). The reaction vessel was then briefly evacuated and backfilled with nitrogen (this sequence was repeated a total of three times). Anhydrous acetonitrile (1.0 mL), **2y** (42.2 mg, 0.10 mmol, 1.0 equiv.), degassed H_2O (2 μL, 0.11 mmol, 1.1 equiv.) and 1,1,3,3-tetramethyldisiloxane (55 μL, 0.30 mmol, 3.0 equiv.) were added to the reaction vessel via syringe sequentially. The reaction mixture was stirred at r.t. for 18 h before an NMR internal standard (phenanthrene) was added. The mixture was filtered through a short pad of silica gel with another 5 mL CH_2Cl_2/MeOH (20/1) as an eluent. The solvents were removed *in vacuo* and the residue was analyzed by ^{1}H NMR spectroscopy. **3y** was detected in 80% yield from ^{1}H NMR spectroscopy matching literature reports[24].

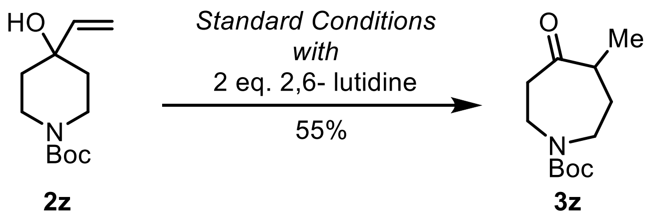

An oven-dried 10 mL re-sealable screw-cap vial equipped with a Teflon-coated magnetic stir bar was charged with Co catalyst **1a** (6.0 mg, 0.010 mmol, 0.10 equiv.) and oxone (100 mesh, 123 mg, 0.20 mmol, 2.0 equiv.). The reaction vessel was then briefly evacuated and backfilled with nitrogen (this sequence was repeated a total of three times). Anhydrous acetonitrile (1.0 mL), **2z** (22.7 mg, 0.10 mmol, 1.0 equiv.), degassed H_2O (2 μL, 0.11 mmol, 1.1 equiv.), 1,1,3,3-tetramethyldisiloxane (55 μL, 0.30 mmol, 3.0 equiv.) and 2,6-lutidine (21.4 mg, 0.20 mmol, 2.0 equiv.) were added to the reaction vessel via syringe sequentially. The reaction mixture was stirred at r.t. for 18 h before an NMR internal

standard (phenanthrene) was added. The mixture was filtered through a short pad of silica gel with another 5 mL CH_2Cl_2/MeOH (20/1) as an eluent. The solvents were removed *in vacuo* and the residue was analyzed by ^{1}H NMR spectroscopy. **3z** was detected in 55% yield from ^{1}H NMR spectroscopy matching literature reports[25].

IV-F. Effect of the amount of 2,6-lutidine

An oven-dried 10 mL re-sealable screw-cap vial equipped with a Teflon-coated magnetic stir bar was charged with Co catalyst **1a** (6.0 mg, 0.010 mmol, 0.10 equiv.) and oxone (100 mesh, 123 mg, 0.20 mmol, 2.0 equiv.). The reaction vessel was then briefly evacuated and backfilled with nitrogen (this sequence was repeated a total of three times). Anhydrous acetonitrile (1.0 mL), **2a** (18.1 mg, 0.10 mmol, 1.0 equiv.), degassed H_2O (2 μL, 0.11 mmol, 1.1 equiv.), 1,1,3,3-tetramethyldisiloxane (73 μL, 0.40 mmol, 4.0 equiv.) and 2,6-lutidine were added to the reaction vessel via syringe sequentially. The reaction mixture was stirred at r.t. for 18 h before an NMR internal standard (phenanthrene) was added. The mixture was filtered through a short pad of silica gel with another 5 mL CH_2Cl_2/MeOH (20/1) as an eluent. The solvents were removed *in vacuo* and the residue was analyzed by ^{1}H NMR spectroscopy.

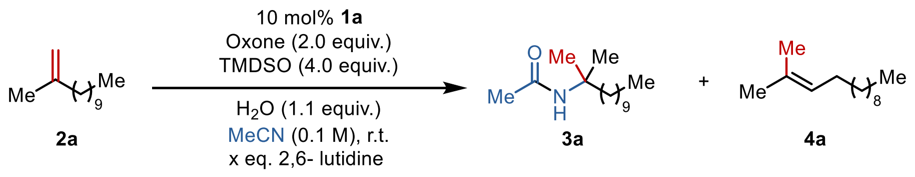

Entry	x	Conv. (%)	**3a** (%)	**4a** (%)
1	5	100	82	4
2	10	88	54	16

Supplementary Figure 3. Effect of the Amount of 2,6- lutidine.

IV-G. Effect of the H_2O

An oven-dried 10 mL re-sealable screw-cap vial equipped with a Teflon-coated magnetic stir bar was charged with Co catalyst **1a** (6.0 mg, 0.010 mmol, 0.10 equiv.) and oxone (100 mesh, 123 mg, 0.20 mmol, 2.0 equiv.). The reaction vessel was then briefly evacuated and backfilled with nitrogen (this sequence was repeated a total of three times). Anhydrous acetonitrile (1.0 mL), **2a** (18.1 mg, 0.10 mmol, 1.0 equiv.), degassed H_2O and 1,1,3,3-tetramethyldisiloxane (73 μL, 0.40 mmol, 4.0 equiv.) were added to the reaction vessel via syringe sequentially. The reaction mixture was stirred at r.t. for 18 h before an NMR internal standard (phenanthrene) was added. The mixture was filtered through a

short pad of silica gel with another 5 mL CH_2Cl_2/MeOH (20/1) as an eluent. The solvents were removed *in vacuo* and the residue was analyzed by ^1H NMR spectroscopy.

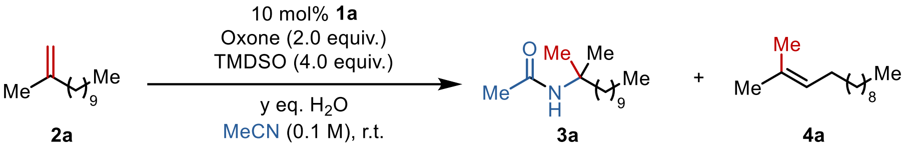

Entry	y	Conv. (%)	**3a** (%)	**4a** (%)	Hydration (%)
1	5	100	96	4	0
2	20	76	55	0	0
3	100	70	0	5	12

Supplementary Figure 4. Effect of the H_2O.

IV-H. HRMS analysis of possible intermediates

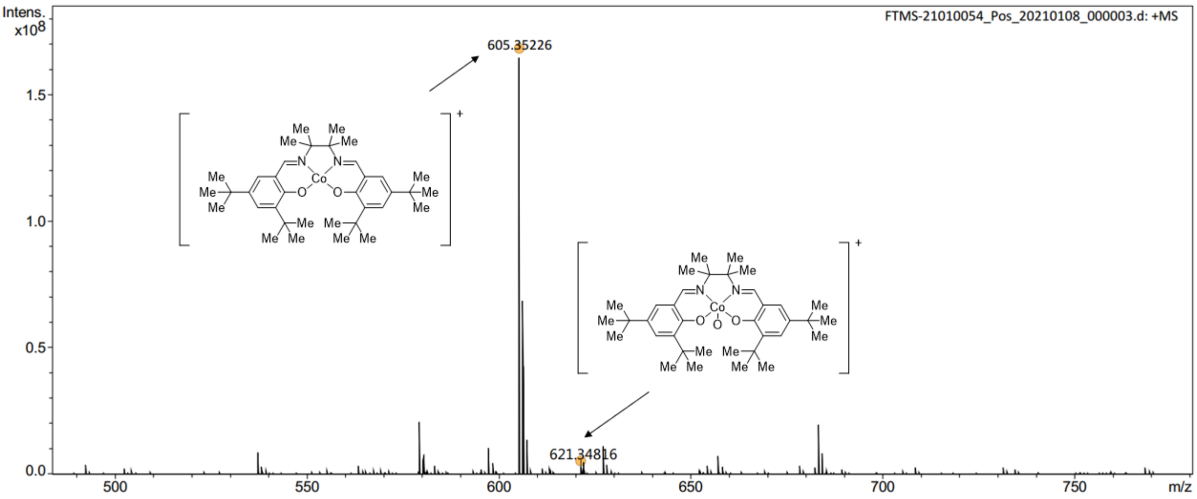

Supplementary Figure 5. HRMS spectrum of a reaction mixture.

IV-I. Radical clock experiment

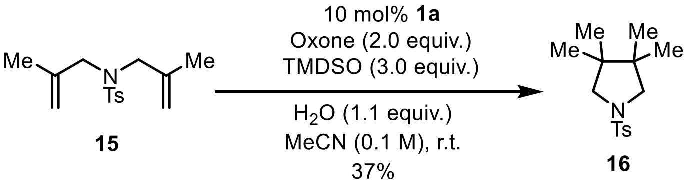

An oven-dried 10 mL re-sealable screw-cap vial equipped with a Teflon-coated magnetic stir bar was charged with Co catalyst **1a** (6.0 mg, 0.010 mmol, 0.10 equiv.) and oxone (100 mesh, 123 mg, 0.20 mmol, 2.0 equiv.). The reaction vessel was then briefly evacuated and backfilled with nitrogen (this sequence was repeated a total of three times). Anhydrous

acetonitrile (1.0 mL), **15** (28 mg, 0.10 mmol, 1.0 equiv.), degassed H_2O (2 μL, 0.11 mmol, 1.1 equiv.) and 1,1,3,3-tetramethyldisiloxane (55 μL, 0.30 mmol, 3.0 equiv.) were added to the reaction vessel via syringe sequentially. The reaction mixture was stirred at r.t. for 18 h before an NMR internal standard (phenanthrene) was added. The mixture was filtered through a short pad of silica gel with another 5 mL CH_2Cl_2/MeOH (20/1) as an eluent. The solvents were removed *in vacuo* and the residue was analyzed by ^{1}H NMR spectroscopy.

Compound **16** was detected from crude ^{1}H NMR.[26] Characteristic ^{1}H NMR signals: 3.12 (s, 4H), 0.73 (s, 12H).

V. X-Ray Crystallography Analysis

Crystallographic Data of Compound 3h

Compound **3h** was dissolved in diethyl ether. The solvent was slowly evaporated at ambient temperature to afford the single crystal.

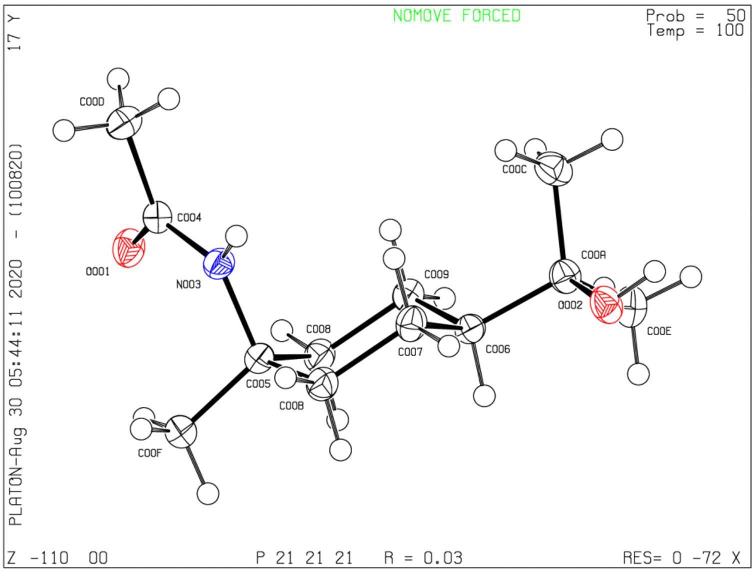

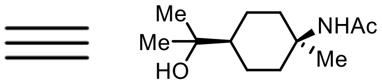

3h
CCDC 2026144

Empirical formula	$C_{12}H_{23}NO_2$
Formula weight	213.32
Temperature/K	100
Space group	P212121
Hall group	P2ac2ab
a/Å	8.0627(1)
b/Å	8.2234(1)
c/Å	19.1383(2)
α/°	90
β/°	90
γ/°	90
Volume/Å^3	1268.92(3)
Z	4

ϱ g/cm3	1.117
μ/mm-1	0.591
F(000)	472.0
Radiation	CuKα (λ = 1.54184)
h,k,l_{max}	10,10,24
N_{ref}	2628[1538]
T_{min},T_{max}	0.932,0.971

Crystallographic Data of Compound 3i

Compound **3i** was dissolved in diethyl ether. The solvent was slowly evaporated at ambient temperature to afford the single crystal.

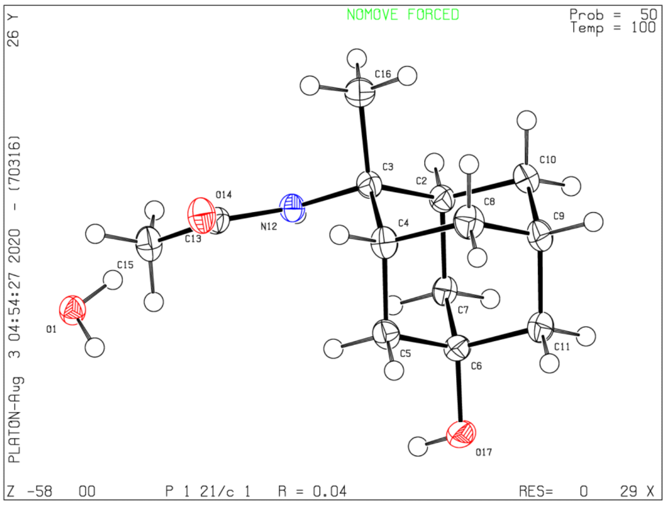

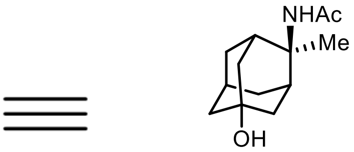

3i

CCDC 2025806

Empirical formula	$C_{13}H_{21}NO_2$
Formula weight	223.32
Temperature/K	100
Space group	P21/c
Hall group	-P2ybc
a/Å	12.8834(1)

b/Å	7.9504(1)
c/Å	13.1471(1)
α/°	90
β/°	94.302(1)
γ/°	90
Volume/Å^3	1342.84(2)
Z	4
ϱ g/cm3	1.194
μ/mm-1	0.676
F(000)	528.0
Radiation	CuKα (λ = 1.54184)
h,k,l_{max}	16,9,16
N_{ref}	2780
T_{min},T_{max}	0.922,0.987

VI. Supplementary References

1. Shigehisa, H.; Ano, T.; Honma, H.; Ebisawa, K.; Hiroya, K., Co-Catalyzed Hydroarylation of Unactivated Olefins. *Org. Lett.* **2016,** *18*, 3622-3625.

2. Ren, W.; Chu, J.; Sun, F.; Shi, Y., Pd-Catalyzed Highly Chemo- and Regioselective Hydrocarboxylation of Terminal Alkyl Olefins with Formic Acid. *Org. Lett.* **2019,** *21*, 5967-5970.

3. Weinstabl, H.; Gaich, T.; Mulzer, J., Application of the RodriguezPattenden Photo-Ring Contraction: Total Synthesis and Configurational Reassignment of 11-Gorgiacerol and 11-Epigorgiacerol. *Org. Lett.* **2012,** *14*, 2834–2837.

4. Schevenels, F. T.; Shen, M.; Snyder, S. A., Isolable and Readily Handled Halophosphonium Pre-reagents for Hydro- and Deuteriohalogenation. *J. Am. Chem. Soc.* **2017,** *139*, 6329-6337.

5. Gaspar, B.; Carreira, E. M., Cobalt Catalyzed Functionalization of Unactivated Alkenes: Regioselective Reductive C-C Bond Forming Reactions. *J. Am. Chem. Soc.* **2009,** *131*, 13214–13215.

6. Wang, D.; Xue, X. S.; Houk, K. N.; Shi, Z., Mild Ring-Opening 1,3-Hydroborations of Non-Activated Cyclopropanes. *Angew. Chem. Int. Ed.* **2018,** *57*, 16861-16865.

7. Zhou, J.; Fu, G. C., Palladium-Catalyzed Negishi Cross-Coupling Reactions of Unactivated Alkyl Iodides, Bromides, Chlorides, and Tosylates. *J. Am. Chem. Soc.* **2003,** *125*, 12527-12530.

8. Nifant'ev, I. E.; Minyaev, M. E.; Tavtorkin, A. N.; Vinogradov, A. A.; Ivchenko, P. V., Branched alkylphosphinic and disubstituted phosphinic and phosphonic acids: effective synthesis based on α-olefin dimers and applications in lanthanide extraction and separation. *RSC Adv.* **2017,** *7*, 24122-24128.

9. Spangler, B.; Fontaine, S. D.; Shi, Y.; Sambucetti, L.; Mattis, A. N.; Hann, B.; Wells, J. A.; Renslo, A. R., A Novel Tumor-Activated Prodrug Strategy Targeting Ferrous Iron Is Effective in Multiple Preclinical Cancer Models. *J. Med. Chem.* **2016,** *59*, 11161-11170.

10. Burgess, K.; Donk, W. A.; Jarstfer, M. B.; Ohlmeyer, M. J., Further Evidence for the Role of dπ-pπ Bonding in Rhodium-Mediated Hydroborations. *J. Am. Chem. Soc.* **1991,** *113*, 6139-6144.

11. Fontan, N.; Alvarez, R.; de Lera, A. R., Stereoselective synthesis by olefin metathesis and characterization of eta-carotene (7,8,7',8'-tetrahydro-beta,beta-carotene). *J. Nat. Prod.* **2012,** *75*, 975-979.

12. Frontier, A. J.; Danishefsky, S. J.; Koppel, G. A.; Meng, D., A useful α, α' - annulation reaction of enamines. *Tetrahedron* **1998,** *54*, 12721-12736.

13. Green, S. A.; Vasquez-Cespedes, S.; Shenvi, R. A., Iron-Nickel Dual-Catalysis: A New Engine for Olefin Functionalization and the Formation of Quaternary Centers. *J. Am. Chem. Soc.* **2018,** *140*, 11317-11324.

14. Olah, G. A., Reddy, V. P., Prakash, G. K. S., Peterson (Silyl-Wittig) Methylenation of Carbonyl Compounds Using Nafion-H Catalyzed Hydroxy-Trimethylsilane Elimination of *β*-Hydroxysilanes. *Synthesis* **1991**, *1*, 29-30.

15. Gao, Y.; Cao, Z.; Zhang, Q.; Guo, R.; Ding, F.; You, Q.; Bi, J.; Zhang, Y., Total Synthesis of the Proposed Structure of Penasulfate A: l-Arabinose as a Source of Chirality. *J. Nat. Prod.* **2019,** *82*, 1908-1916.

16. Warner, R. M.; Leitch, L. C., Deuterated Organic Compounds XXV Synthesis of certain Deuterated n-Undecanes and n-Dodecanes *Journal of Labelled Compounds* **1965,** *1*, 42-53.

17. Kitano, Y.; Okada, I.; Chiba, K., Facile Synthesis of N-Substituted Amides from Alcohols and Amides. *Synthesis* **2013,** *45*, 1069-1075.

18. Kolocouris, A.; Koch, A.; Kleinpeter, E.; Stylianakis, I., 2-Substituted and 2,2-disubstituted

adamantane derivatives as models for studying substituent chemical shifts and C–Hax···Yax cyclohexane contacts—results from experimental and theoretical NMR spectroscopic chemical shifts and DFT structures. *Tetrahedron* **2015,** *71*, 2463-2481.
19. Kalkhambkar, R. G.; Waters, S. N.; Laali, K. K., Highly efficient synthesis of amides via Ritter chemistry with ionic liquids. *Tetrahedron Lett* **2011,** *52*, 867-871.
20. Kiyokawa, K.; Watanabe, T.; Fra, L.; Kojima, T.; Minakata, S., Hypervalent Iodine(III)-Mediated Decarboxylative Ritter-Type Amination Leading to the Production of alpha-Tertiary Amine Derivatives. *J. Org. Chem.* **2017,** *82*, 11711-11720.
21. For an analogous structure:Crossley, S. W. M., Barabe, F., Shenvi, R. A., Simple, Chemoselective, Catalytic Olefin Isomerization. *J. Am. Chem. Soc.* **2014**, *136*, 16788-16791.
22. Michaudel, Q.; Thevenet, D.; Baran, P. S., Intermolecular Ritter-Type C-H Amination of Unactivated sp(3) Carbons. *J. Am. Chem. Soc.* **2012**, *134*, 2547-2550.
23. For an analogous structure: (1) Electroorganic Chemistry; 145: Coupling Reaction of an Olefin with a Radical NO_3 ·Generated by Anodic Oxidation of NO_3 Shono, T.; Chuyankamnerdkarn, M.; Maekawa, H.; Ishifune, M.; Kashimura, S. *Synthesis.* **1994**, *9*, 895-897. (2) Protonated form: Remote functionalization of (–)-menthol—synthesis of 4a,5,6,7,8,8a-hexahydro-4H-benzo[1,3]oxazine derivatives with the Selectfluor^{TM} reagent F–TEDA–BF_4. Banks, R. E.; Lawrence, N. J.; Besheesh, M. K.; Popplewell, A. L.; Pritchard, R. G. *Chem. Commun.*, **1996**, *1629*-1630.
24. Shigehisa, H.; Koseki, N.; Shimizu, N.; Fujisawa, M.; Niitsu, M.; Hiroya, K., Catalytic Hydroamination of Unactivated Olefins Using a Co Catalyst for Complex Molecule Synthesis. *J. Am. Chem. Soc.* **2014,** *136*, 13534-13537.
25. Touney, E. E.; Foy, N. J.; Pronin, S. V. Catalytic radical−polar crossover reactions of allylic alcohols. *J. Am. Chem. Soc.* **2018**, *140*, 16982−16987.
26. Zhang, J.; Yang, C.-G.; He, C., Gold(I)-Catalyzed Intra- and Intermolecular Hydroamination of Unactivated Olefins. *J. Am. Chem. Soc.* **2006,** *128*, 1798-1799.

VII. NMR Spectra

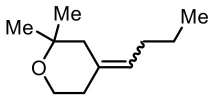

2d

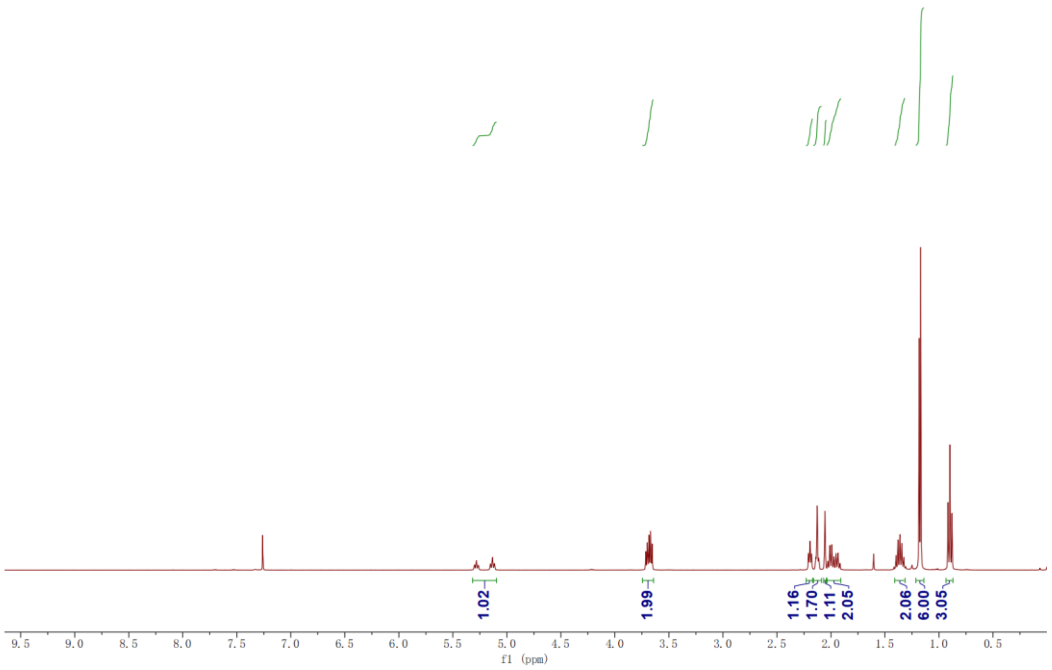

Supplementary Figure 6. ^{1}H NMR spectrum of 2d

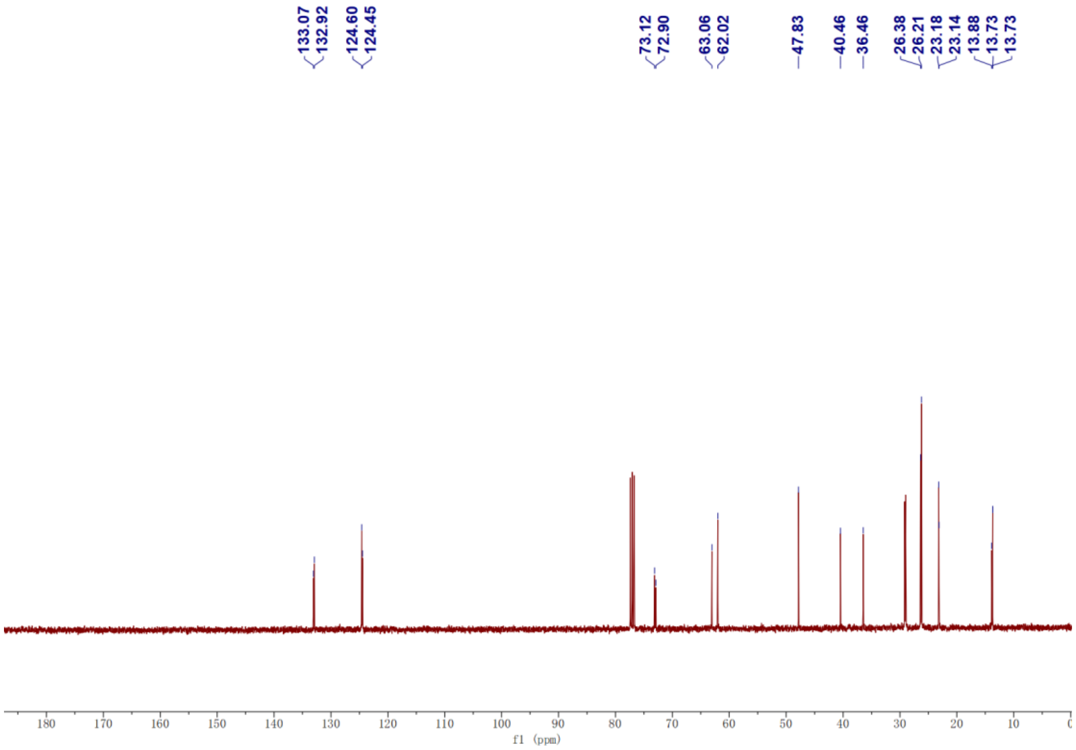

Supplementary Figure 7. ^{13}C NMR spectrum of 2d

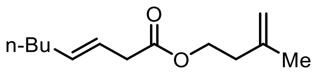

2l

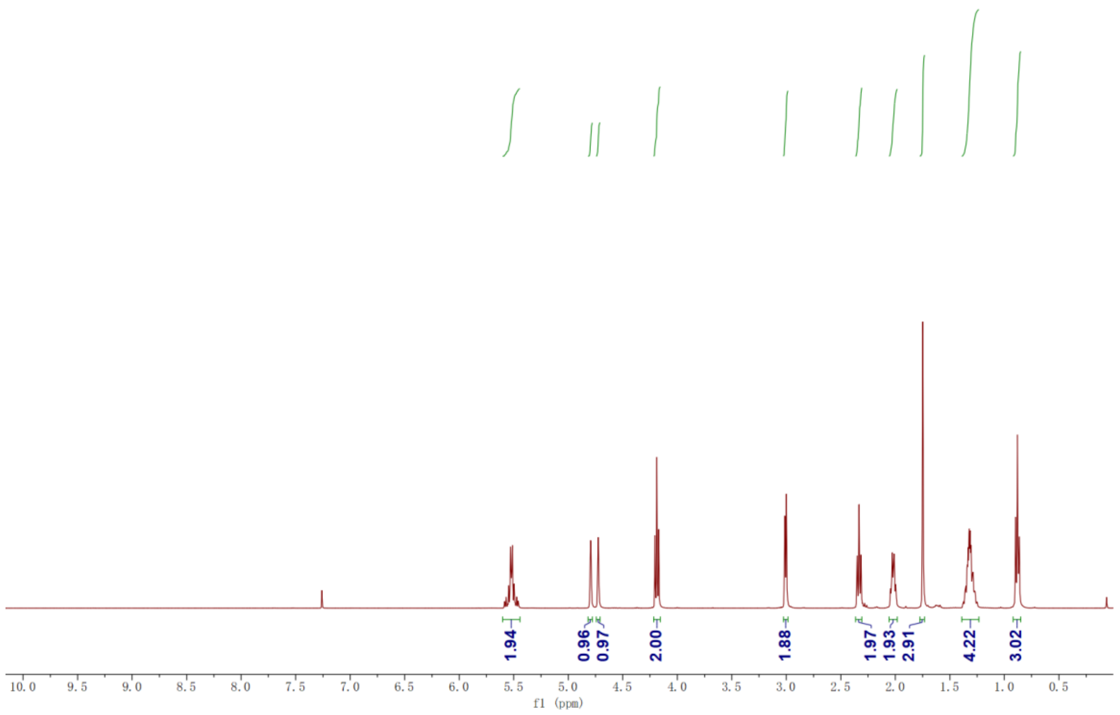

Supplementary Figure 8. ¹H NMR spectrum of 2l

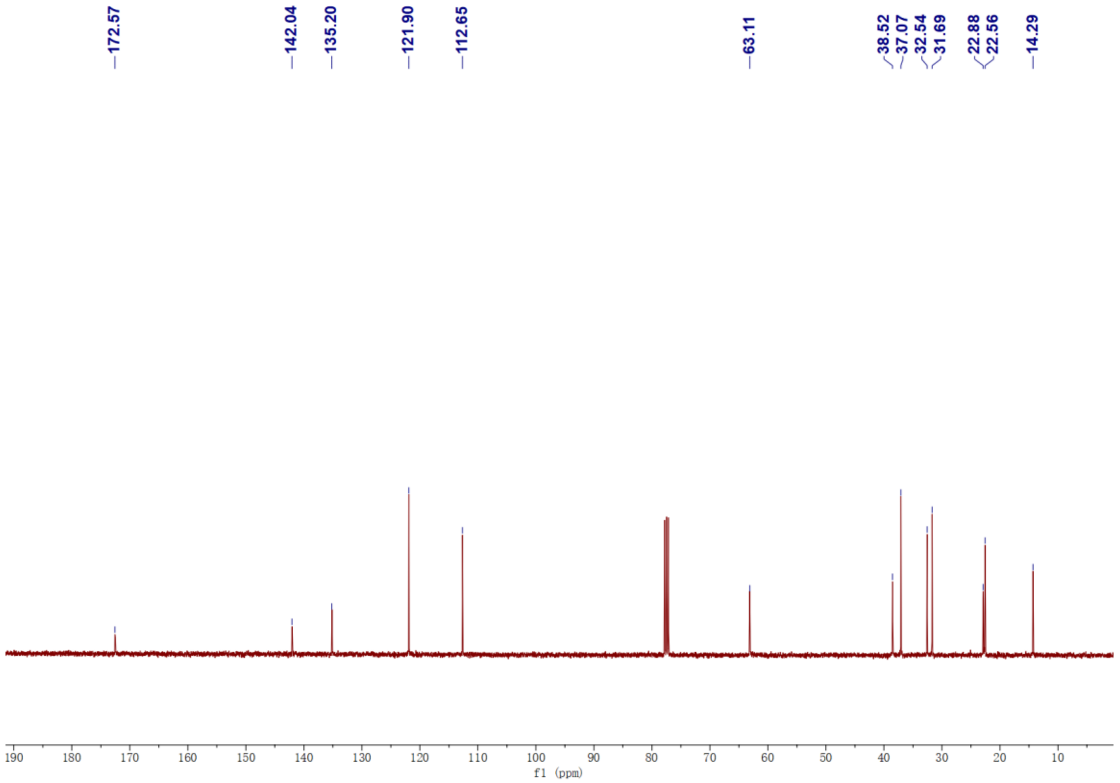

Supplementary Figure 9. ¹³C NMR spectrum of 2l

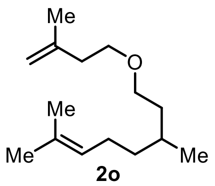

2o

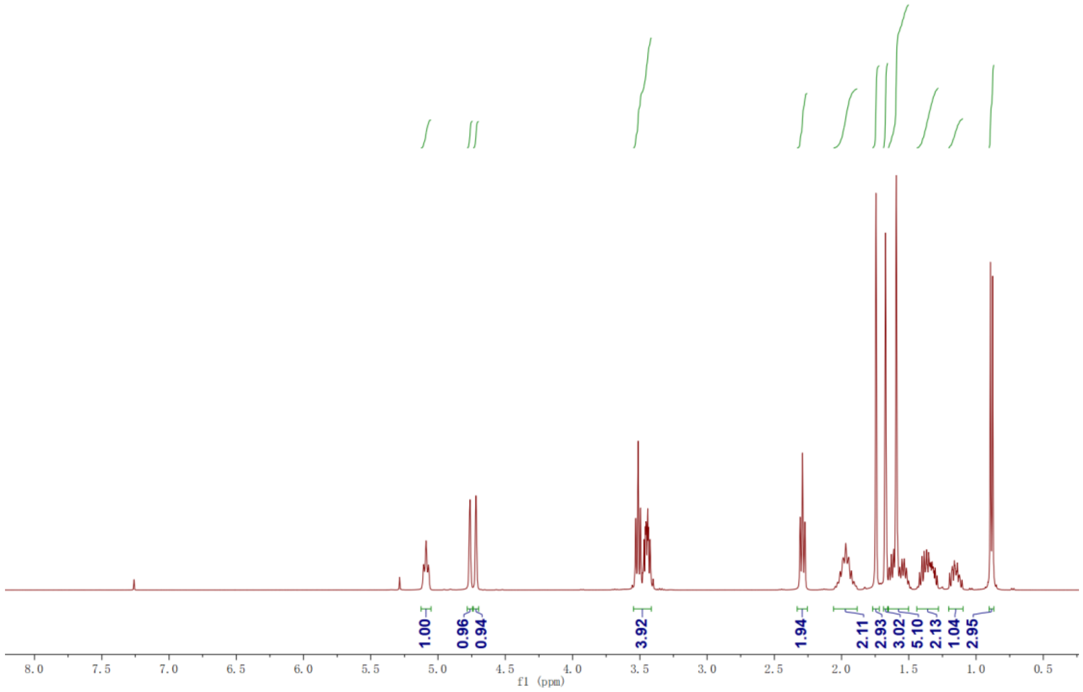

Supplementary Figure 10. ^{1}H NMR spectrum of 2o

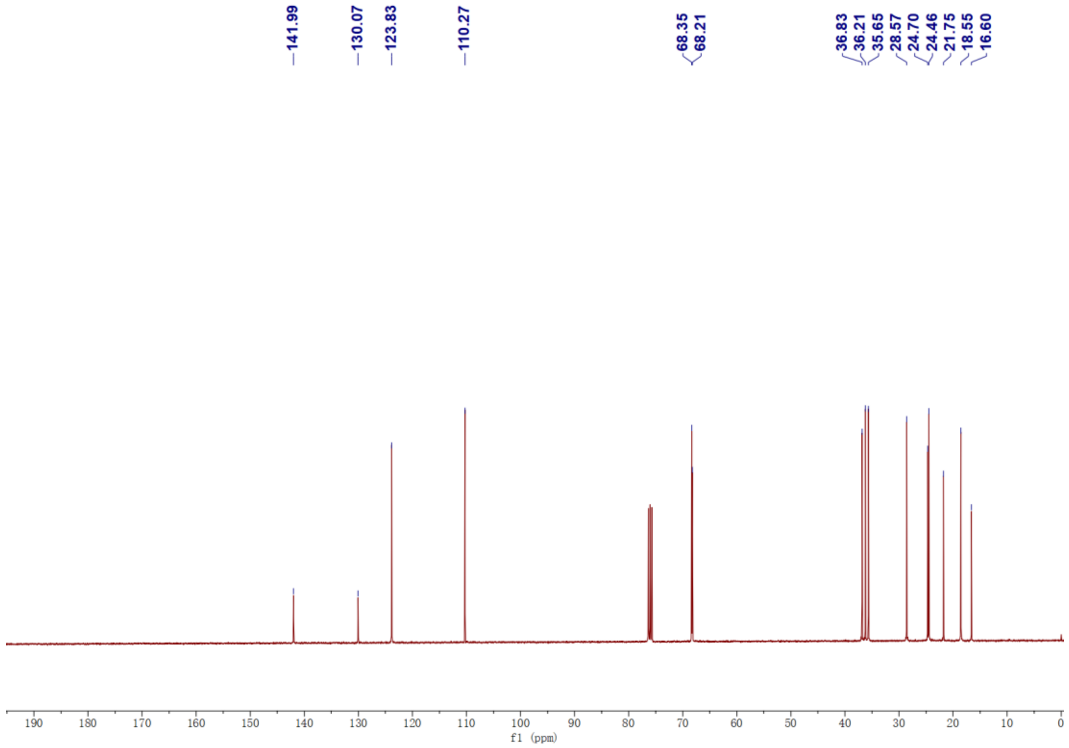

Supplementary Figure 11. ^{13}C NMR spectrum of 2o

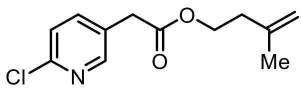

2t

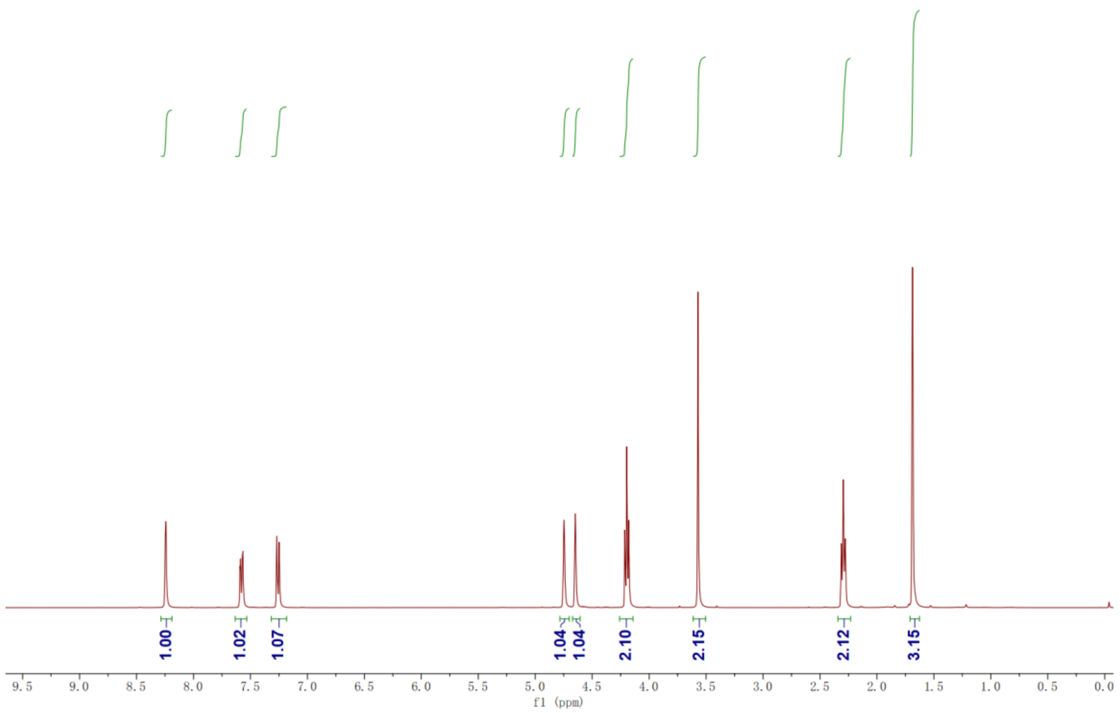

Supplementary Figure 12. ^{1}**H NMR spectrum of 2t**

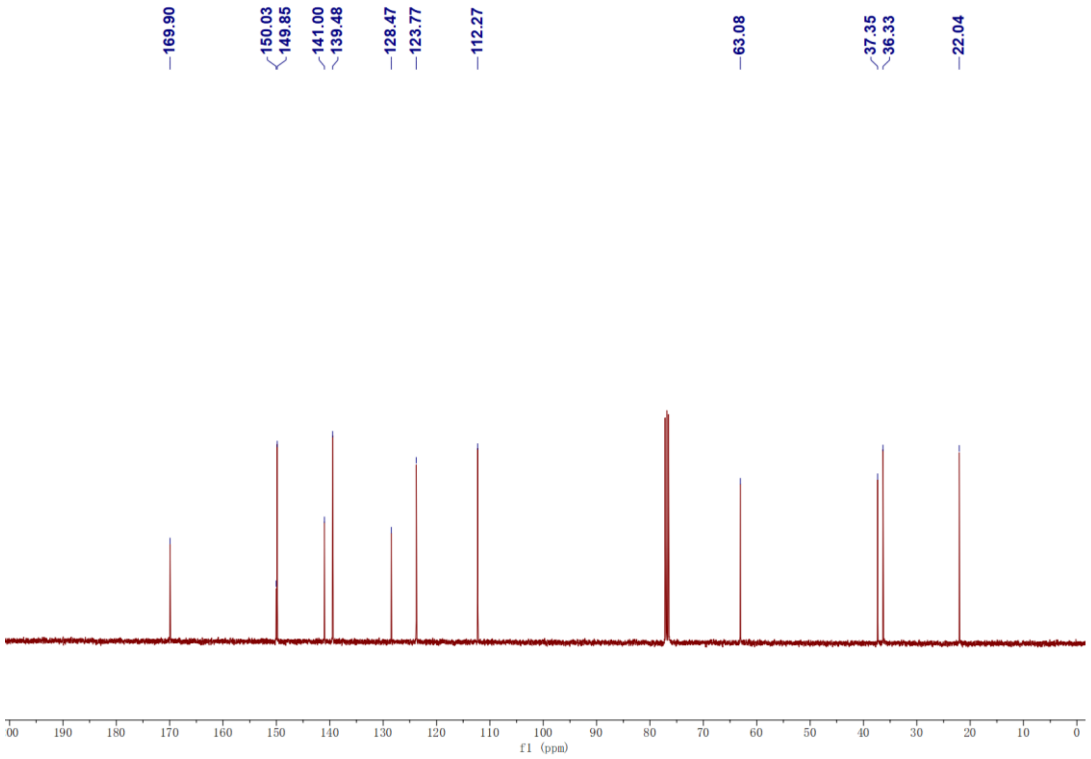

Supplementary Figure 13. ^{13}**C NMR spectrum of 2t**

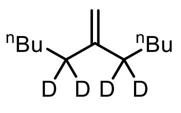

2w-d_4

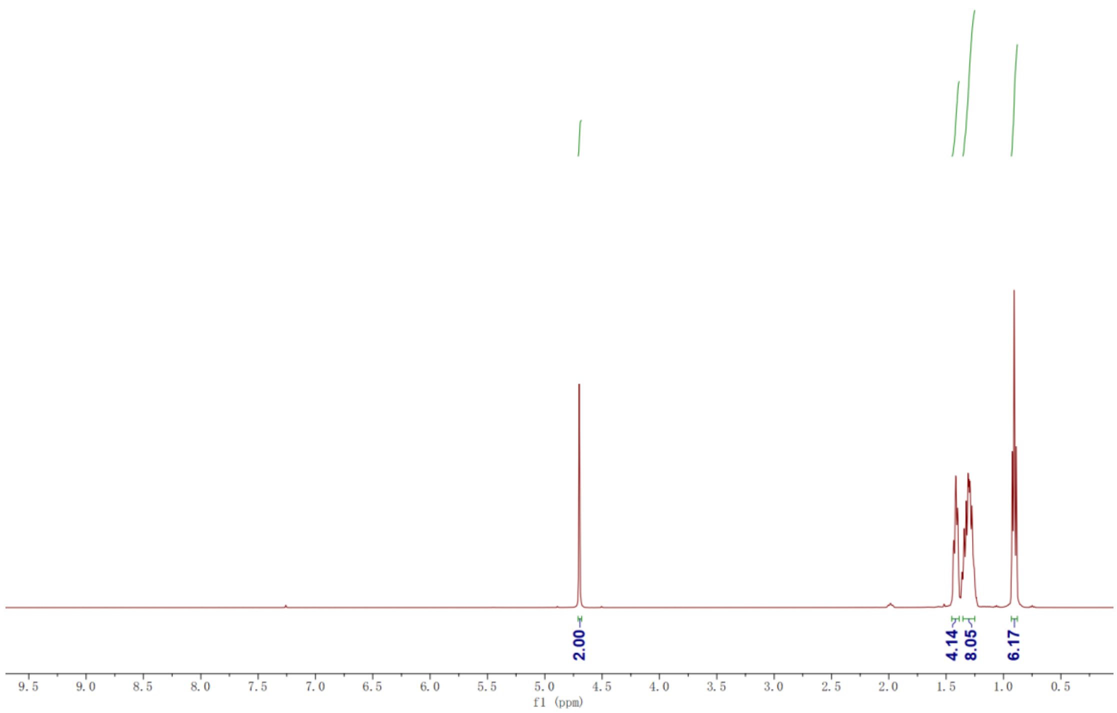

Supplementary Figure 14. ^{1}H NMR spectrum of 2w-d_4

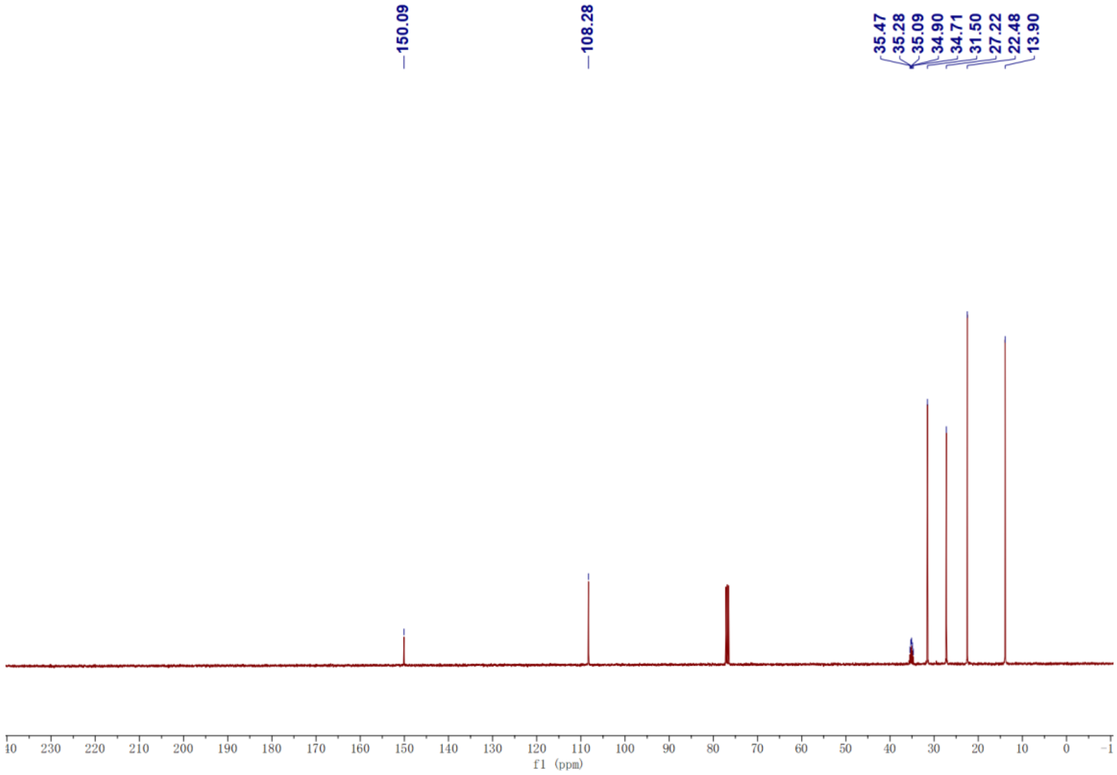

Supplementary Figure 15. ^{13}C NMR spectrum of 2w-d_4

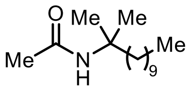

3a

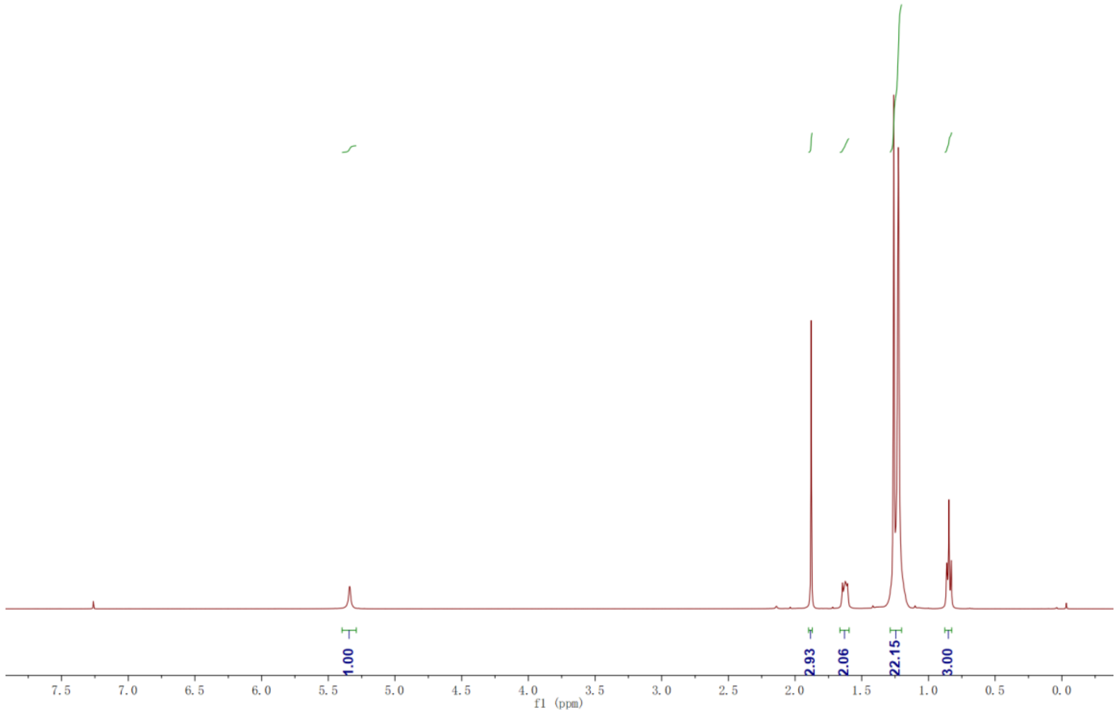

Supplementary Figure 16. ^{1}**H NMR spectrum of 3a**

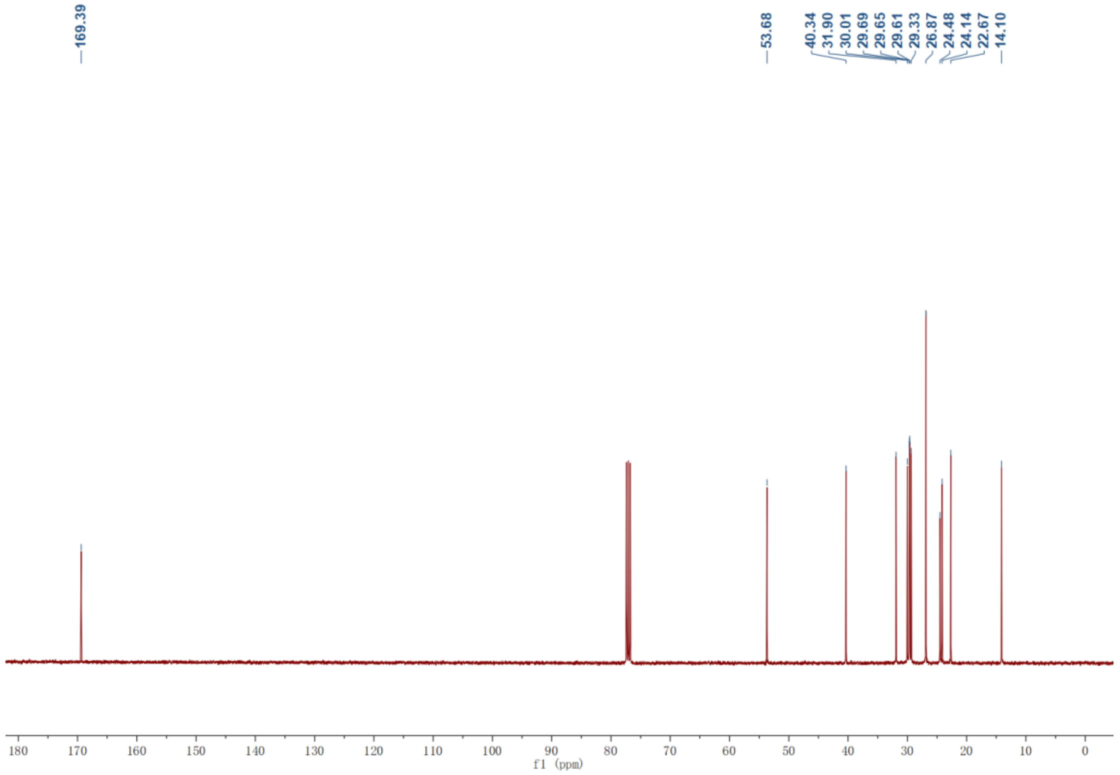

Supplementary Figure 17. ^{13}**C NMR spectrum of 3a**

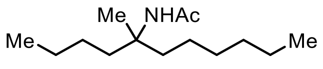

3b

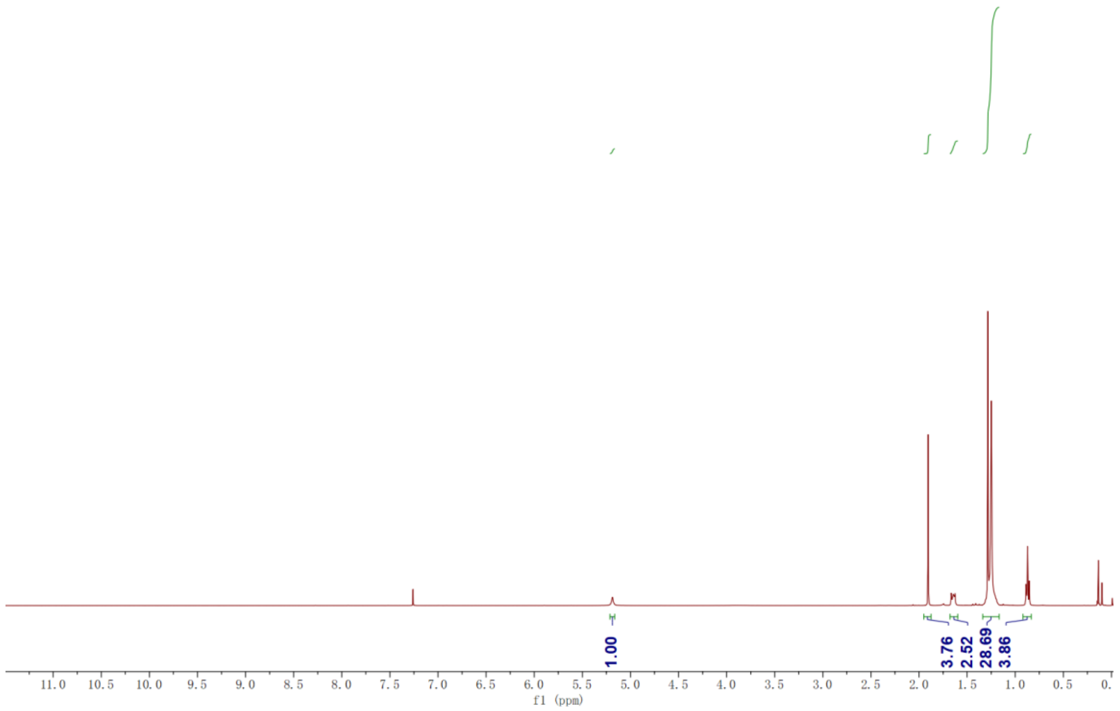

Supplementary Figure 18. ^{1}H NMR spectrum of 3b

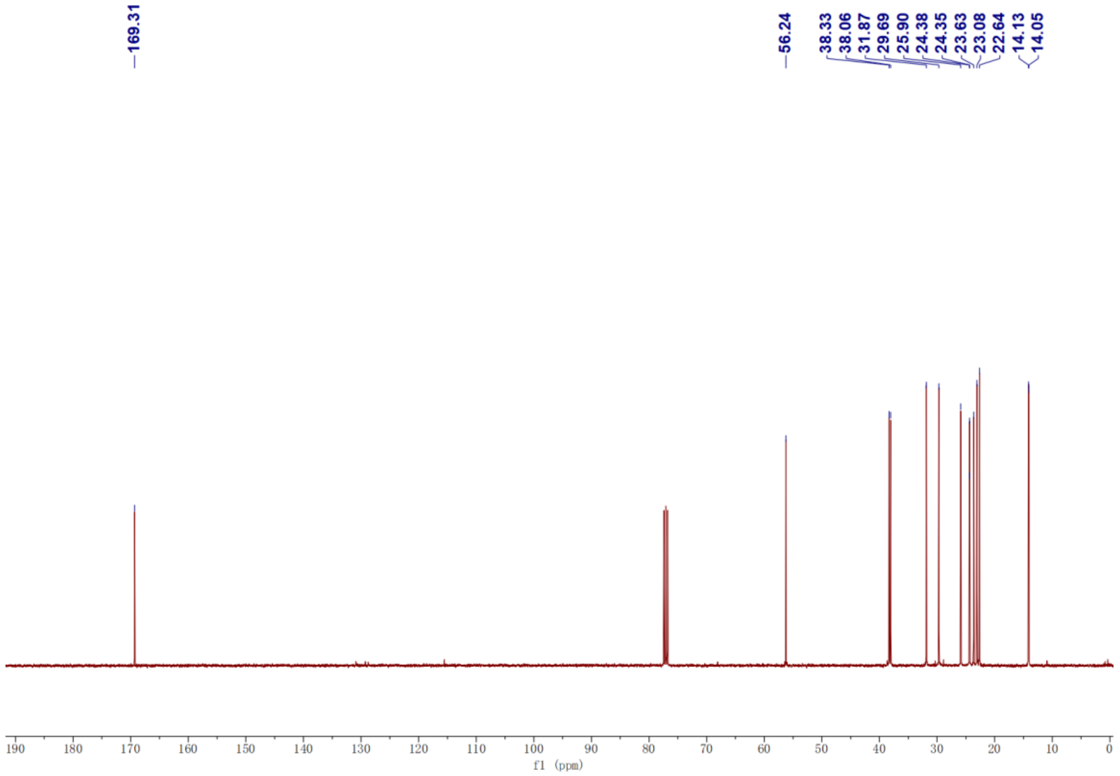

Supplementary Figure 19. ^{13}C NMR spectrum of 3b

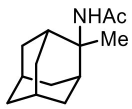

3c

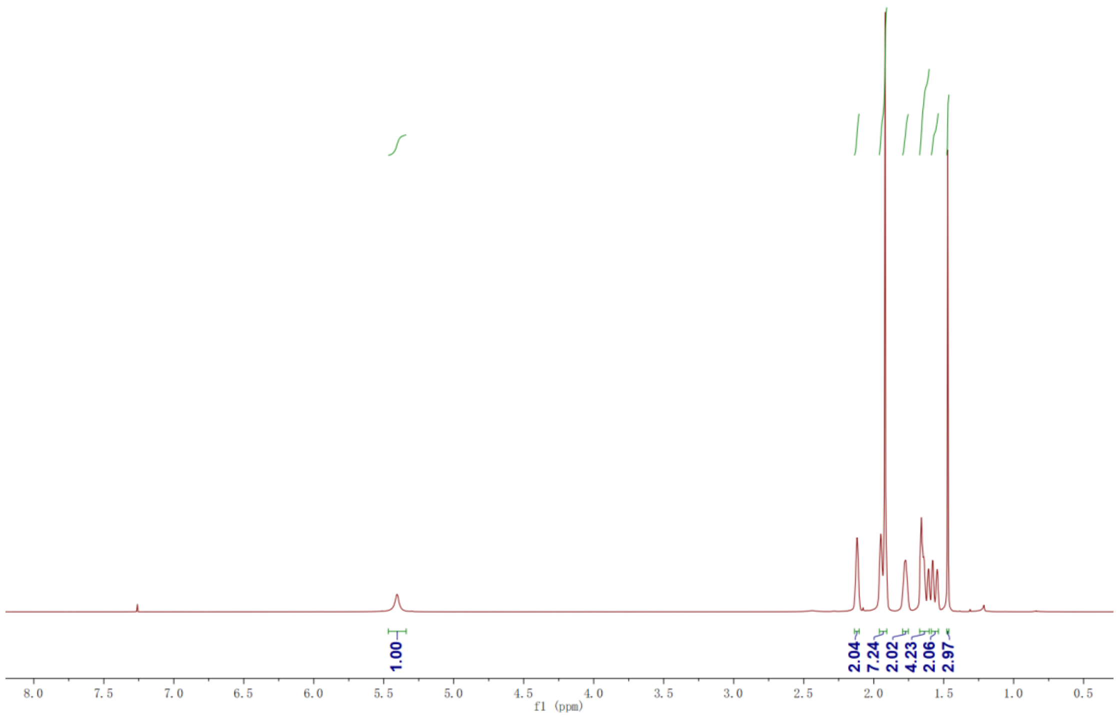

Supplementary Figure 20. ^{1}H NMR spectrum of 3c

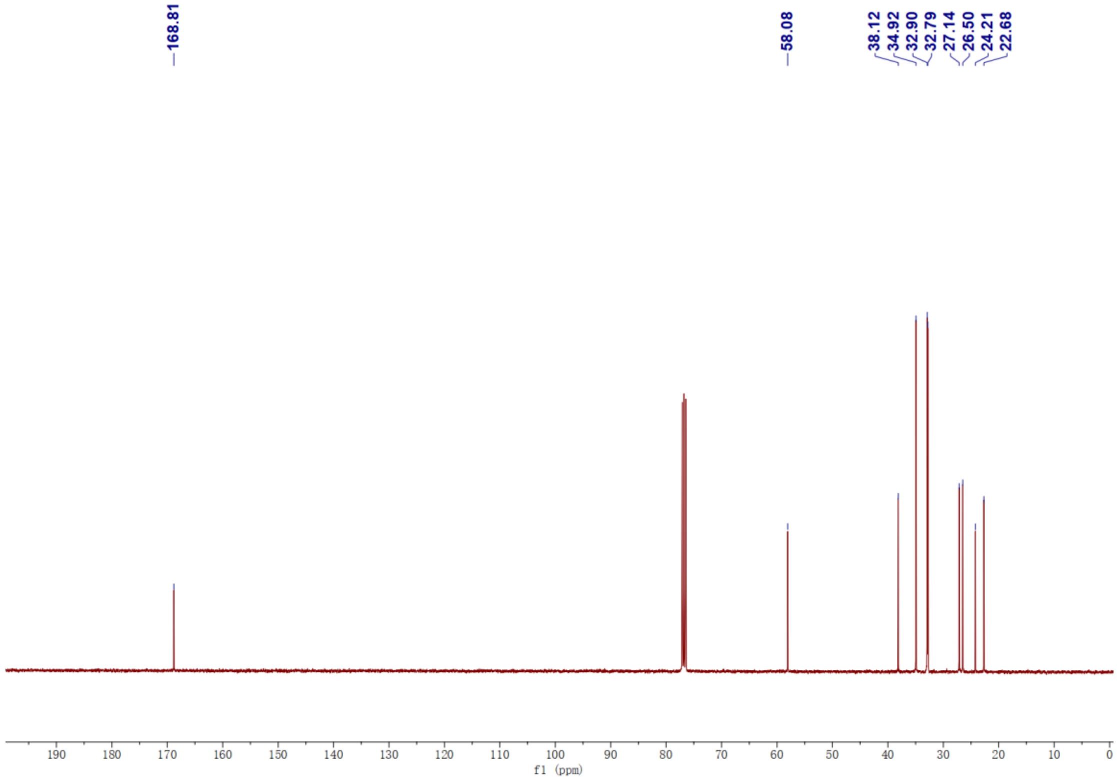

Supplementary Figure 21. ^{13}C NMR spectrum of 3c

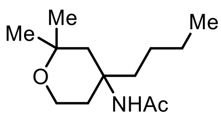

3d

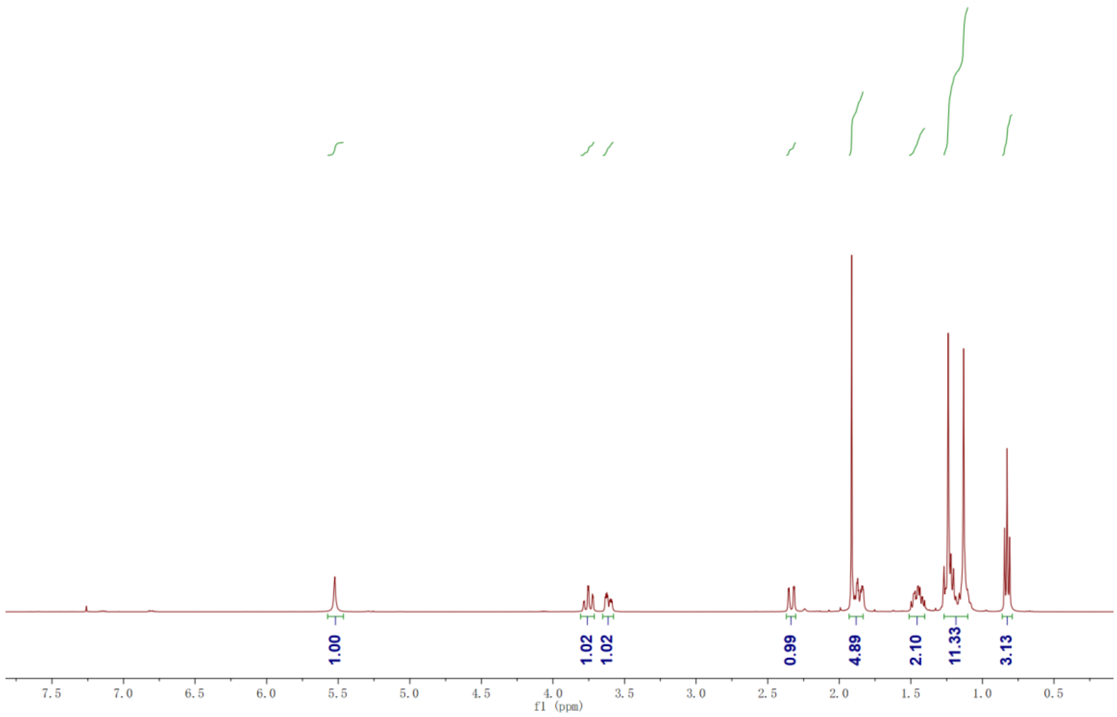

Supplementary Figure 22. ^{1}**H NMR spectrum of 3d**

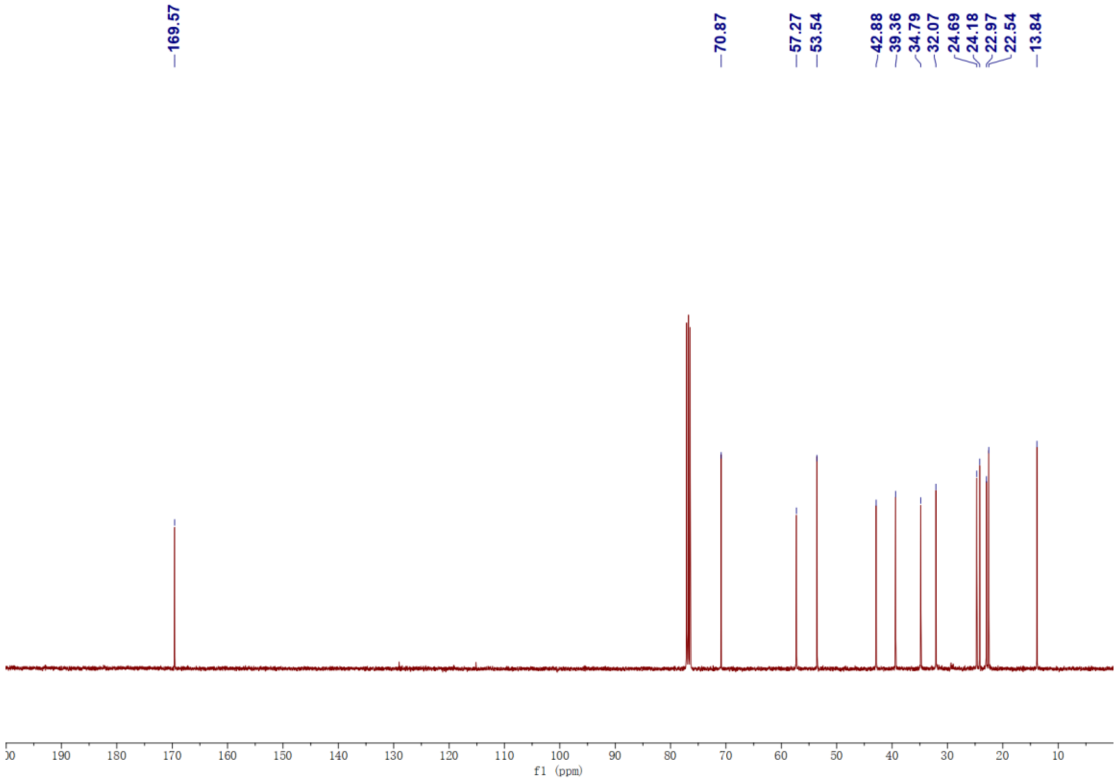

Supplementary Figure 23. ^{13}**C NMR spectrum of 3d**

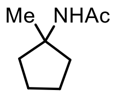

3e

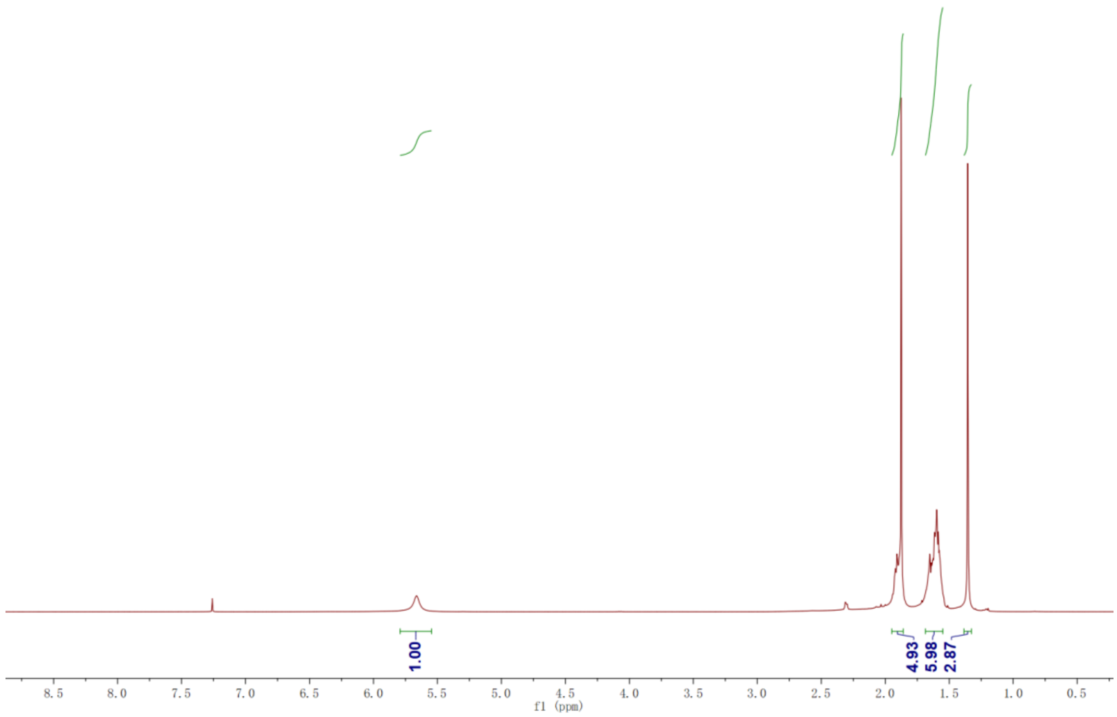

Supplementary Figure 24. ^{1}H NMR spectrum of 3e

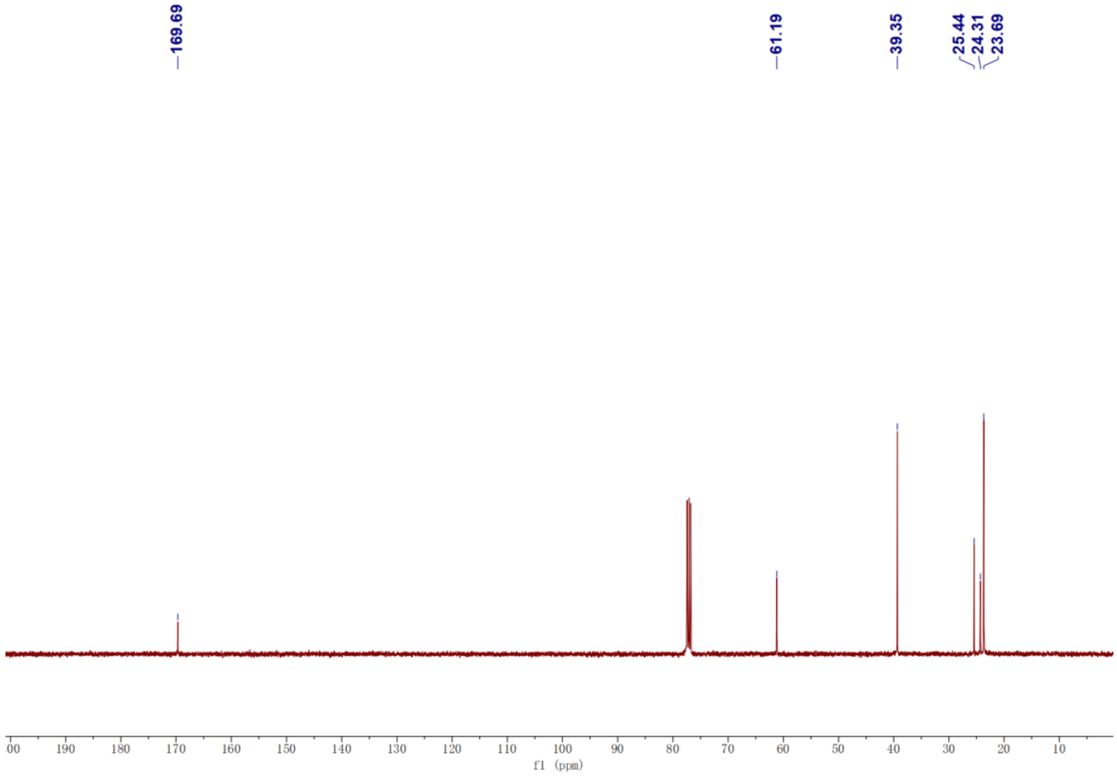

Supplementary Figure 25. ^{13}C NMR spectrum of 3e

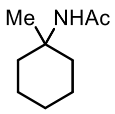

3f

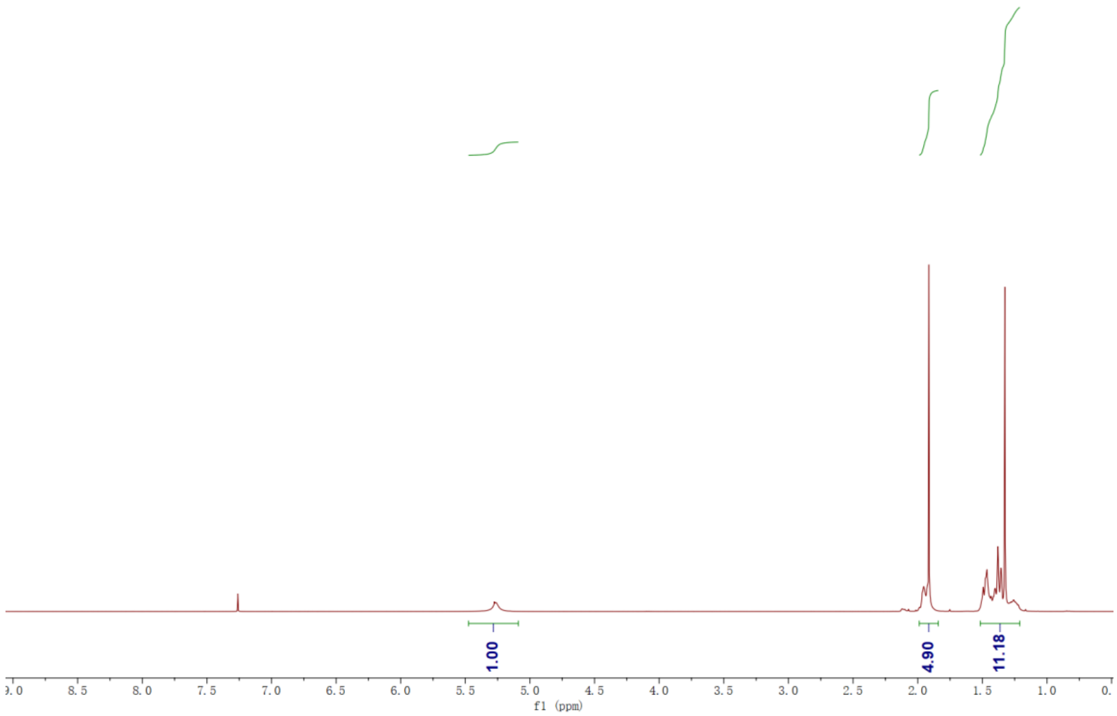

Supplementary Figure 26. ^{1}H NMR spectrum of 3f

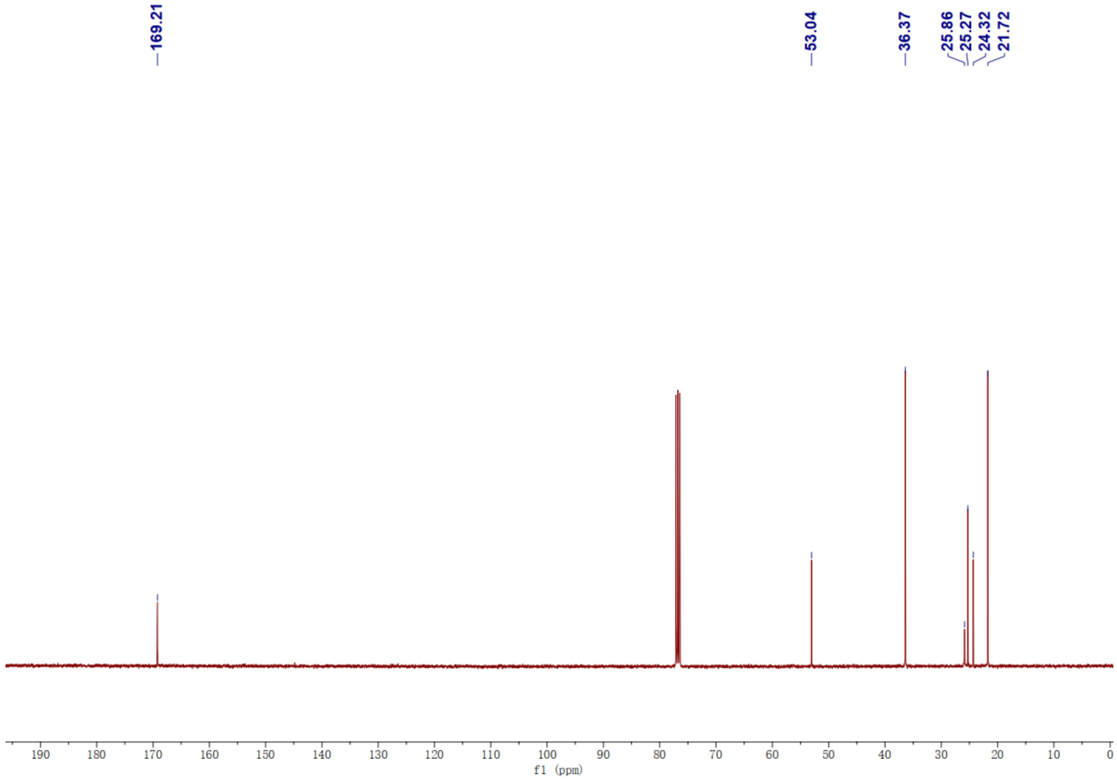

Supplementary Figure 27. ^{13}C NMR spectrum of 3f

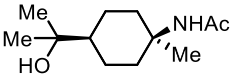

3h

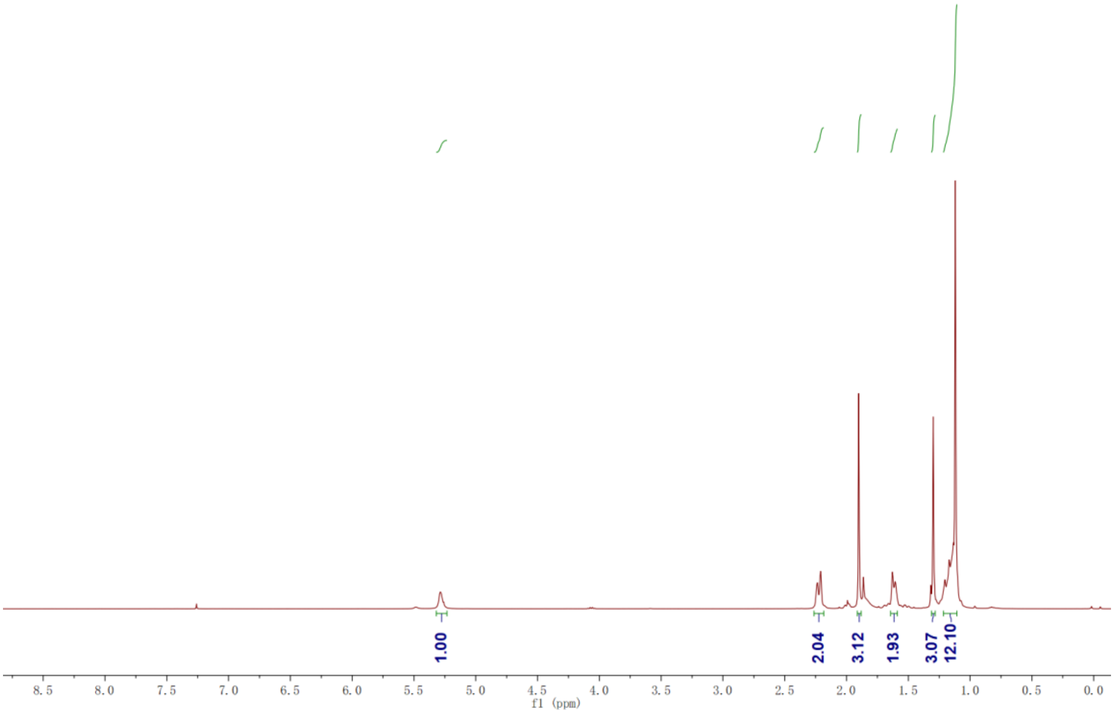

Supplementary Figure 28. ^{1}H NMR spectrum of 3h

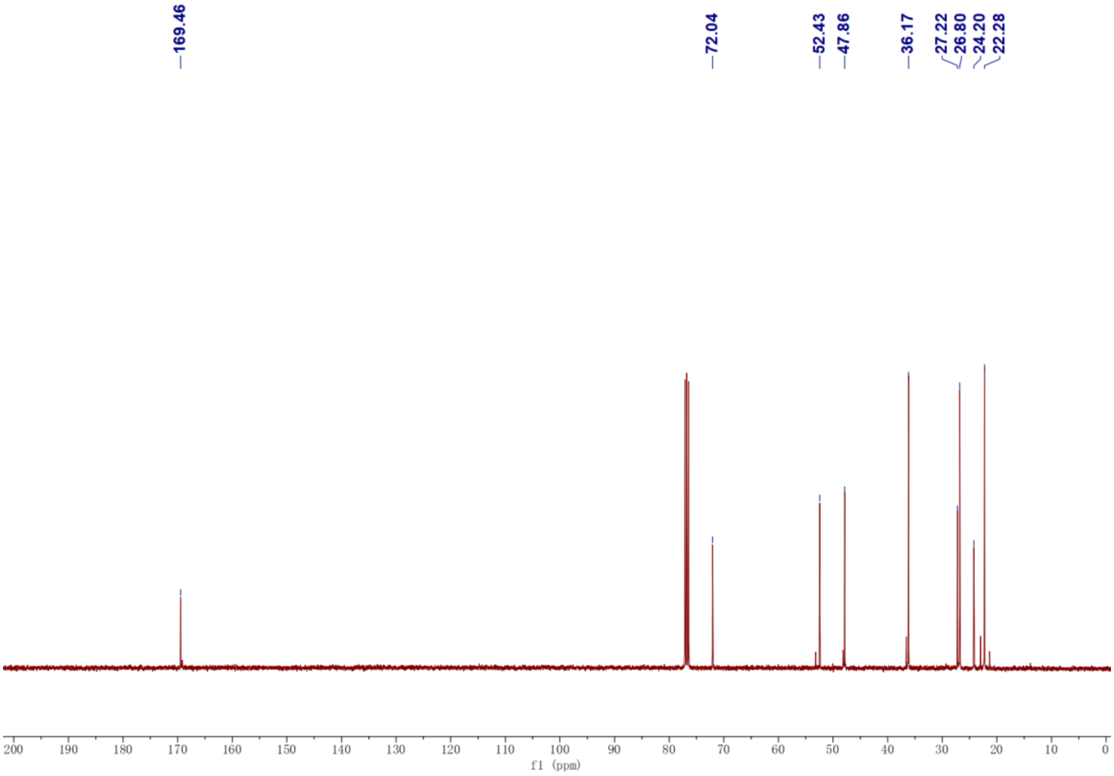

Supplementary Figure 29. ^{13}C NMR spectrum of 3h

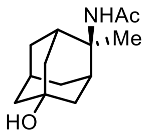

3i

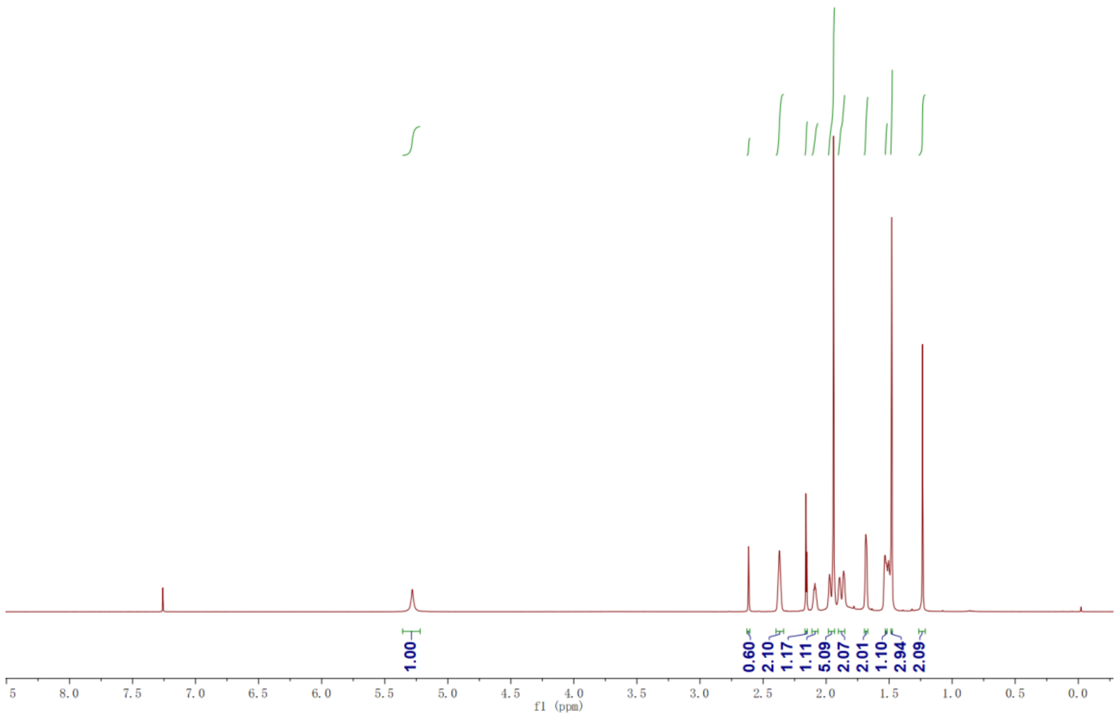

Supplementary Figure 30. ^{1}H NMR spectrum of 3i

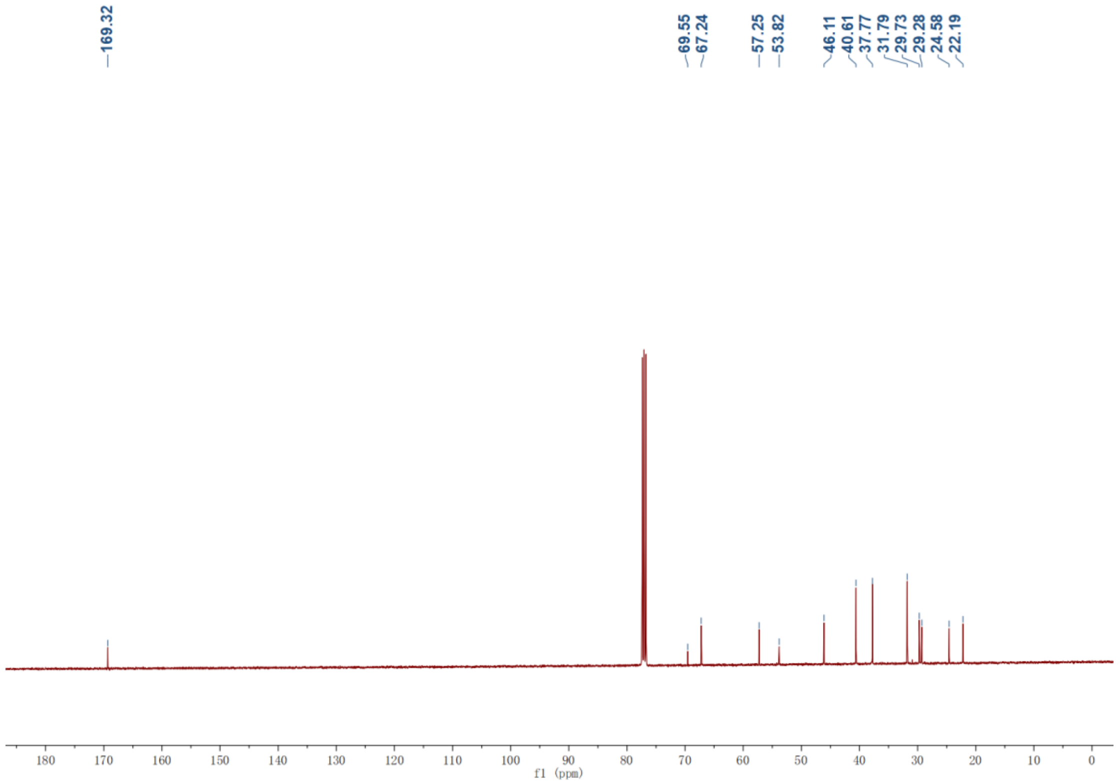

Supplementary Figure 31. ^{13}C NMR spectrum of 3i

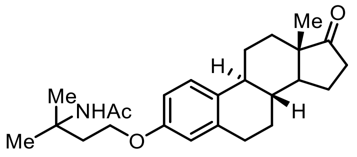

3j

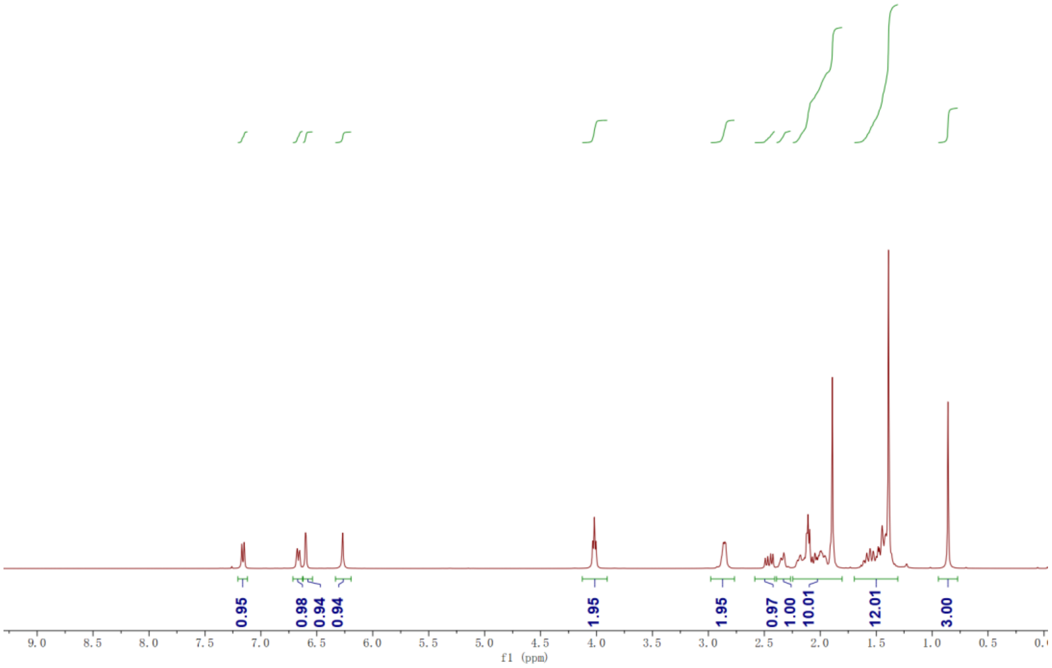

Supplementary Figure 32. ^{1}H NMR spectrum of 3j

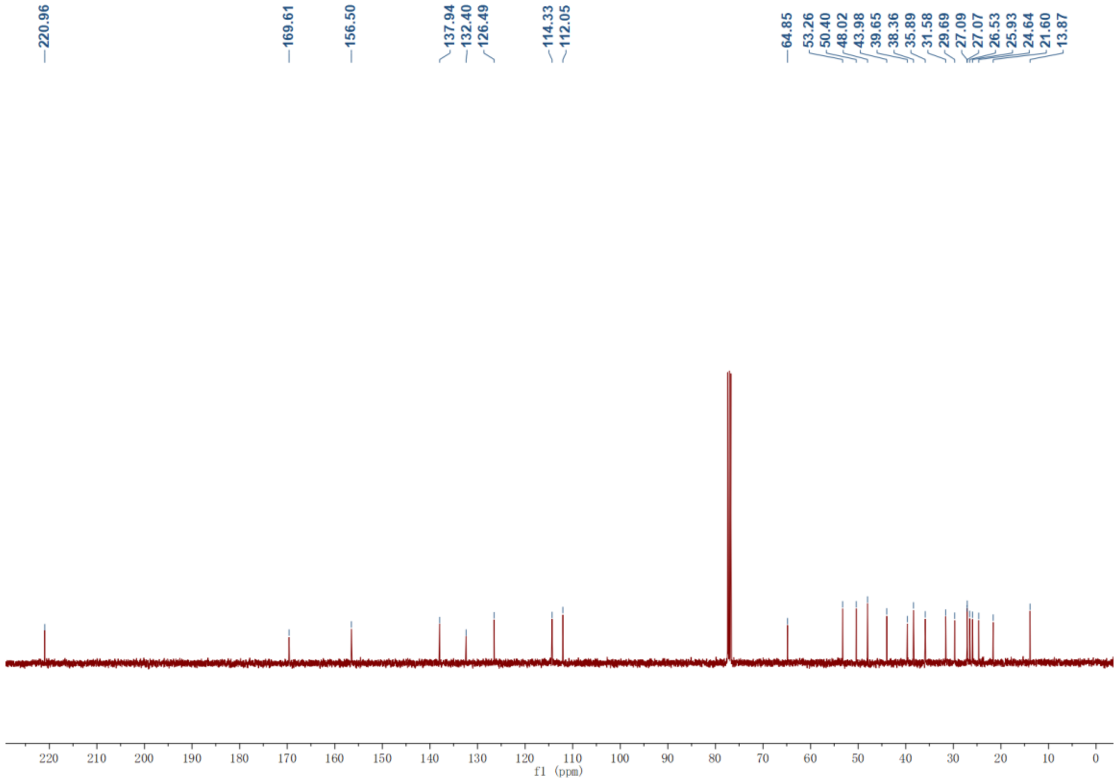

Supplementary Figure 33. ^{13}C NMR spectrum of 3j

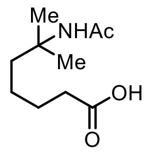

3k

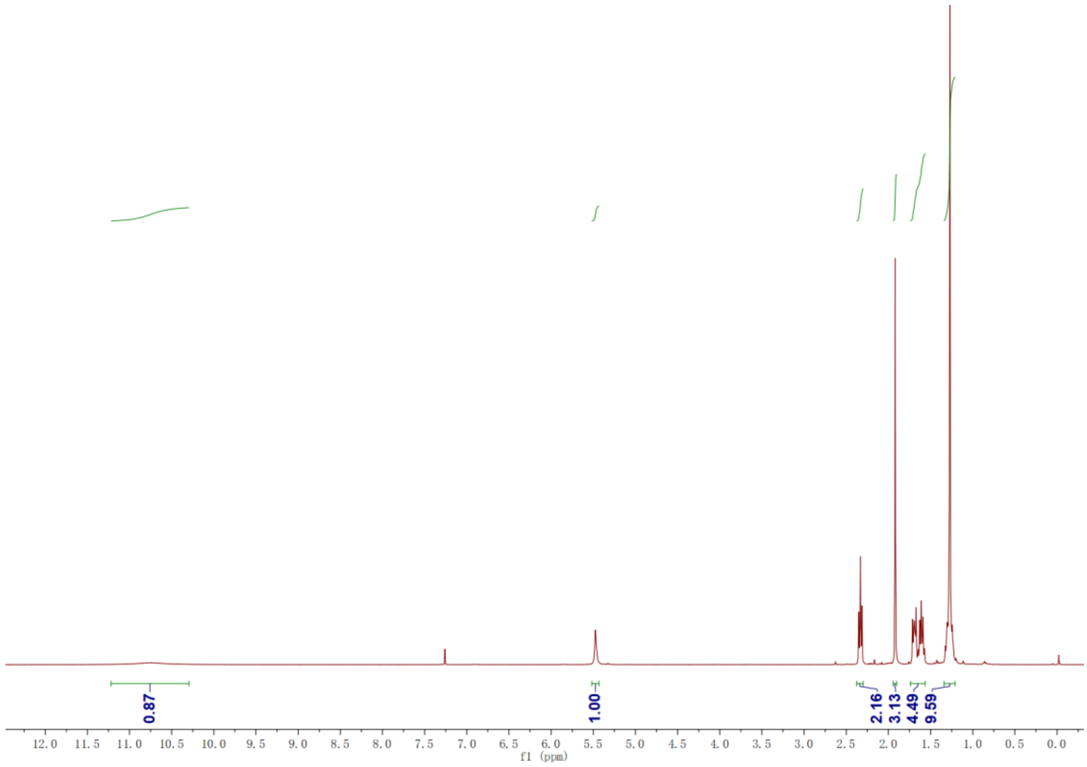

Supplementary Figure 34. ^{1}H **NMR spectrum of 3k**

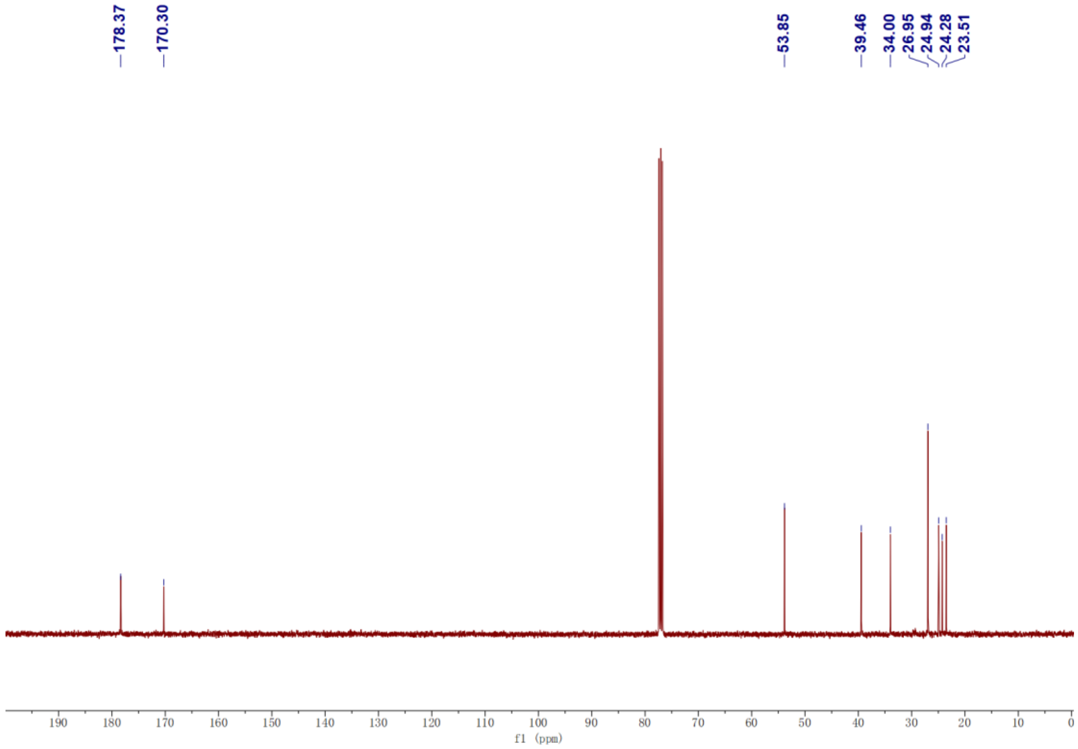

Supplementary Figure 35. ^{13}C **NMR spectrum of 3k**

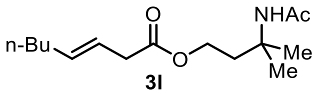

3l

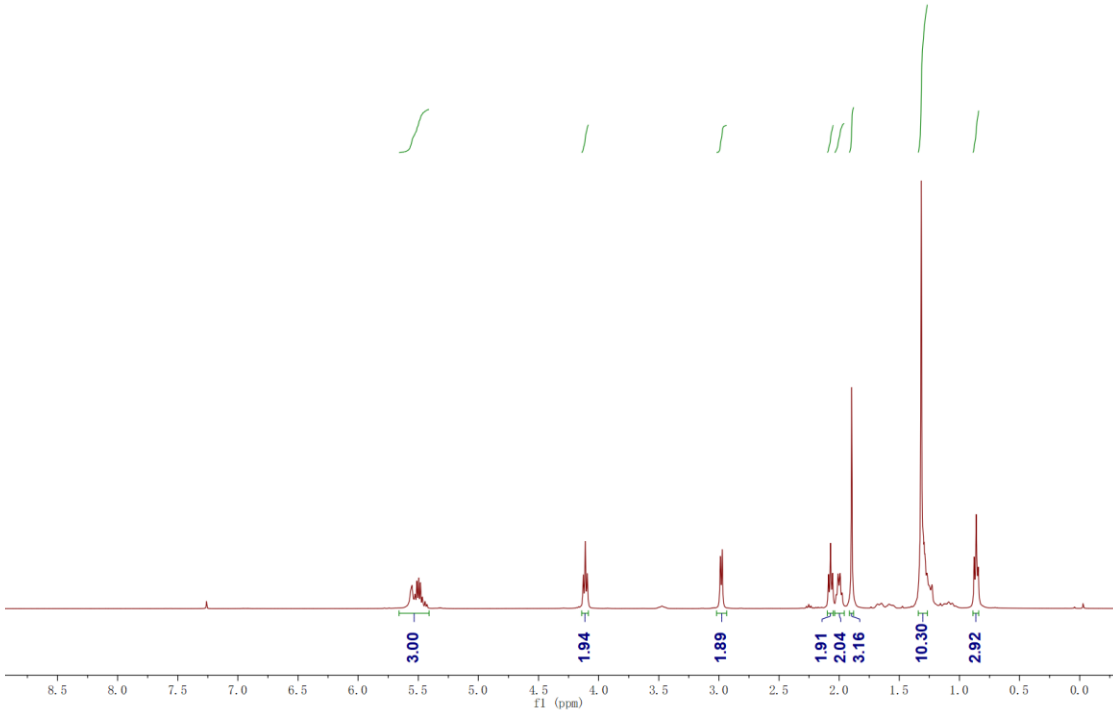

Supplementary Figure 36. **^{1}H NMR spectrum of 3l**

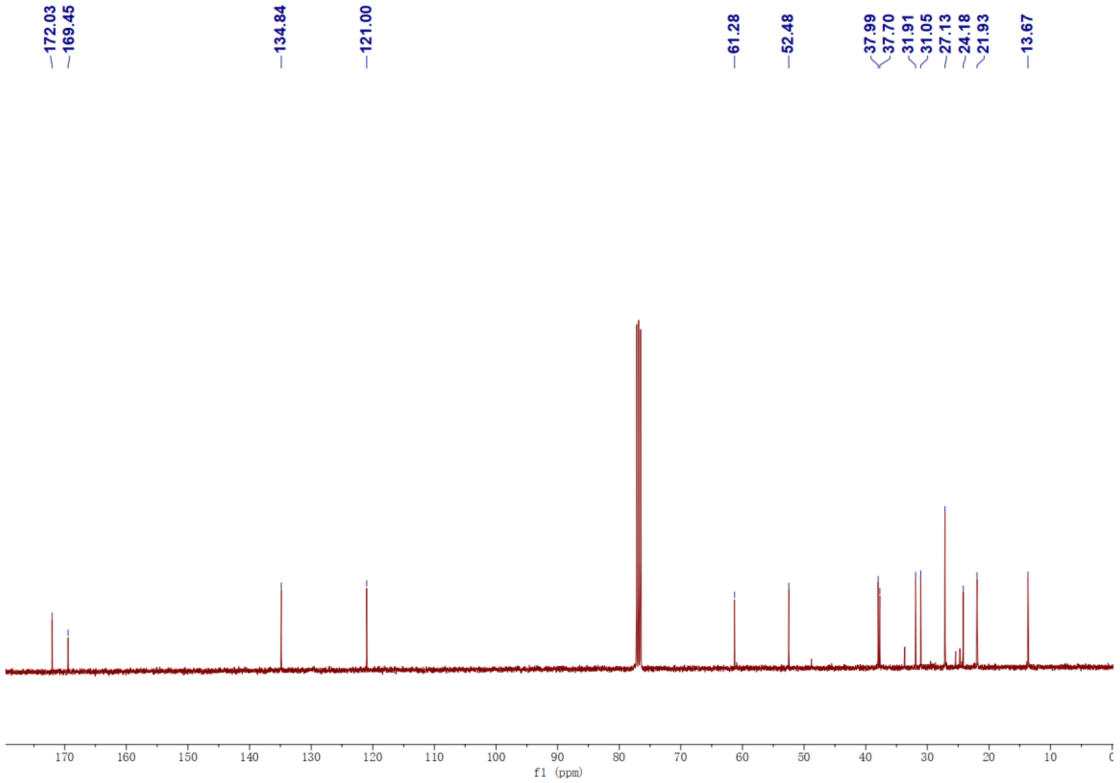

Supplementary Figure 37. **^{13}C NMR spectrum of 3l**

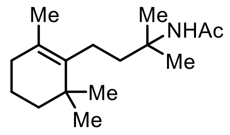

3m

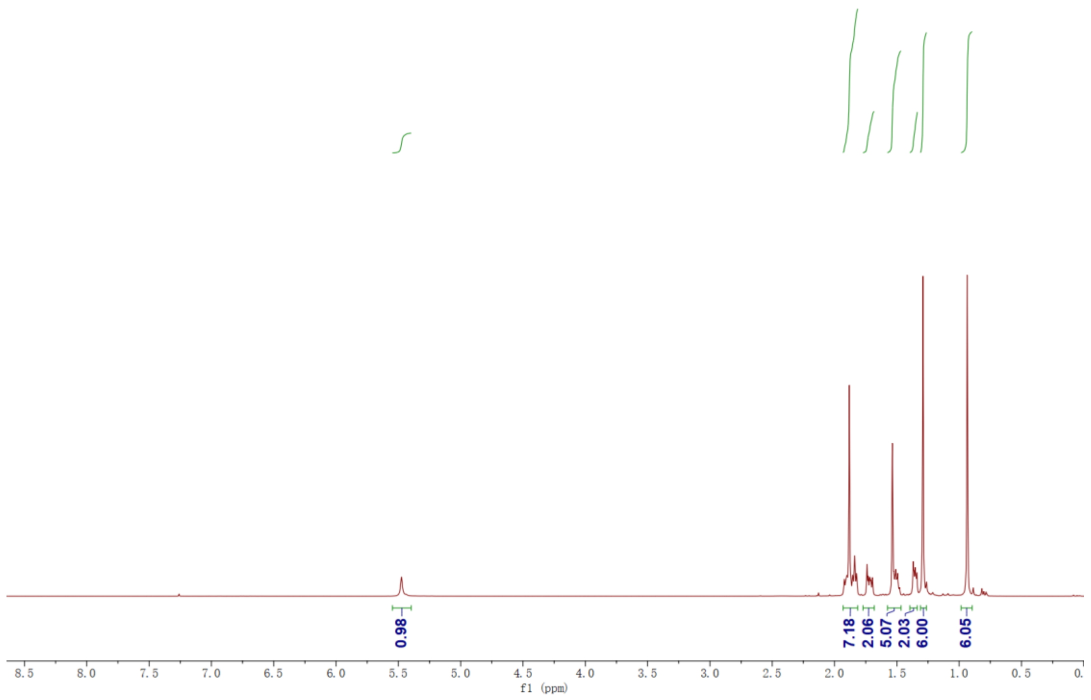

Supplementary Figure 38. ^{1}H NMR spectrum of 3m

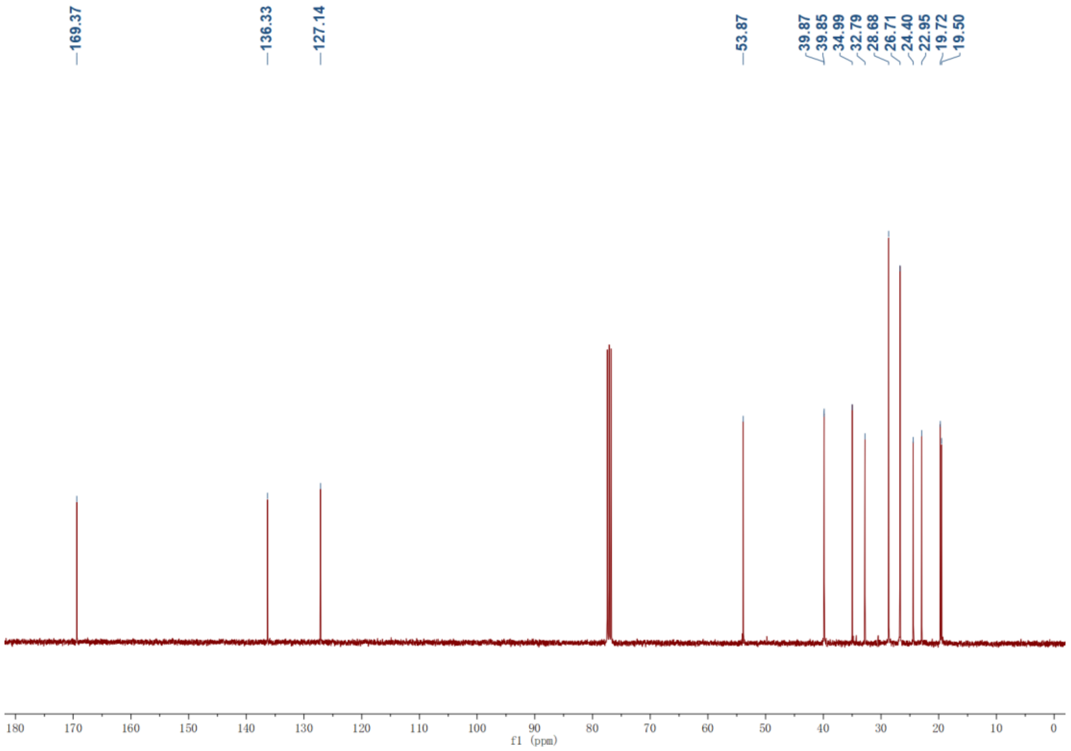

Supplementary Figure 39. ^{13}C NMR spectrum of 3m

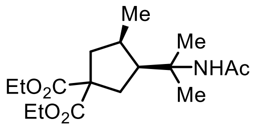

3n

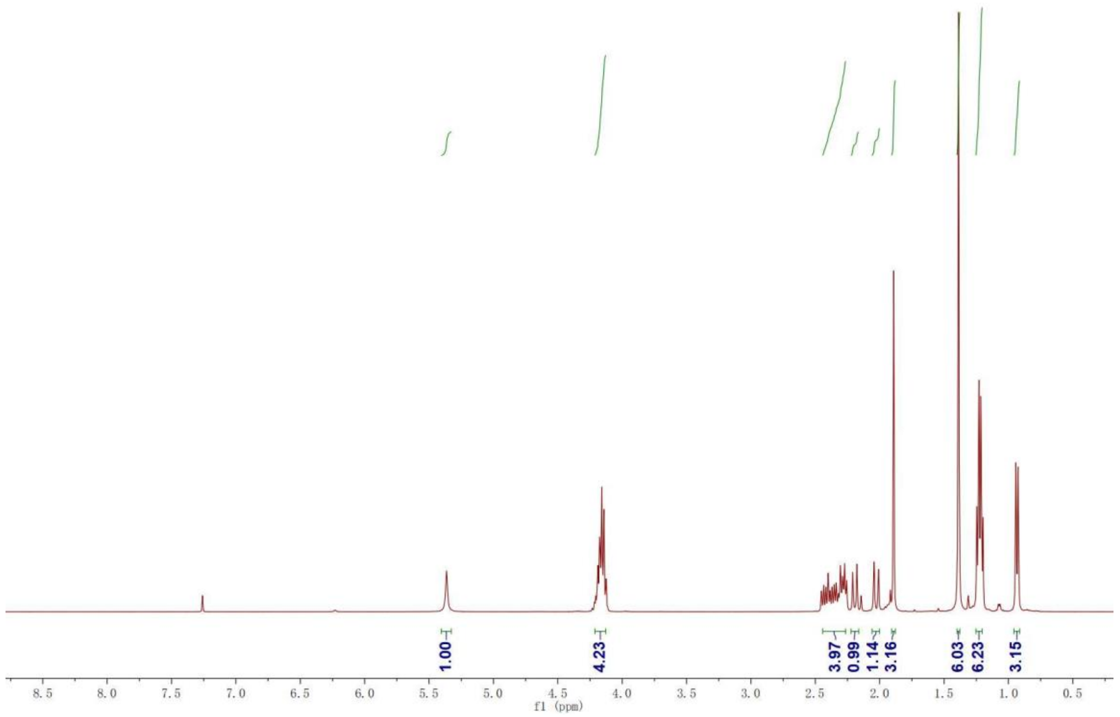

Supplementary Figure 40. ^{1}H NMR spectrum of 3n

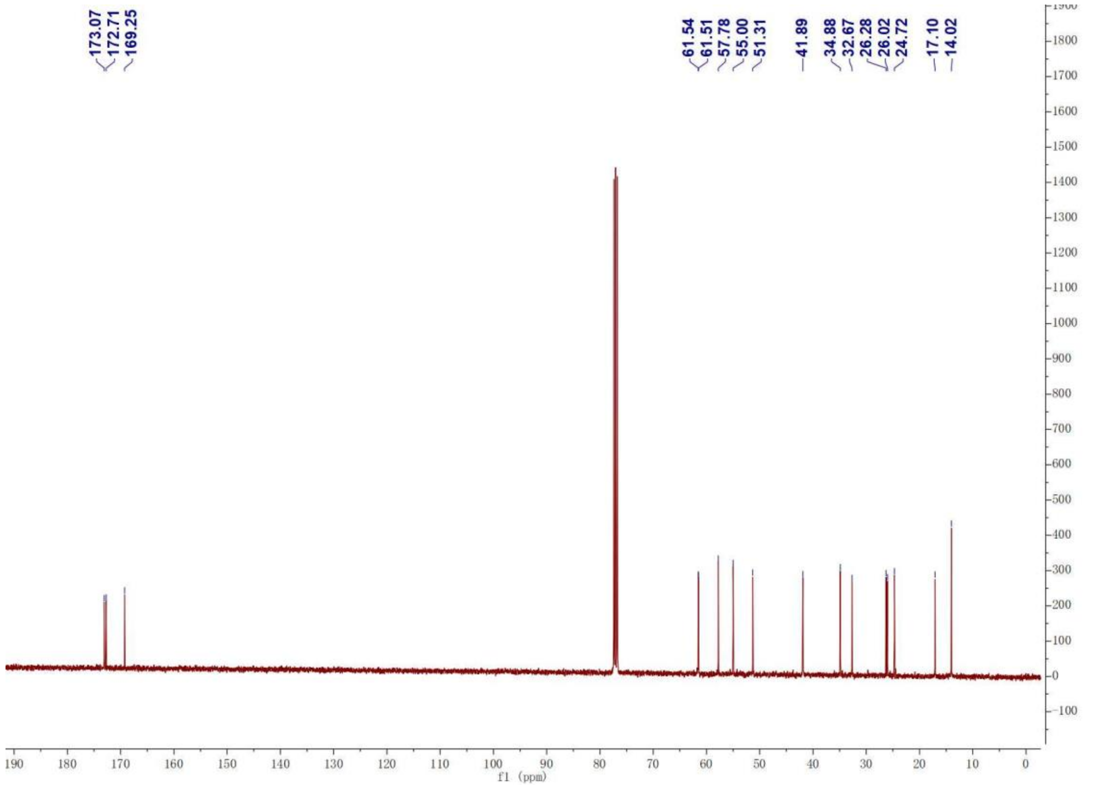

Supplementary Figure 41. ^{13}C NMR spectrum of 3n

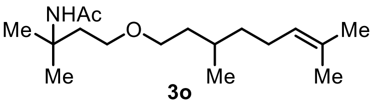

3o

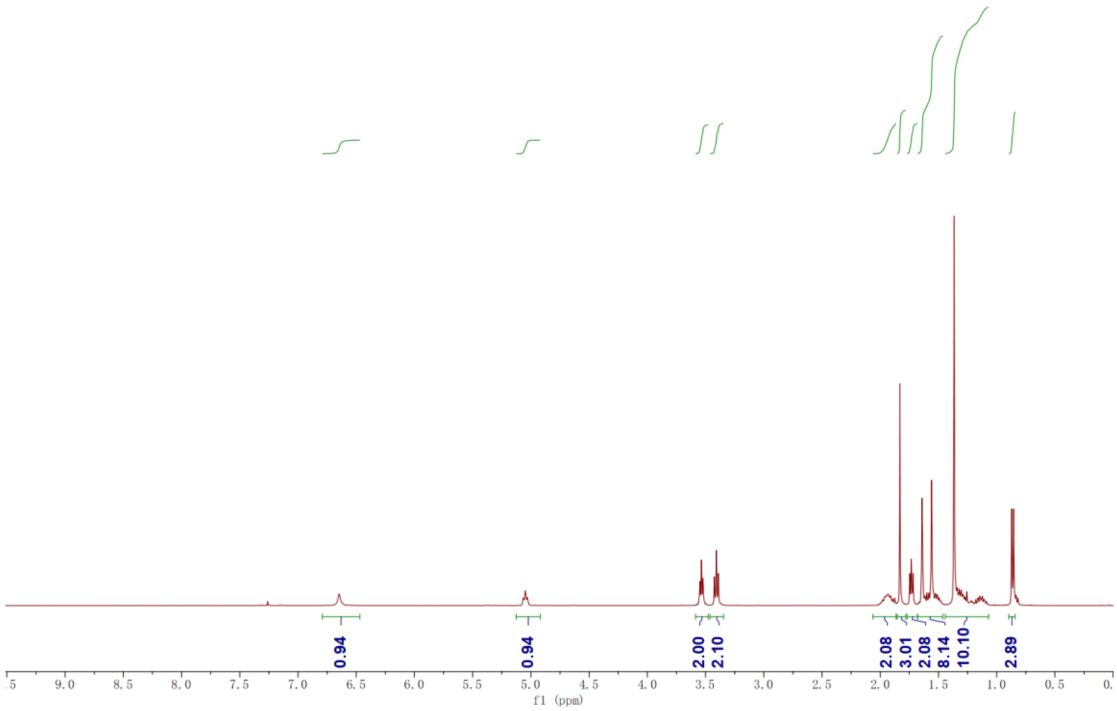

Supplementary Figure 42. ^{1}H NMR spectrum of 3o

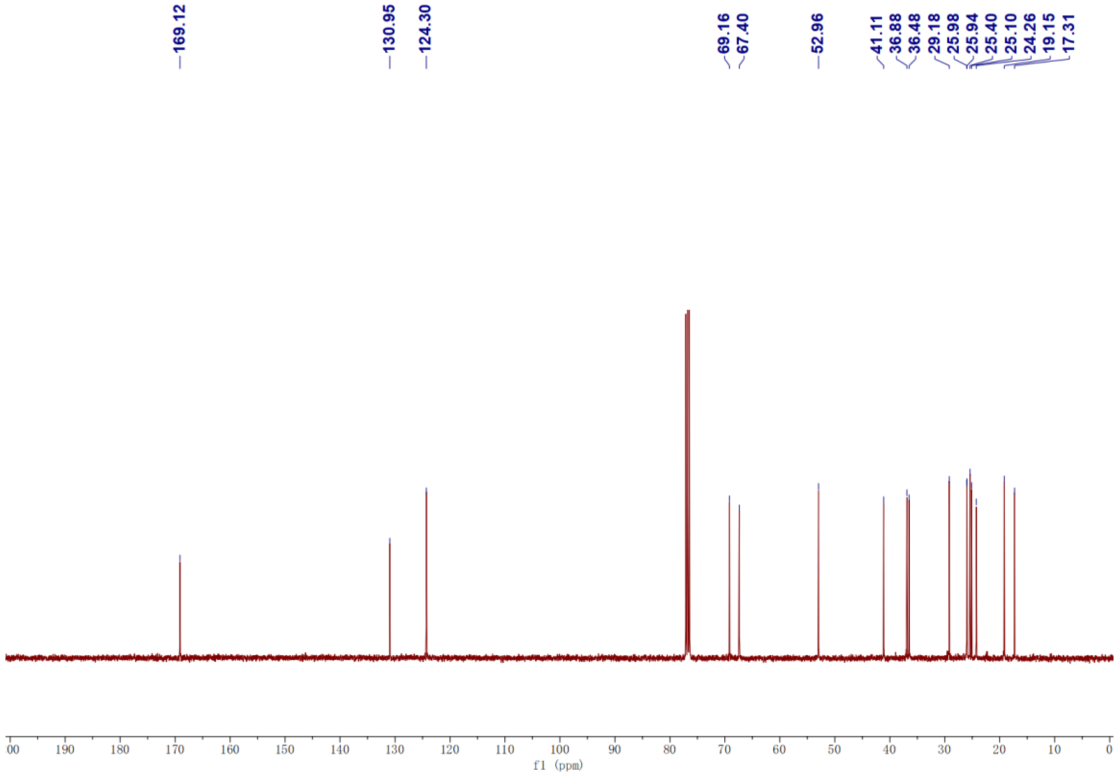

Supplementary Figure 43. ^{13}C NMR spectrum of 3o

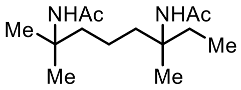

3o'

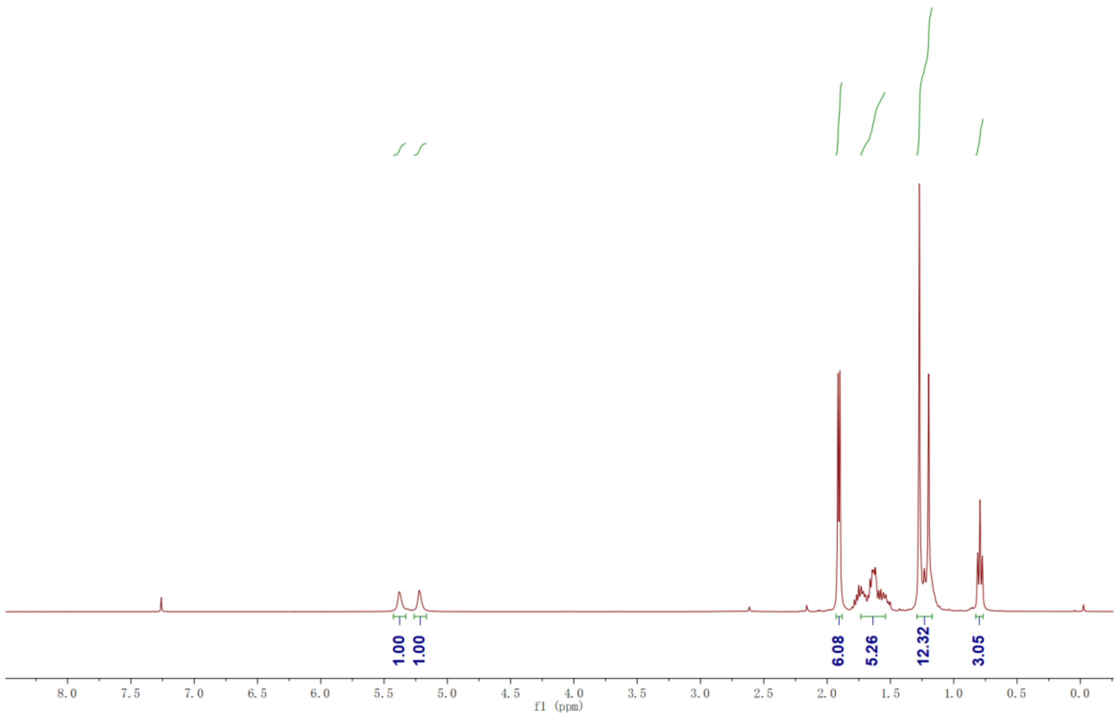

Supplementary Figure 44. ^{1}H NMR spectrum of 3o'

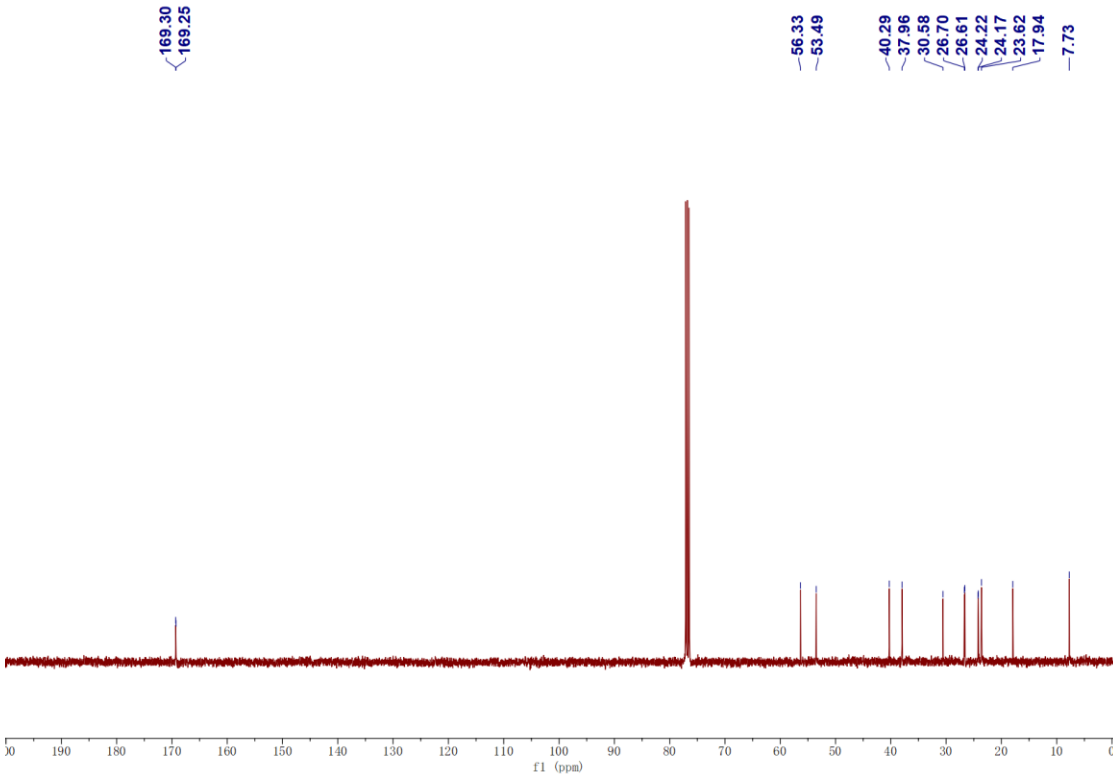

Supplementary Figure 45. ^{13}C NMR spectrum of 3o'

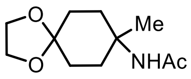

3p

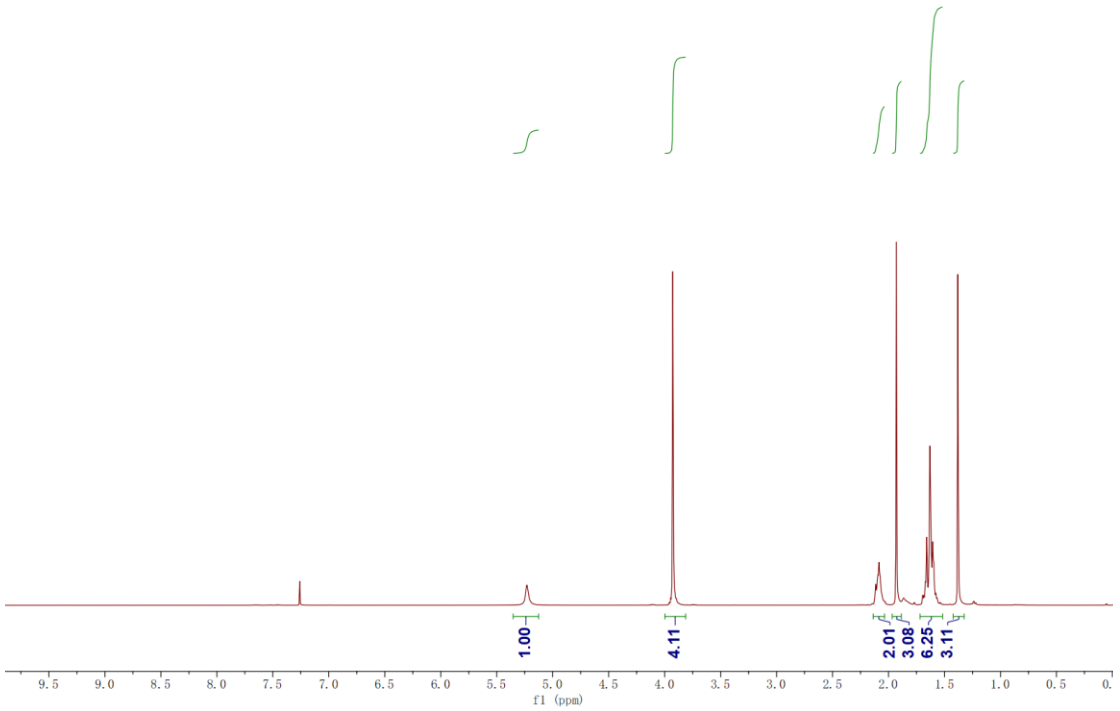

Supplementary Figure 46. ^{1}H NMR spectrum of 3p

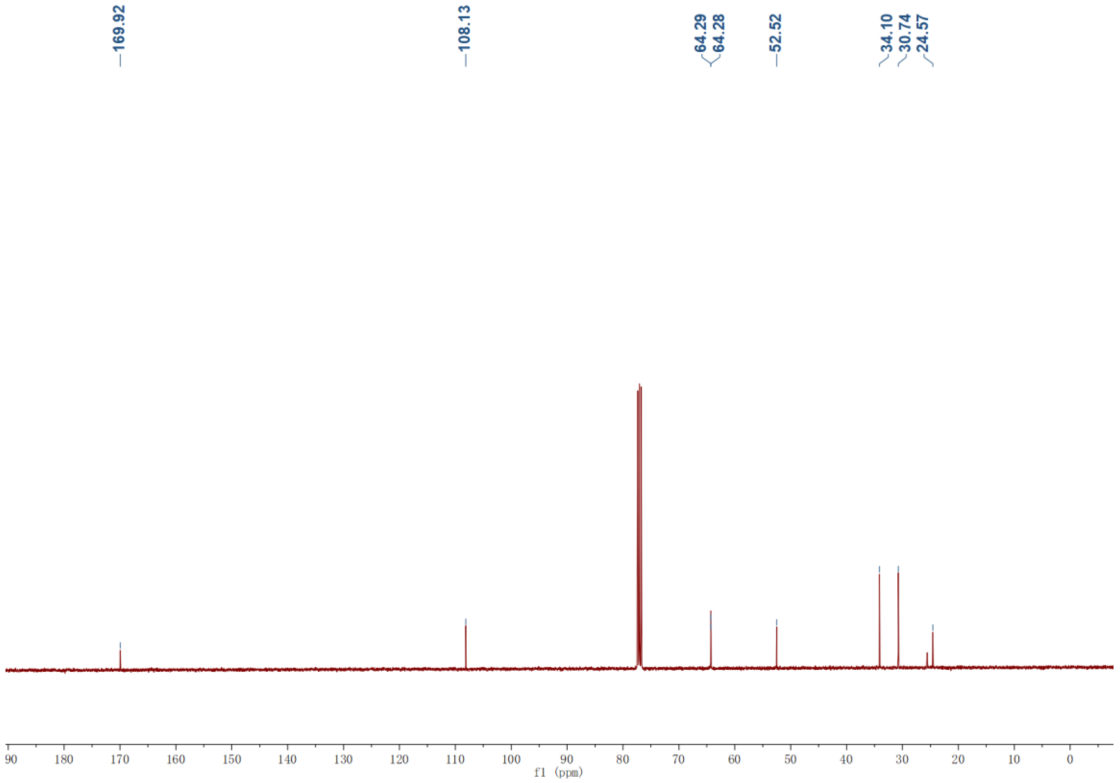

Supplementary Figure 47. ^{13}C NMR spectrum of 3p

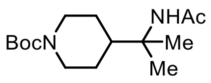

3q

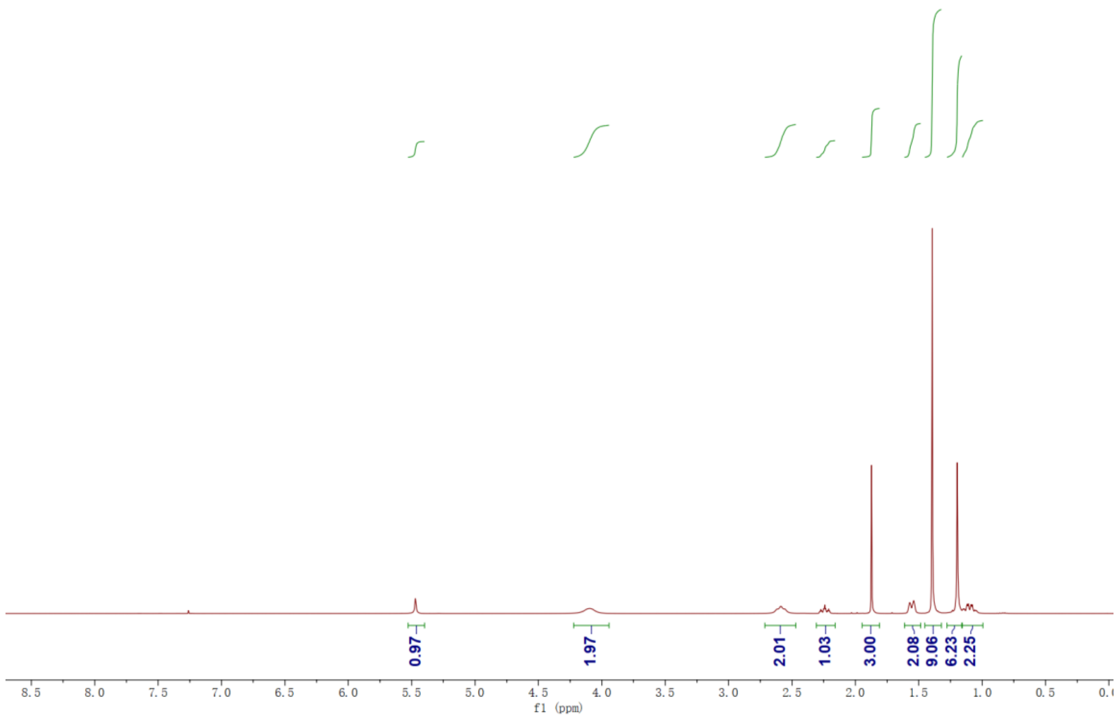

Supplementary Figure 48. ^{1}H NMR spectrum of 3q

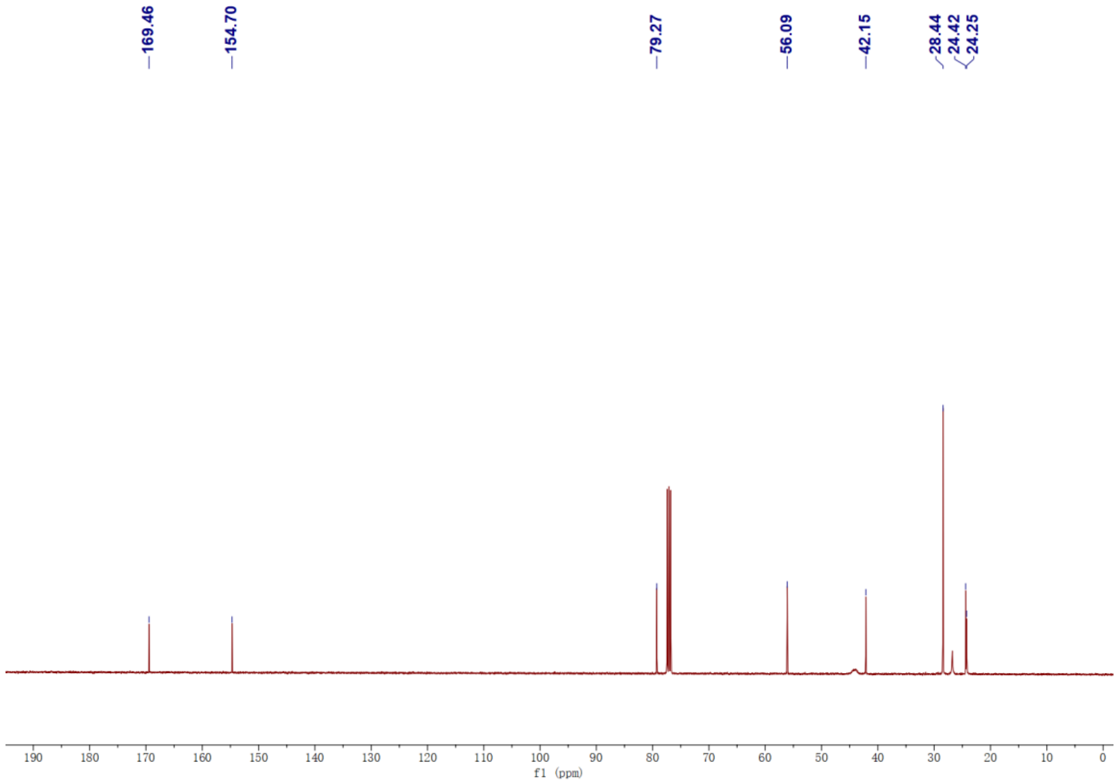

Supplementary Figure 49. ^{13}C NMR spectrum of 3q

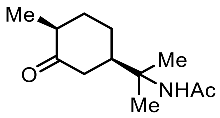

3r

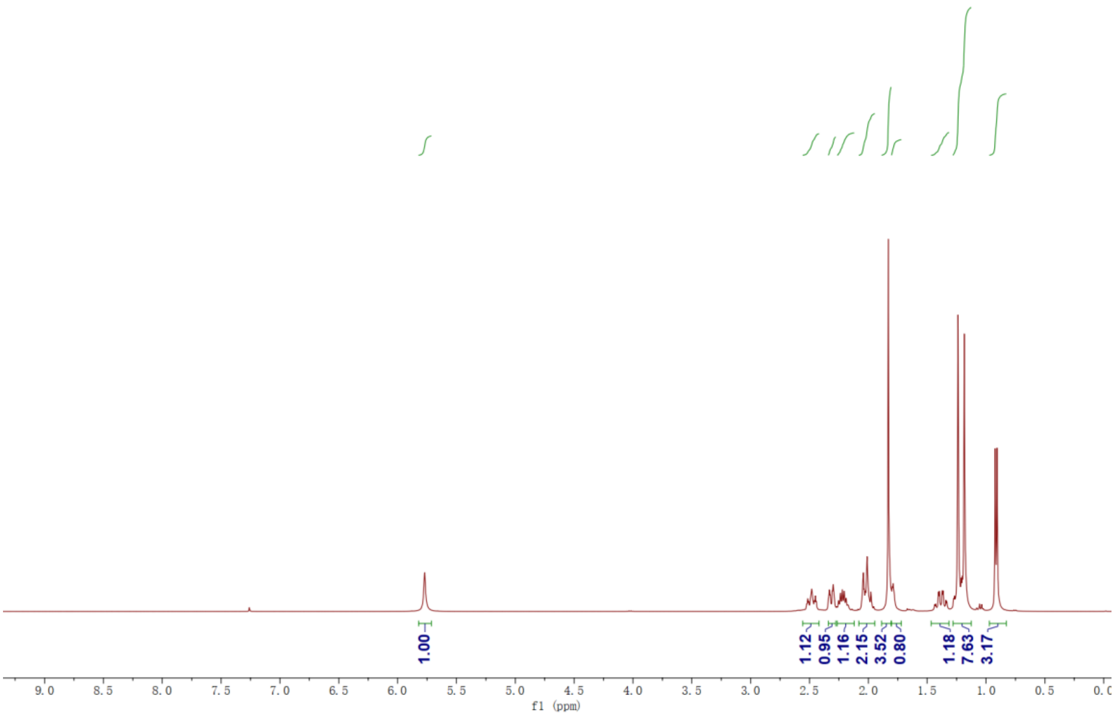

Supplementary Figure 50. ^{1}H NMR spectrum of 3r

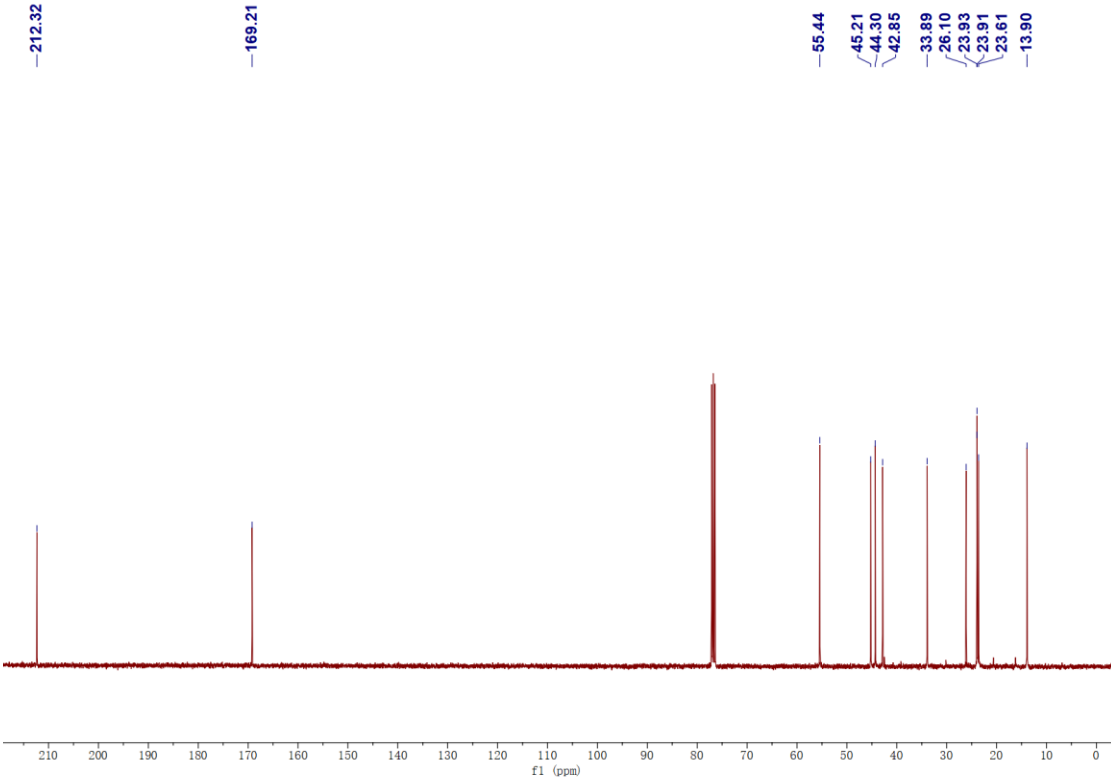

Supplementary Figure 51. ^{13}C NMR spectrum of 3r

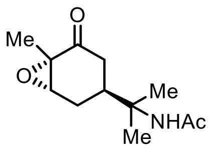

3s

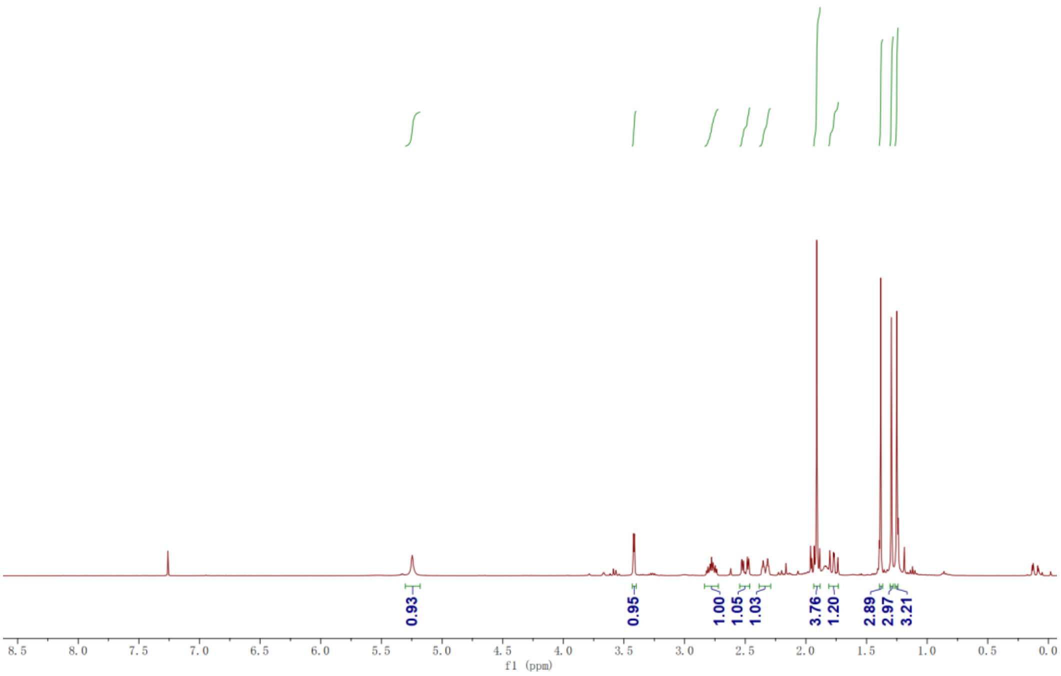

Supplementary Figure 52. ^{1}H NMR spectrum of 3s

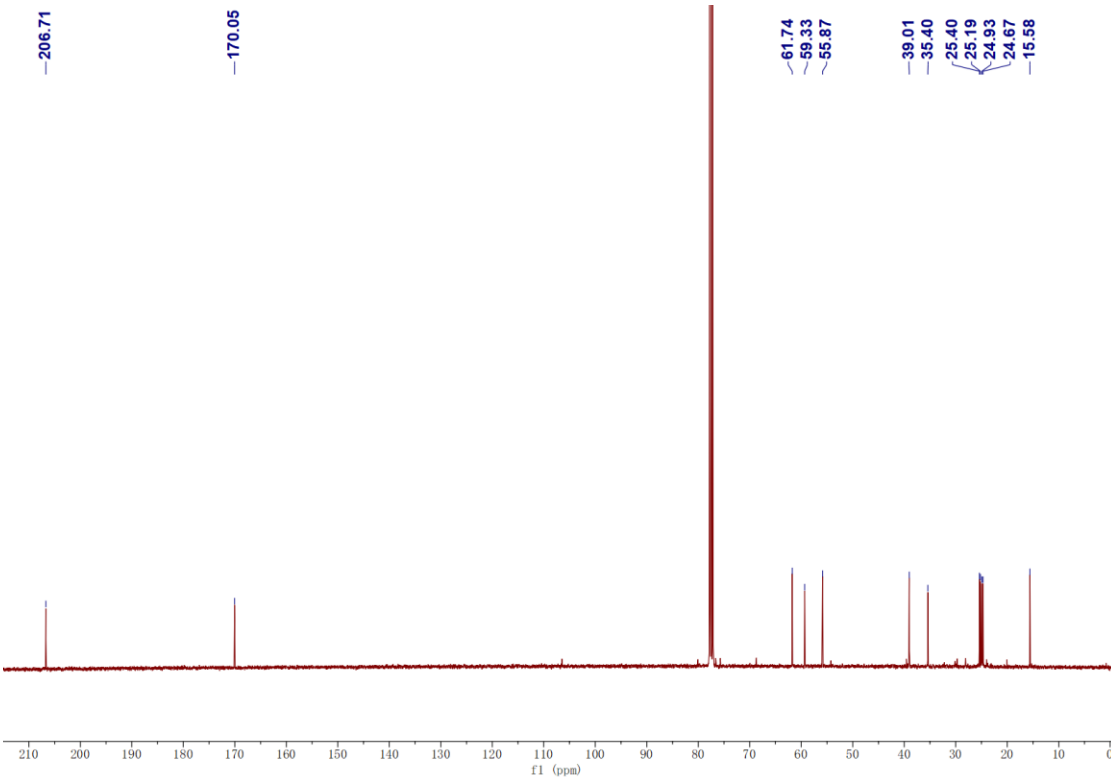

Supplementary Figure 53. ^{13}C NMR spectrum of 3s

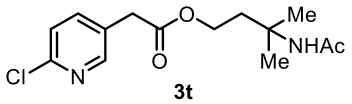

3t

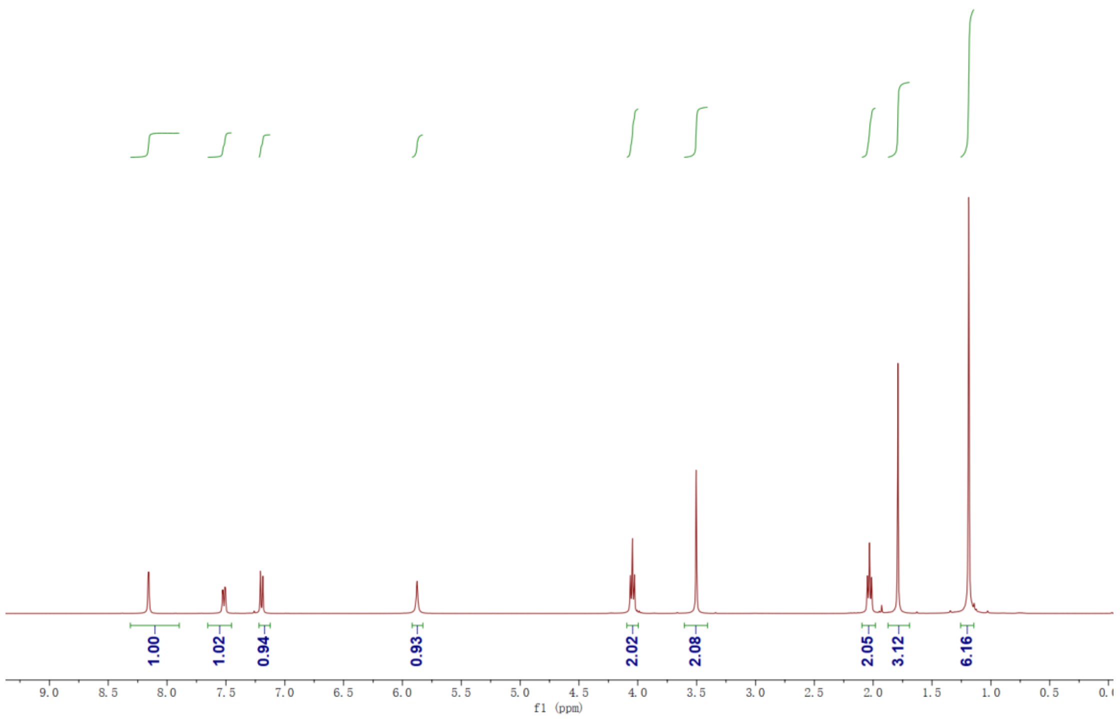

Supplementary Figure 54. ^{1}H NMR spectrum of 3t

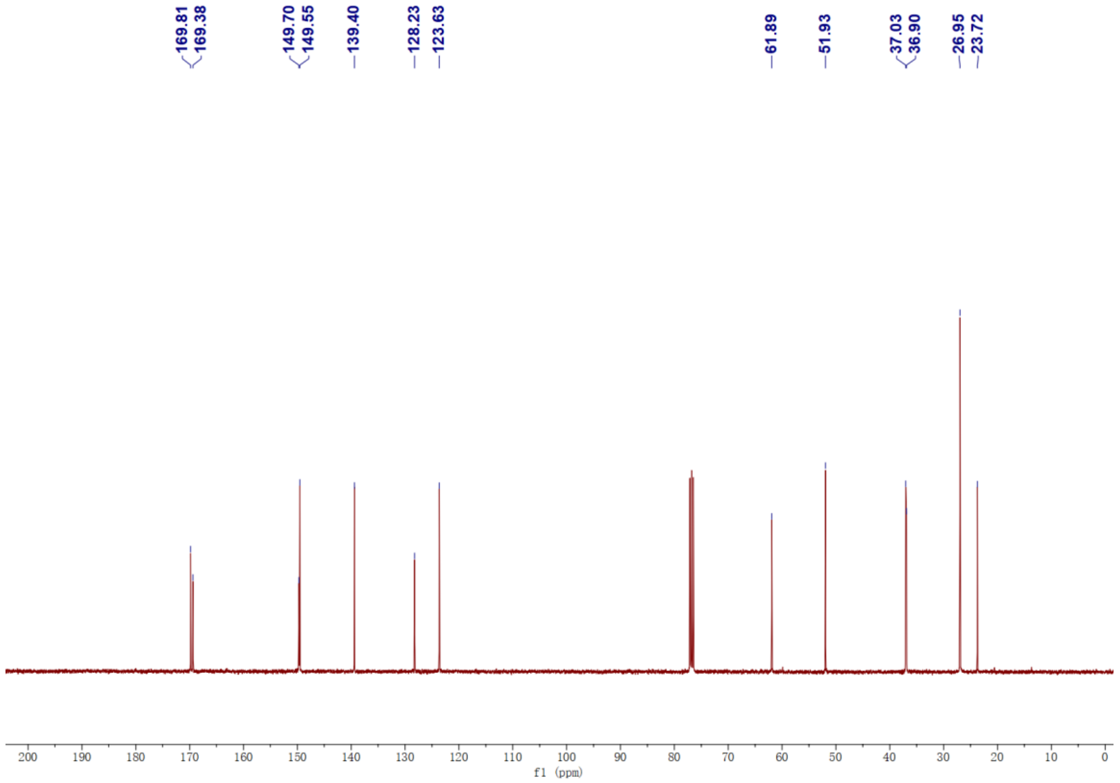

Supplementary Figure 55. ^{13}C NMR spectrum of 3t

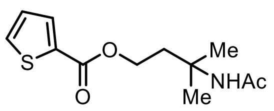

3u

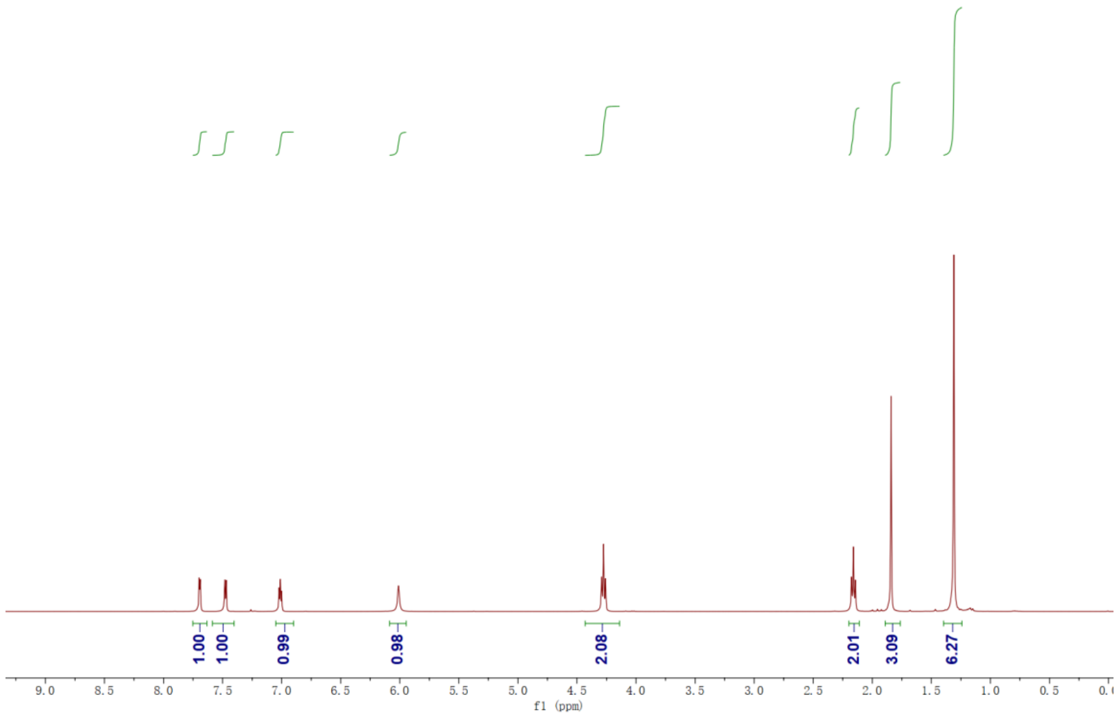

Supplementary Figure 56. ^{1}H NMR spectrum of 3u

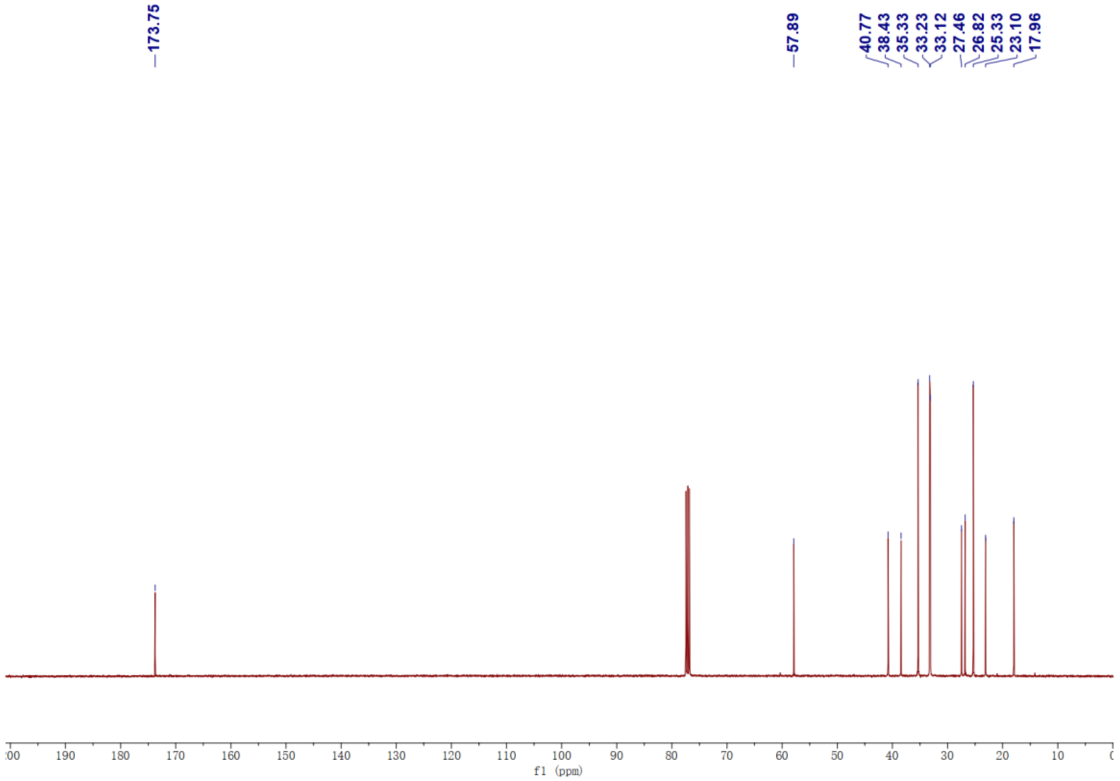

Supplementary Figure 57. ^{13}C NMR spectrum of 3u

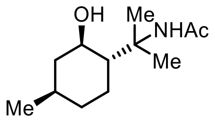

3v

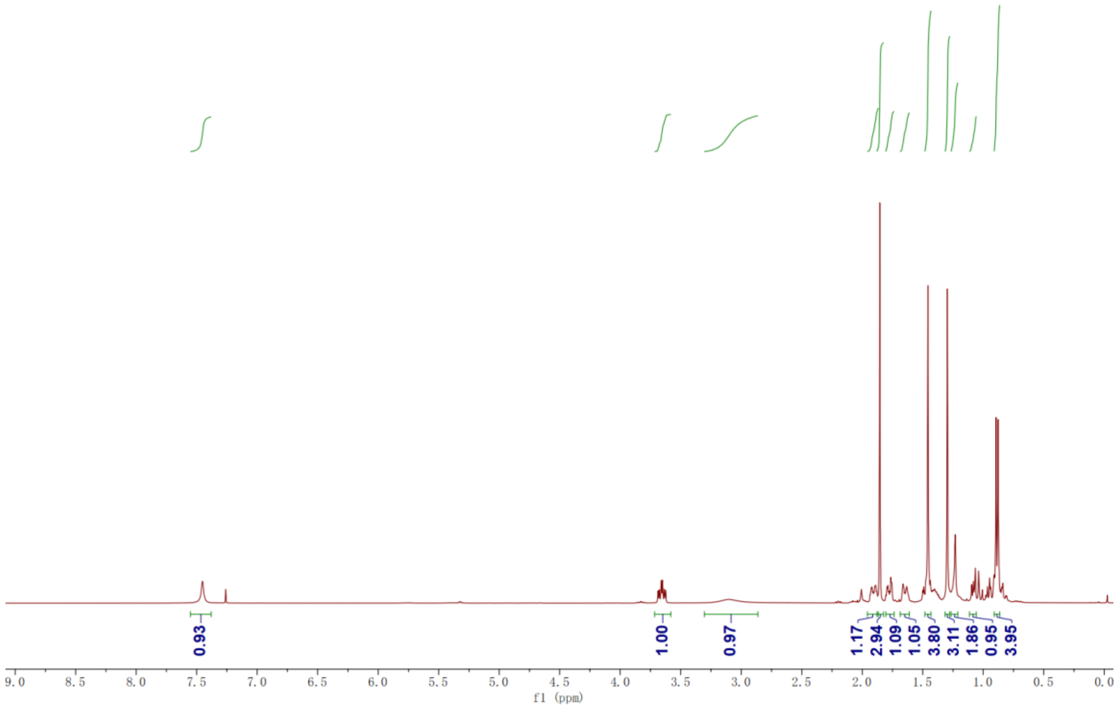

Supplementary Figure 58. ^{1}H NMR spectrum of 3v

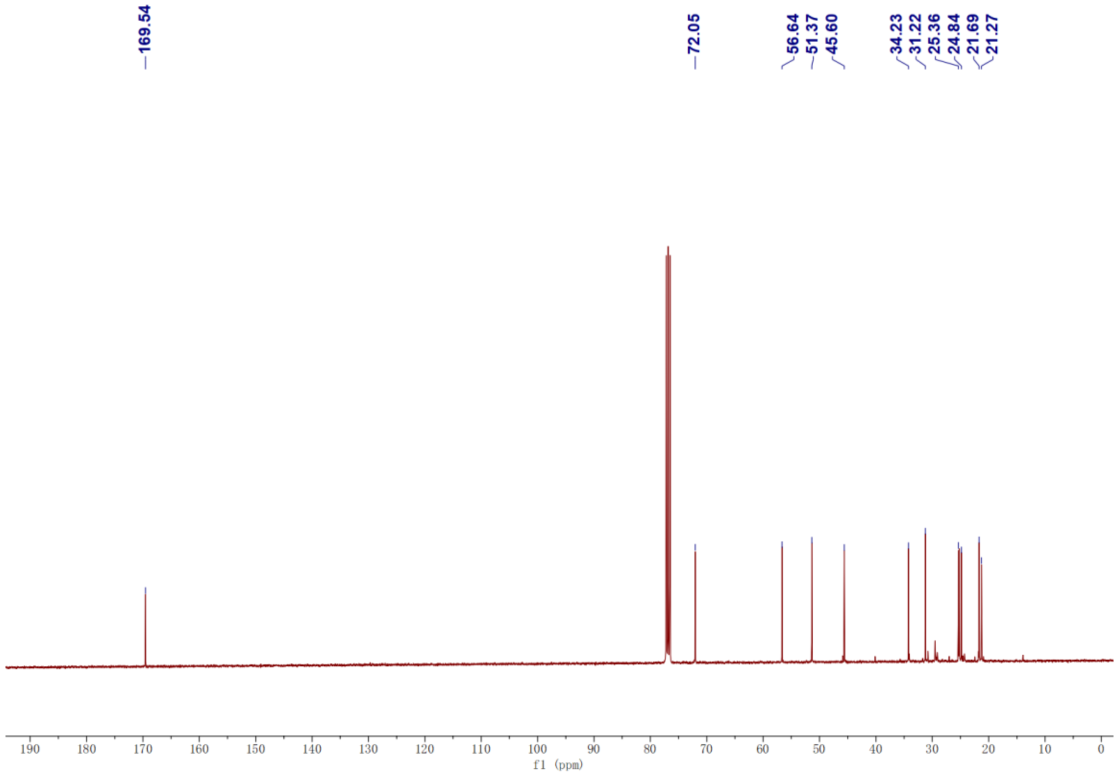

Supplementary Figure 59. ^{13}C NMR spectrum of 3v

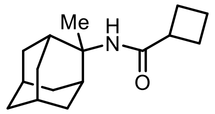

7a

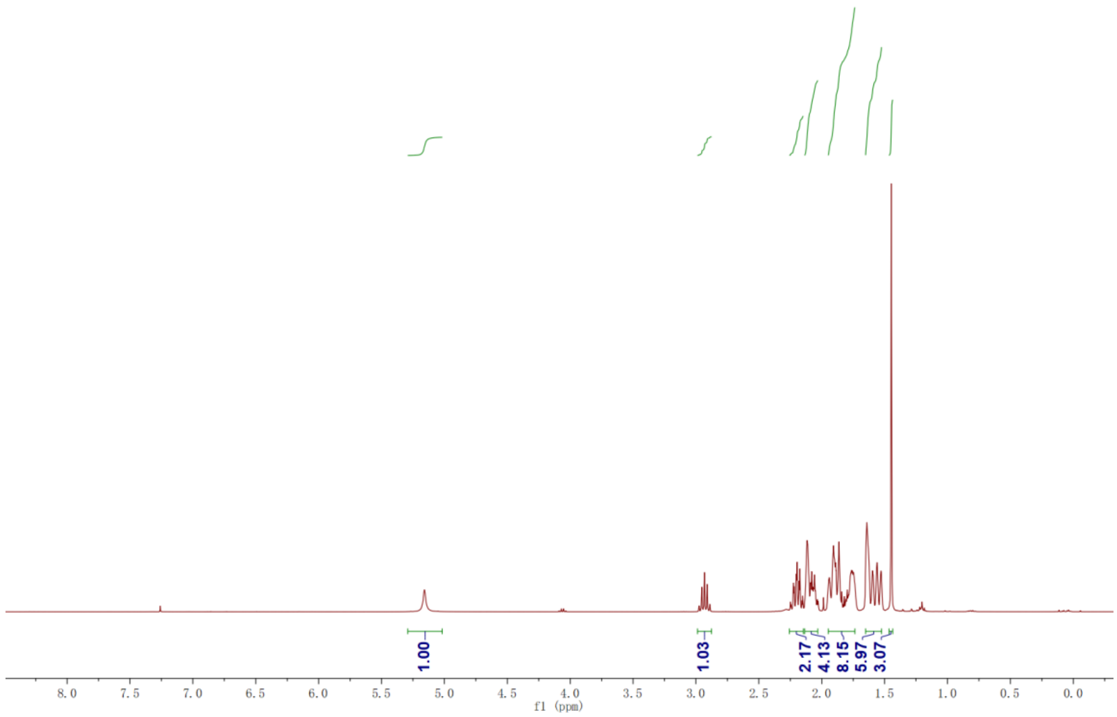

Supplementary Figure 60. ^{1}**H NMR spectrum of 7a**

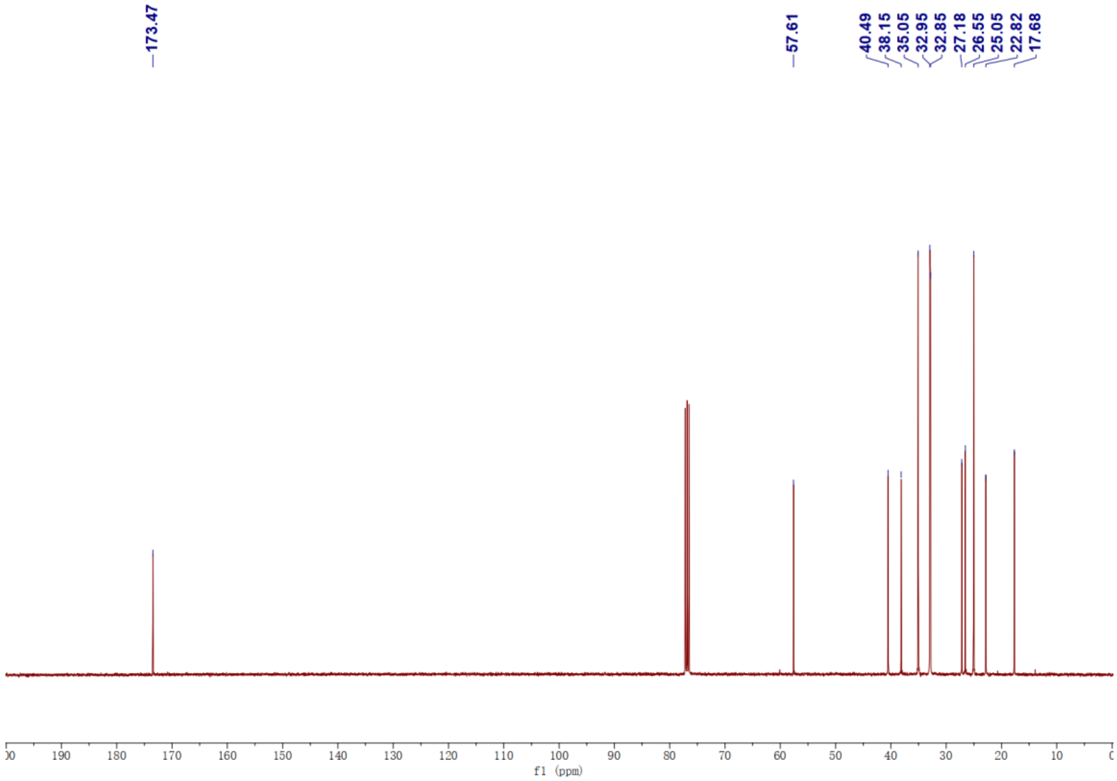

Supplementary Figure 61. ^{13}**C NMR spectrum of 7a**

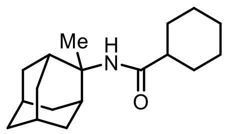

7b

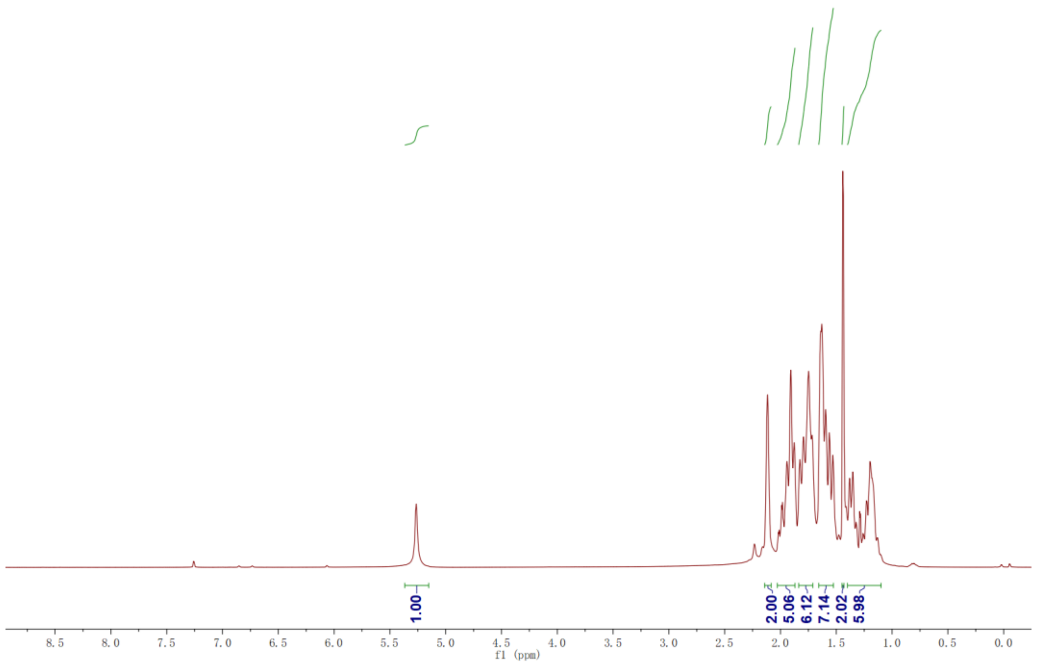

Supplementary Figure 62. ^{1}H NMR spectrum of 7b

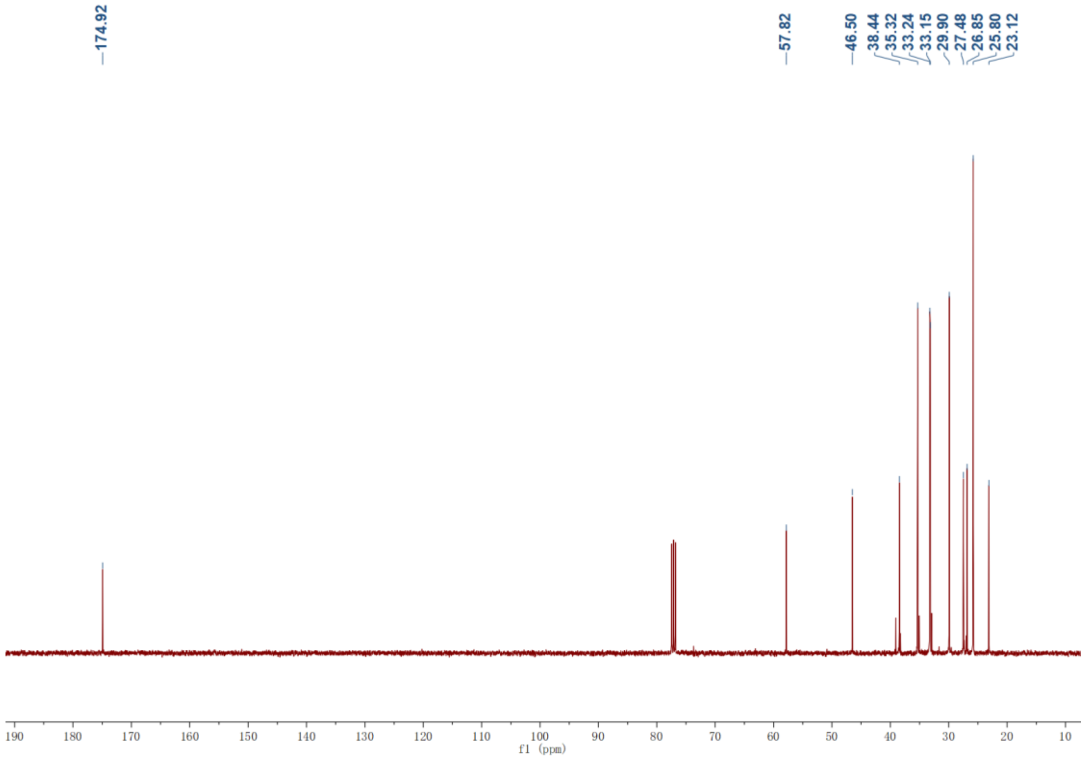

Supplementary Figure 63. ^{13}C NMR spectrum of 7b

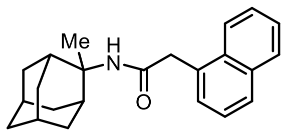

7c

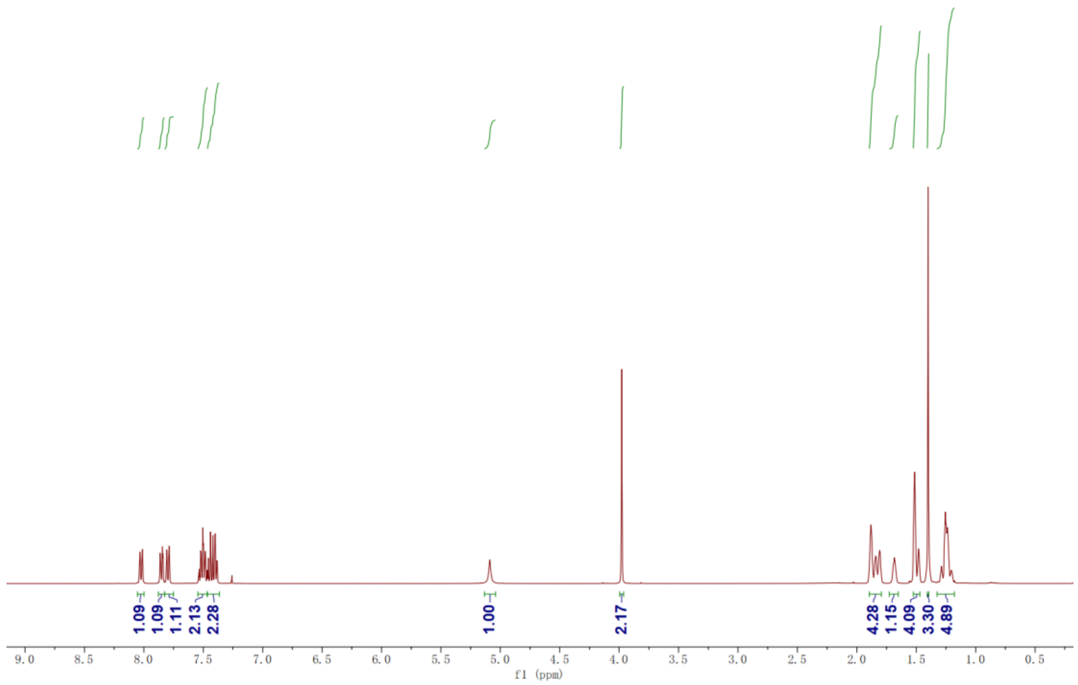

Supplementary Figure 64. ¹H NMR spectrum of 7c

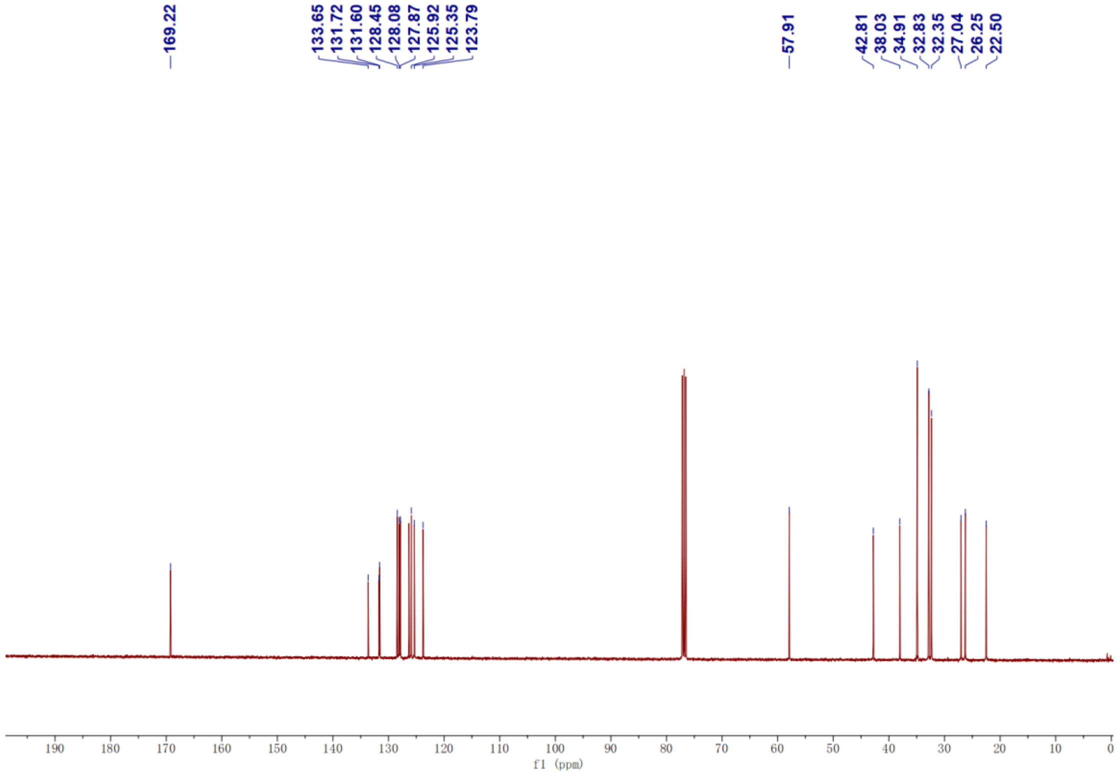

Supplementary Figure 65. ¹³C NMR spectrum of 7c

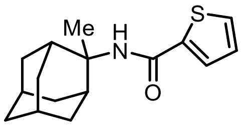

7d

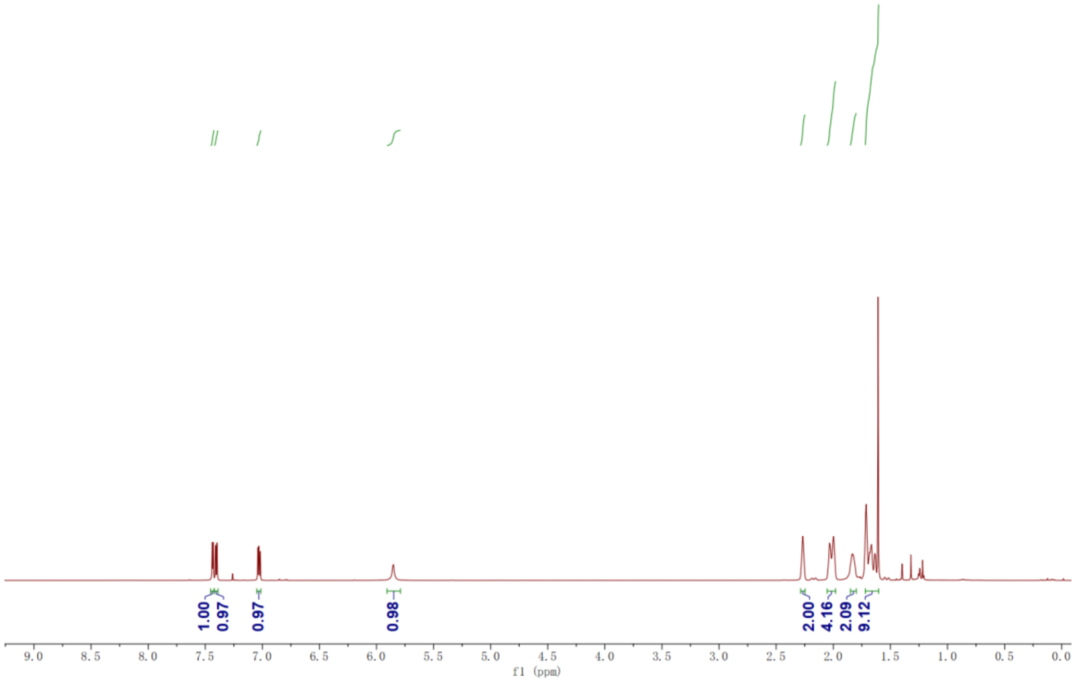

Supplementary Figure 66. ^{1}H NMR spectrum of 7d

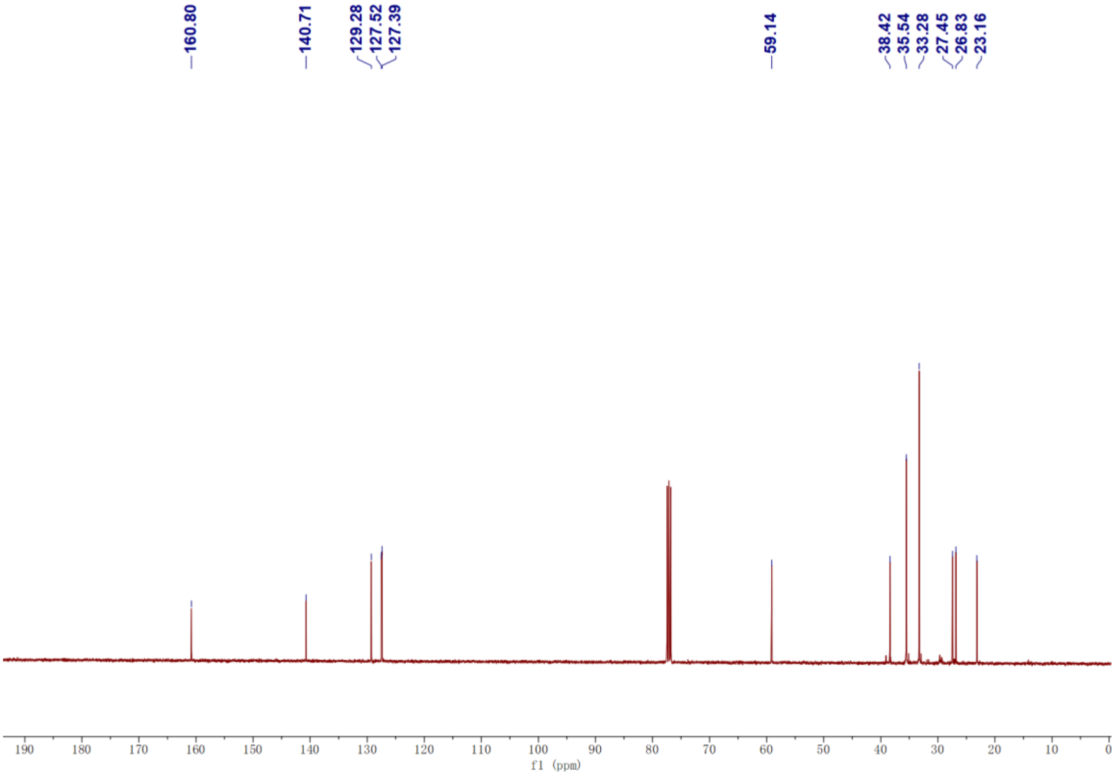

Supplementary Figure 67. ^{13}C NMR spectrum of 7d

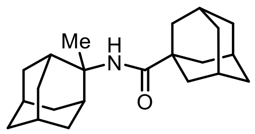

7e

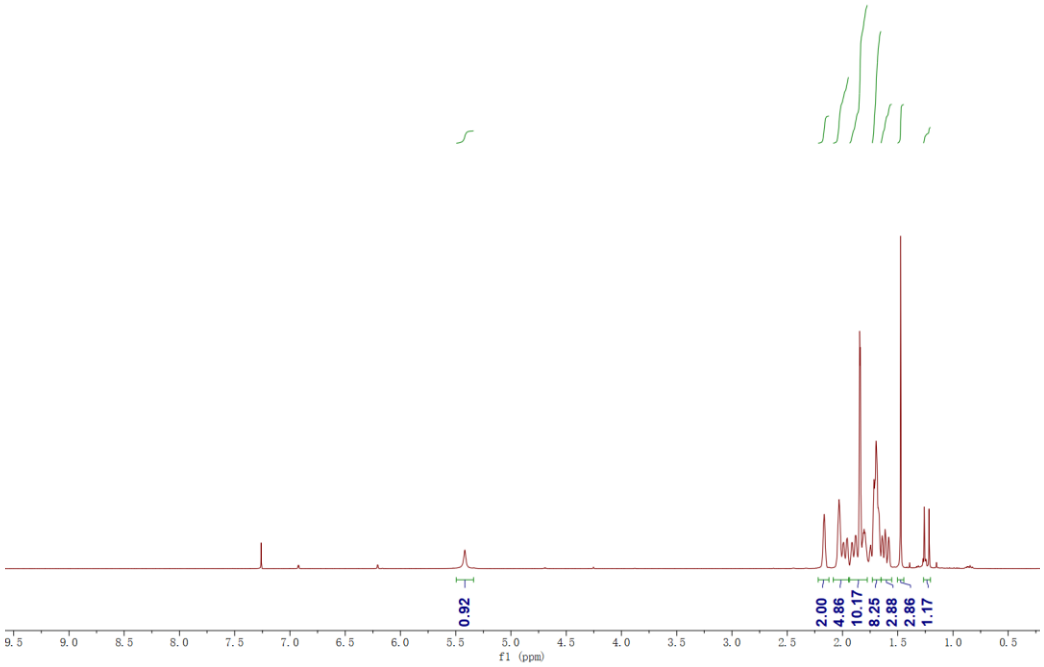

Supplementary Figure 68. ^{1}H NMR spectrum of 7e

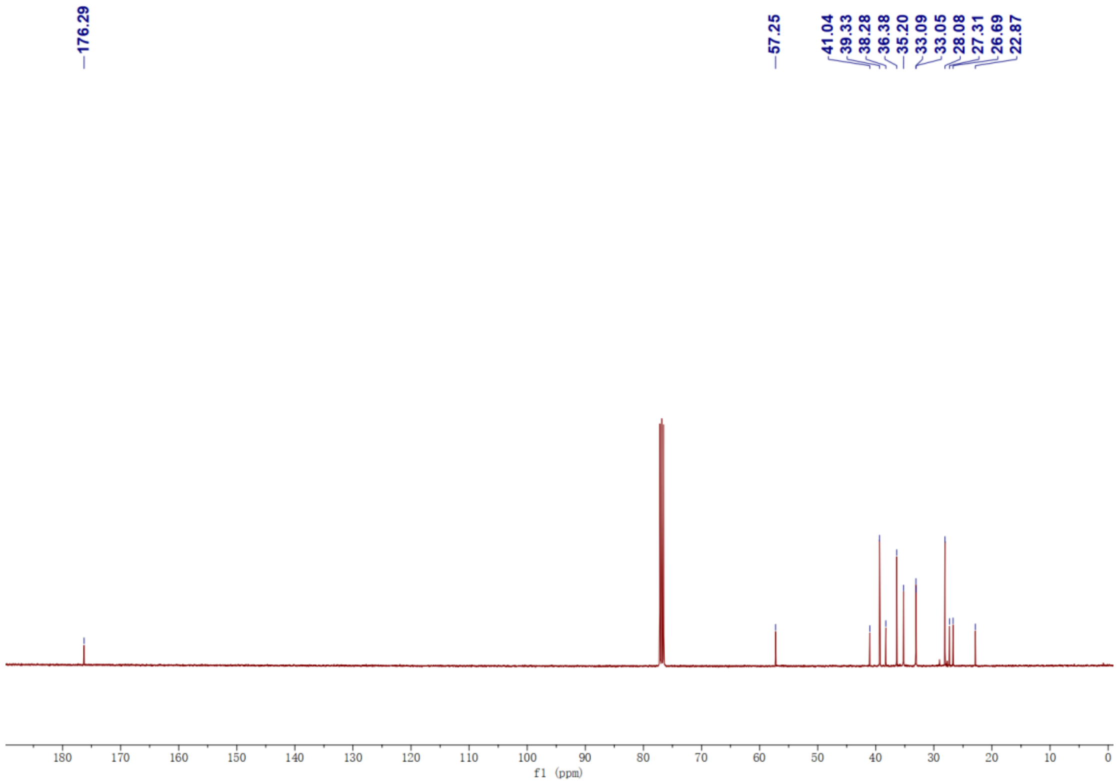

Supplementary Figure 69. ^{13}C NMR spectrum of 7e

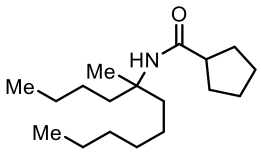

7f

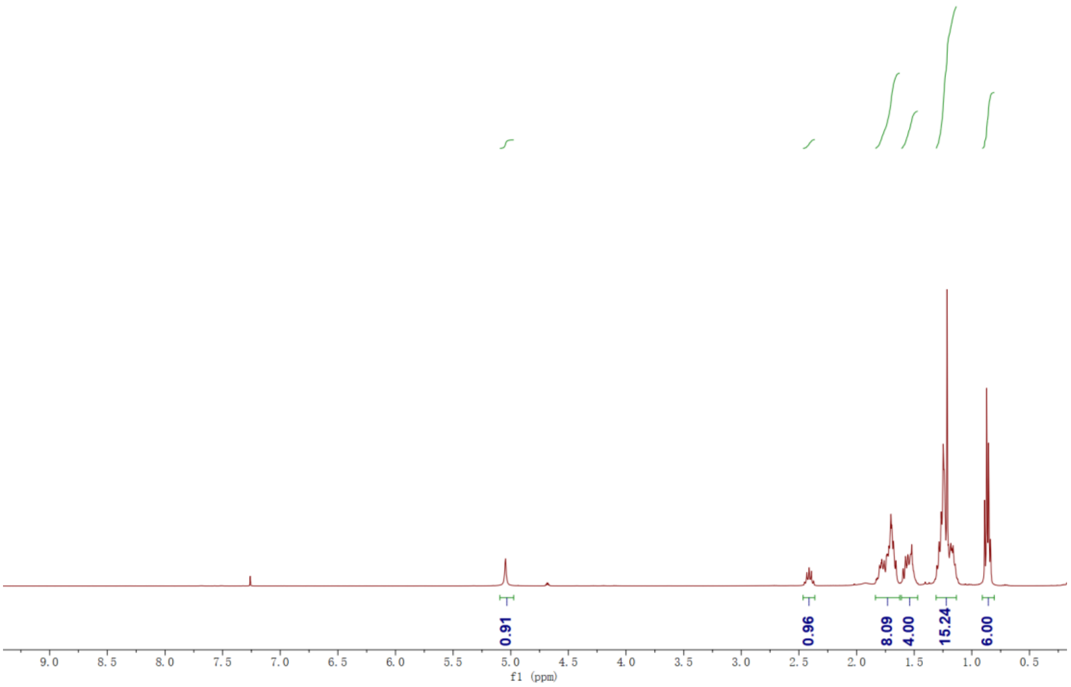

Supplementary Figure 70. ^{1}H NMR spectrum of 7f

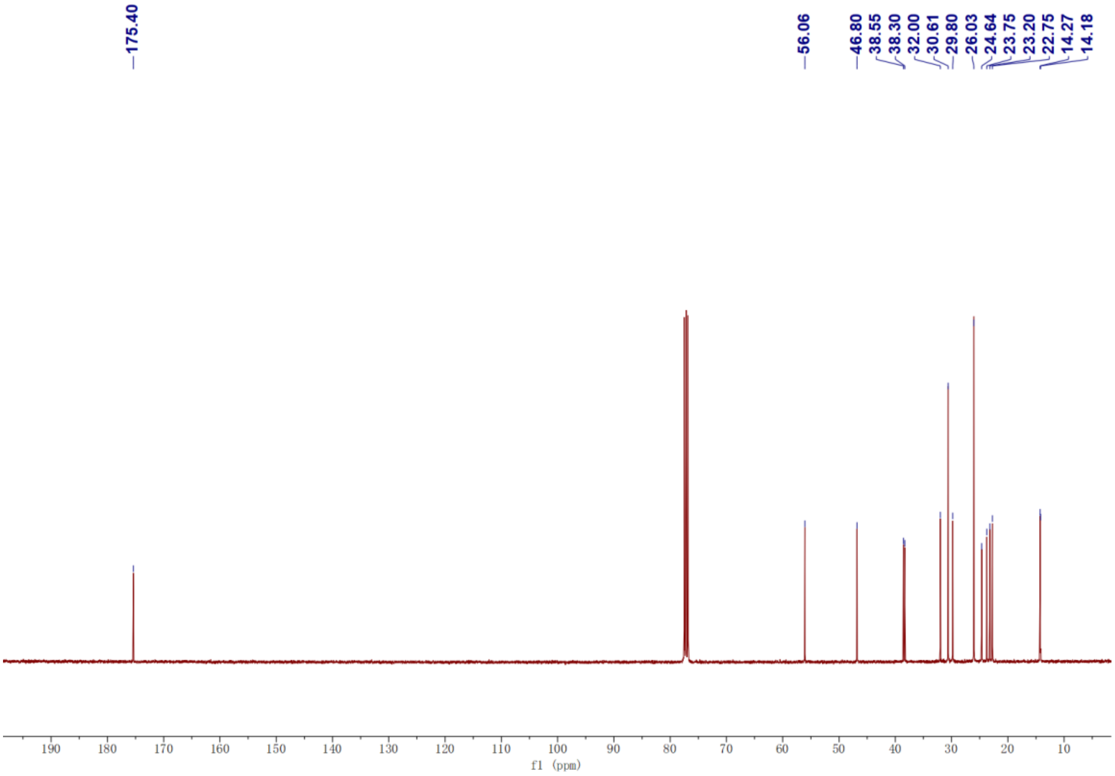

Supplementary Figure 71. ^{13}C NMR spectrum of 7f

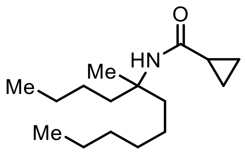

7g

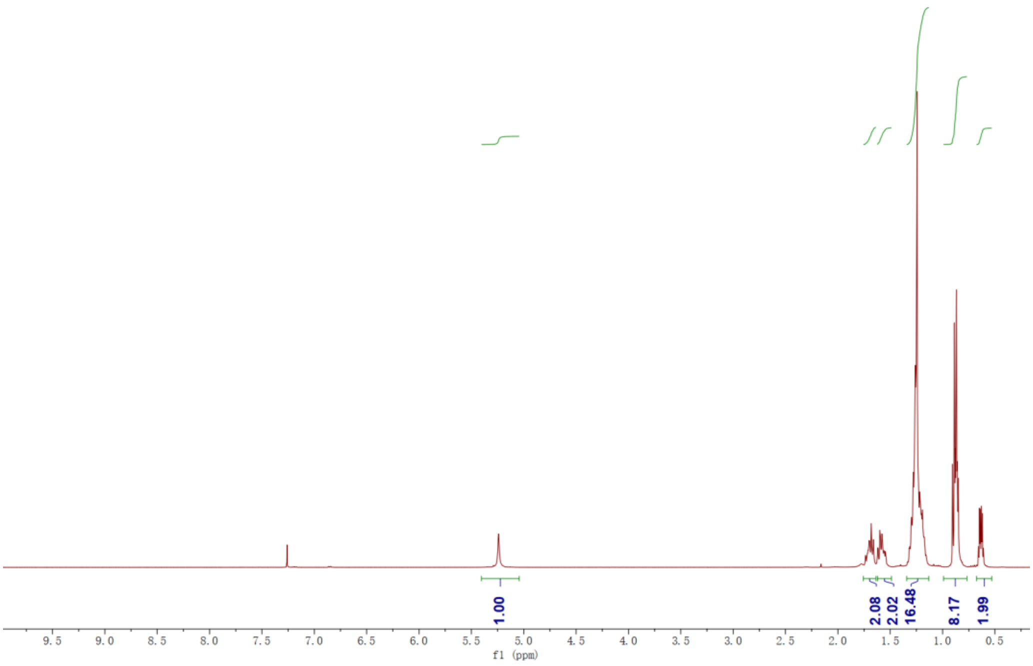

Supplementary Figure 72. ^{1}H NMR spectrum of 7g

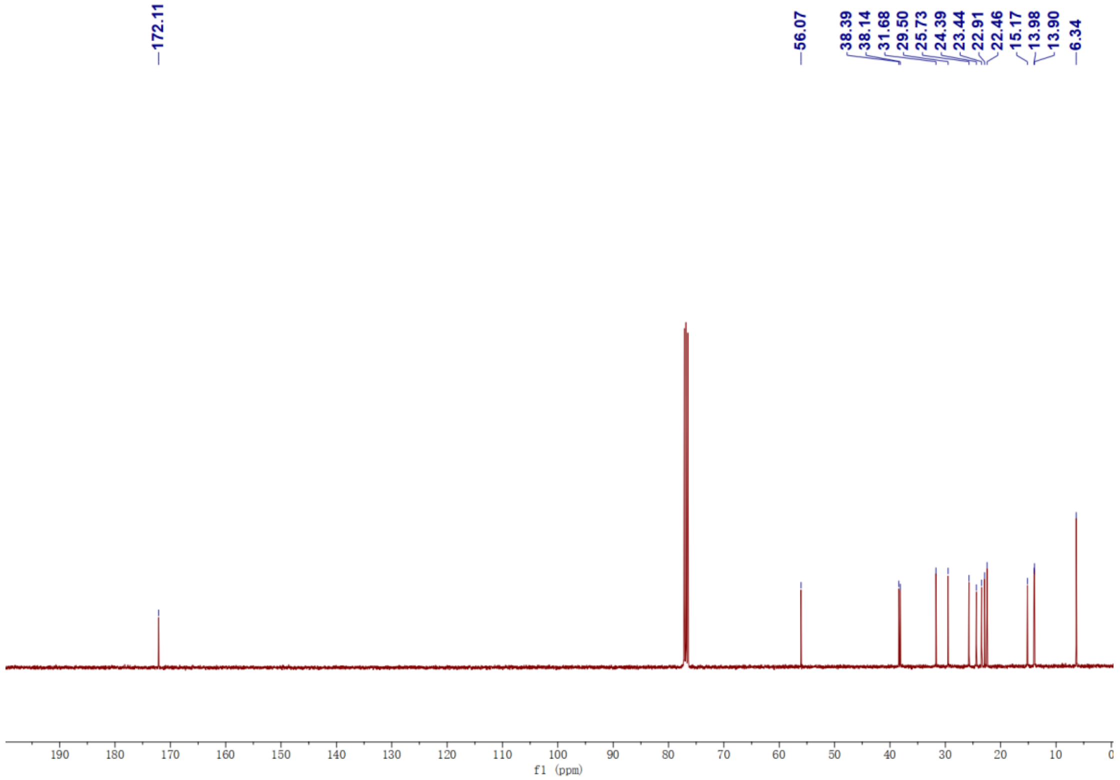

Supplementary Figure 73. ^{13}C NMR spectrum of 7g

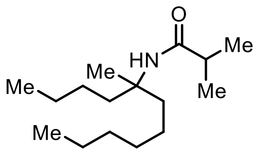

7h

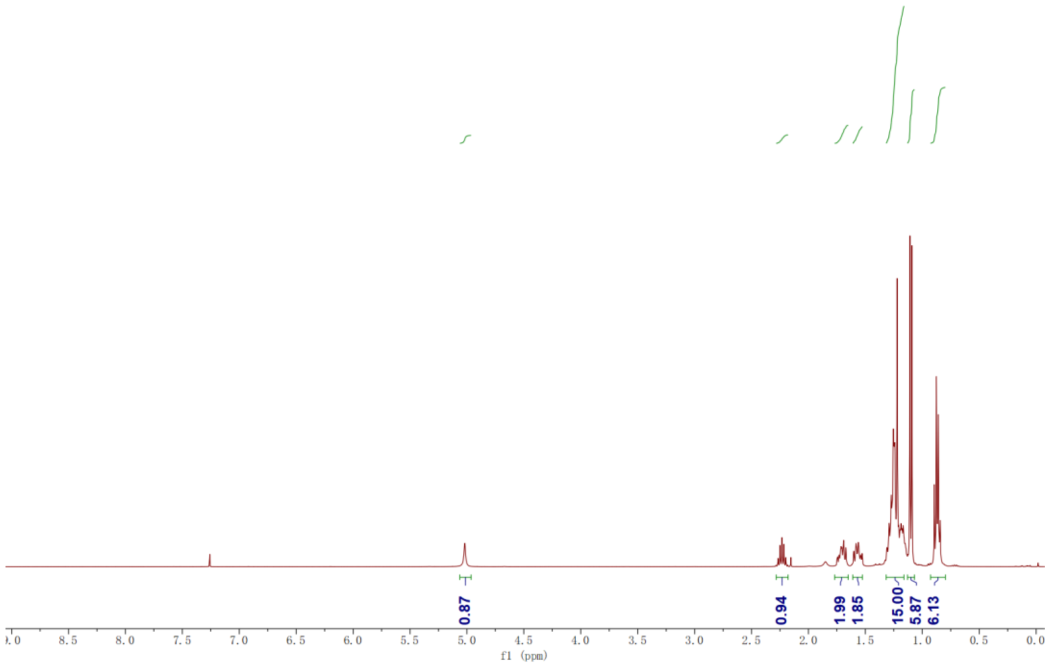

Supplementary Figure 74. ^{1}H NMR spectrum of 7h

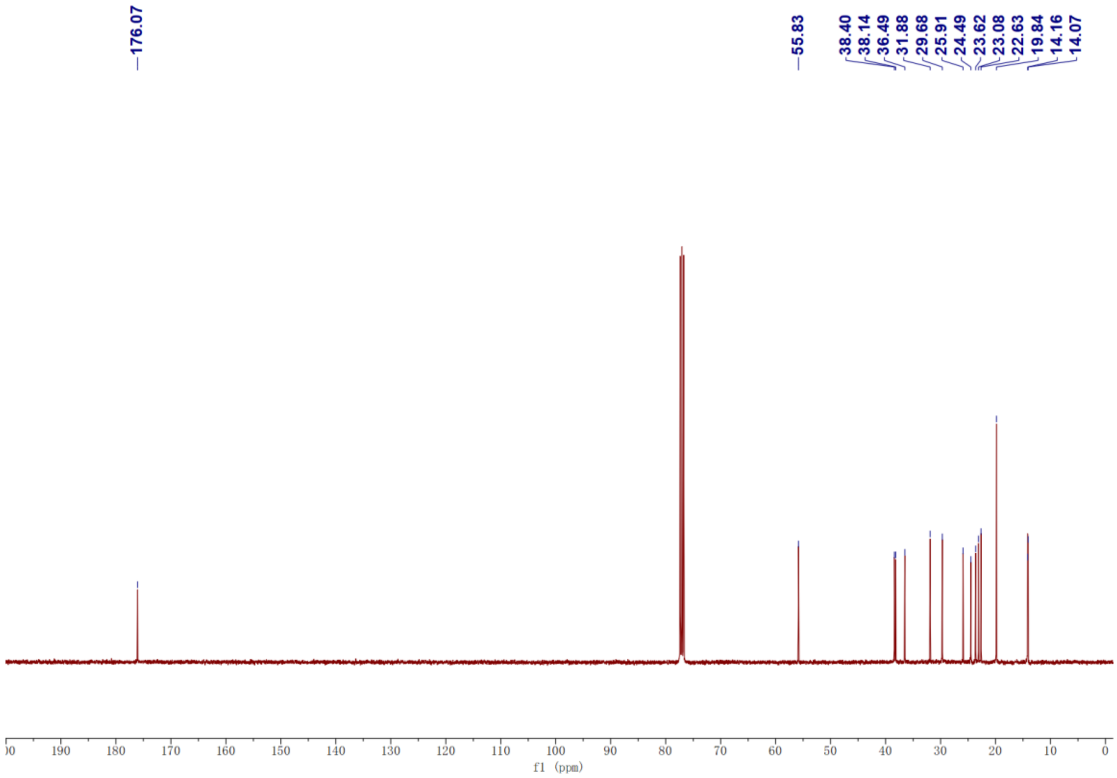

Supplementary Figure 75. ^{13}C NMR spectrum of 7h

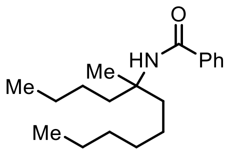

7i

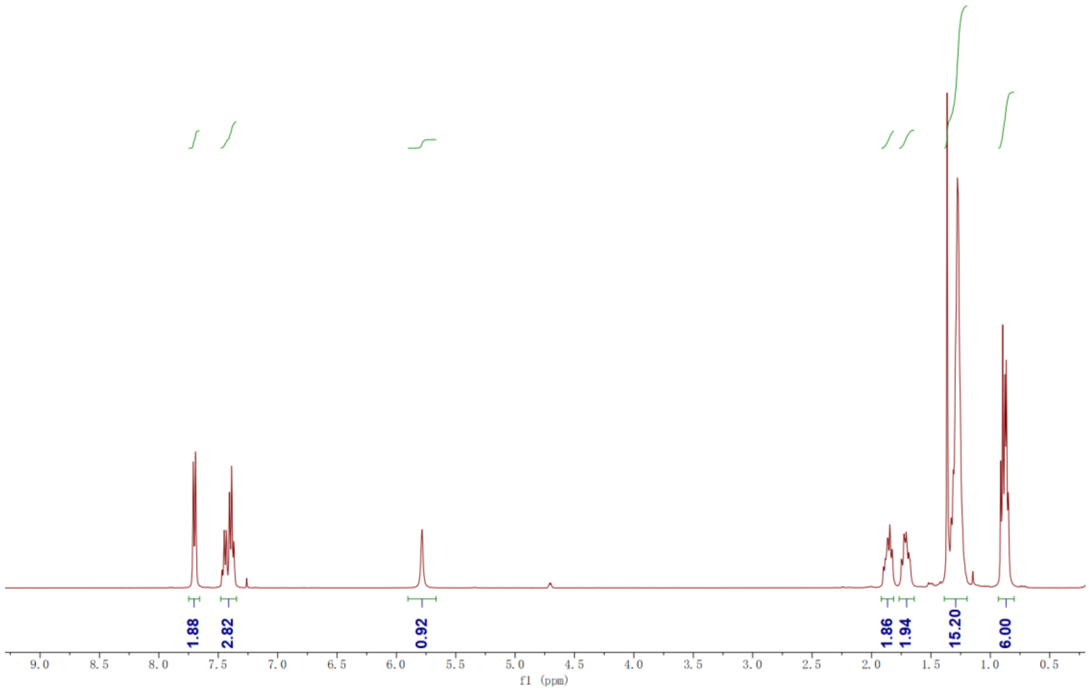

Supplementary Figure 76. ^{1}**H NMR spectrum of 7i**

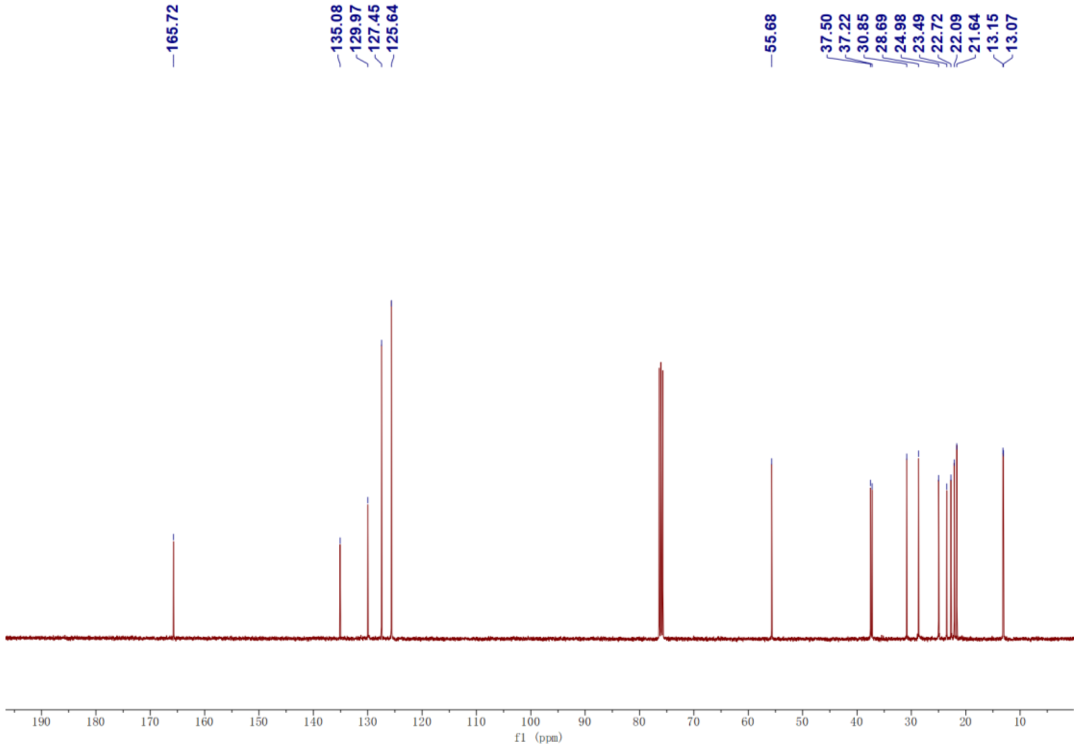

Supplementary Figure 77. ^{13}**C NMR spectrum of 7i**

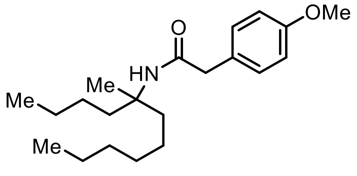

7j

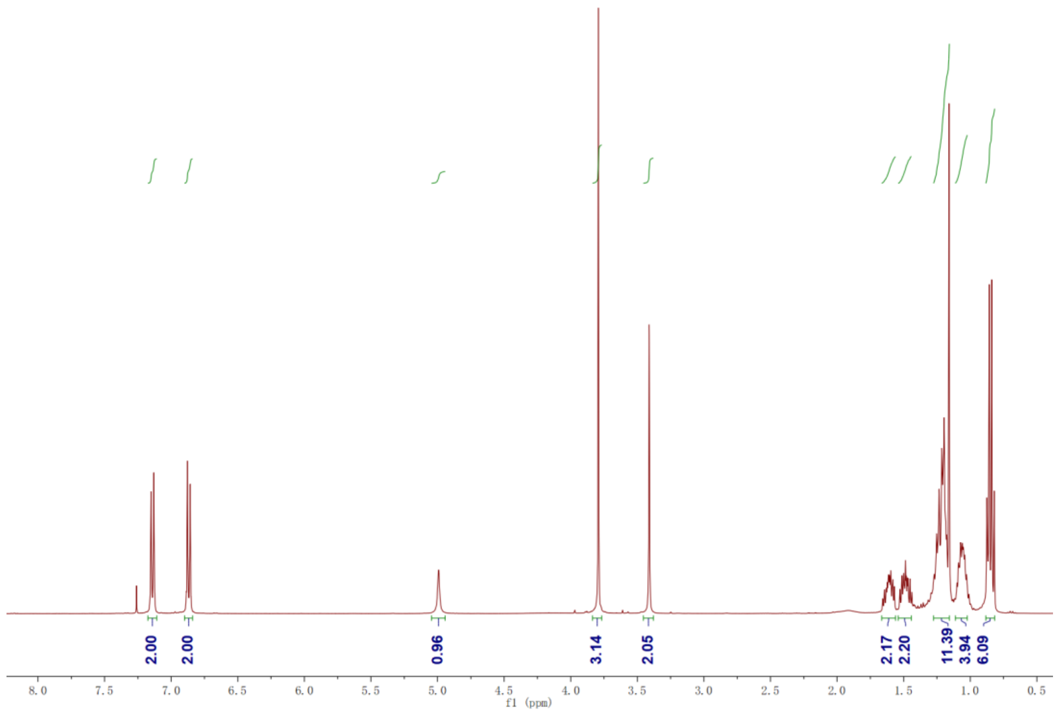

Supplementary Figure 78. ^{1}H NMR spectrum of 7j

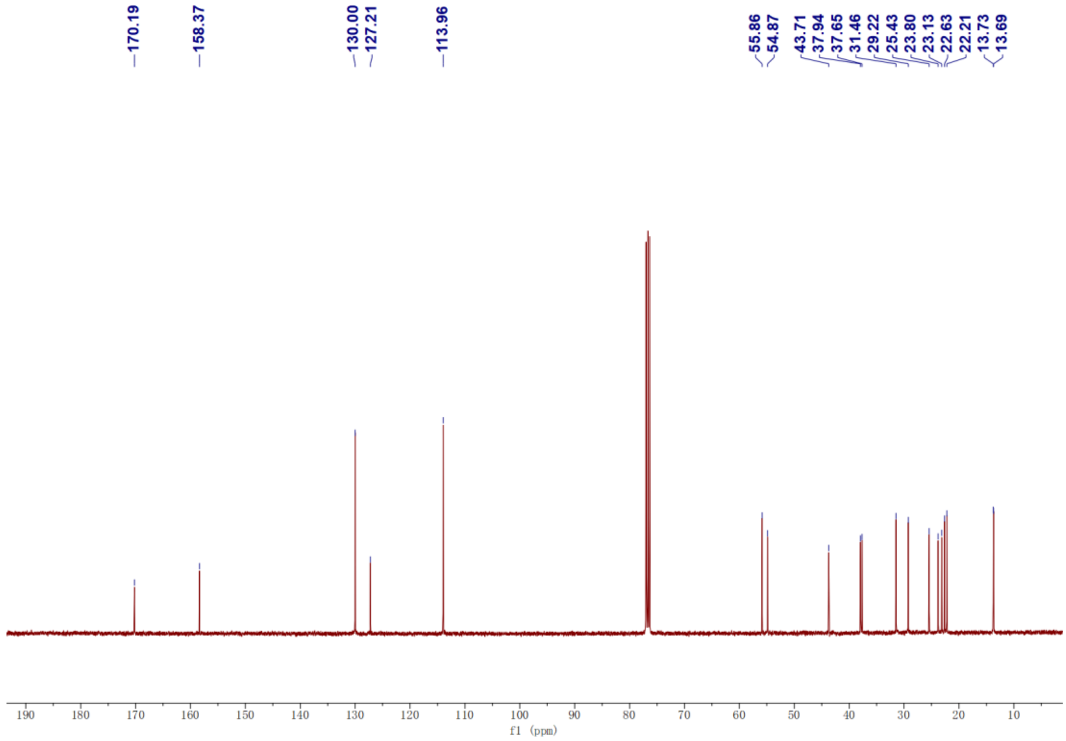

Supplementary Figure 79. ^{13}C NMR spectrum of 7j

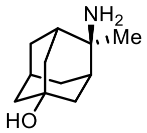

8

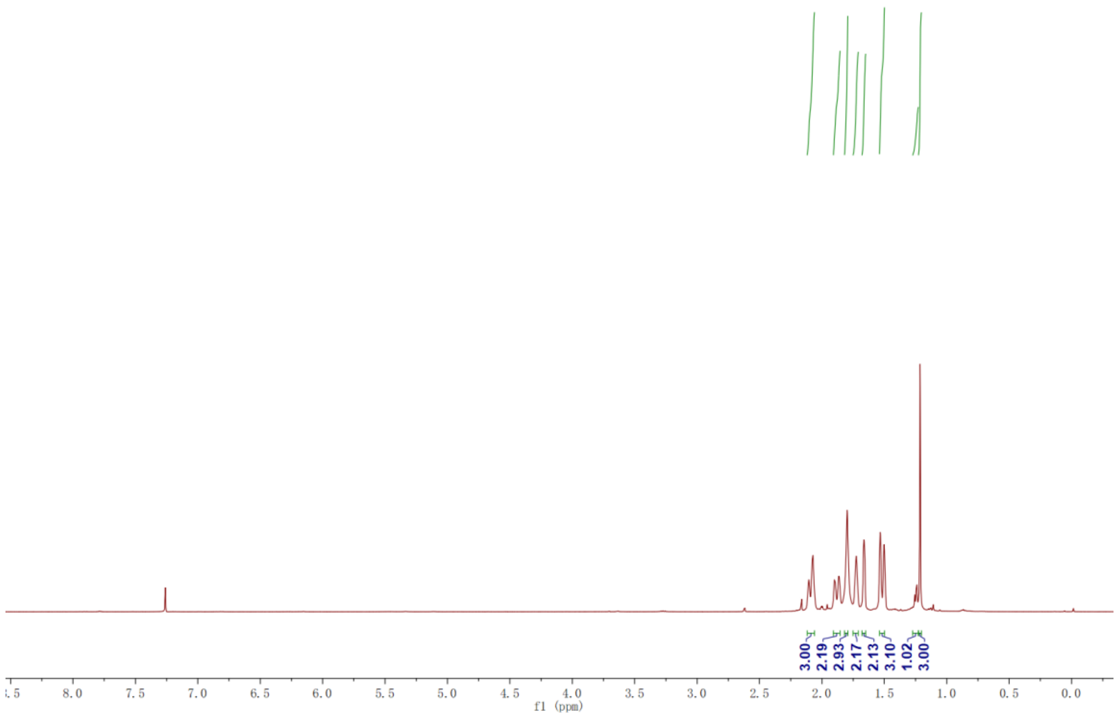

Supplementary Figure 80. ^{1}H NMR spectrum of 8

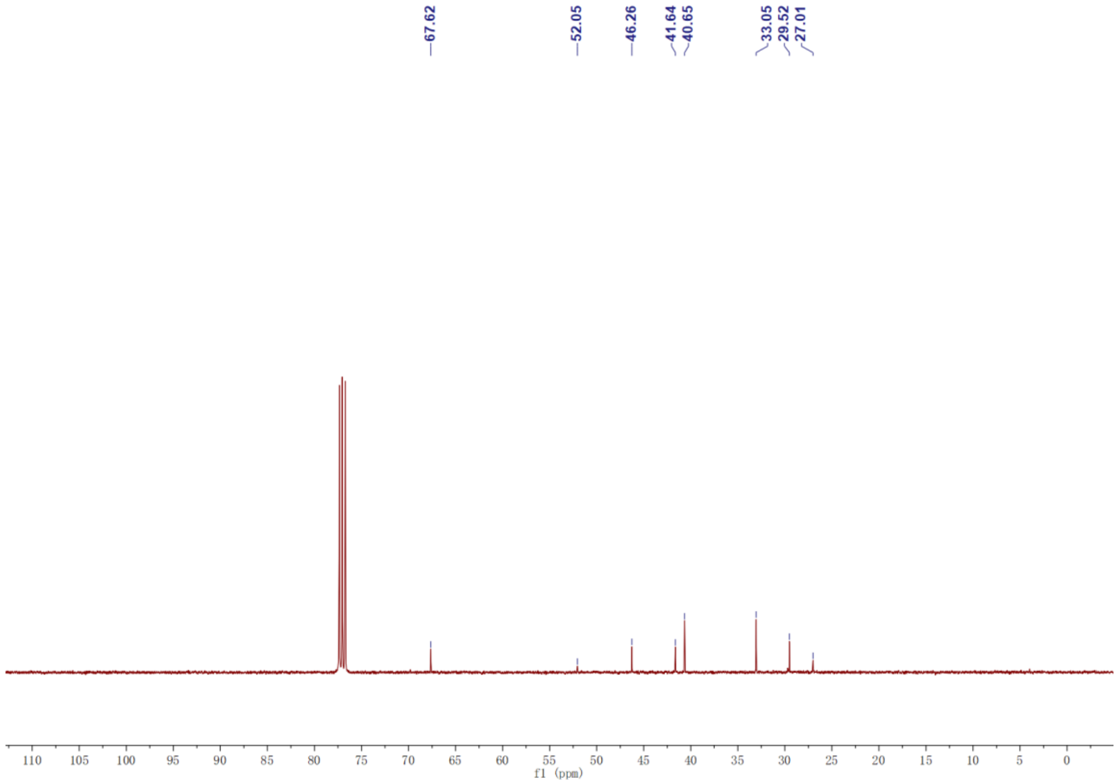

Supplementary Figure 81. ^{13}C NMR spectrum of 8

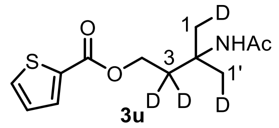

3u

$C_{1/1'}$: 127%

C_3: 0%

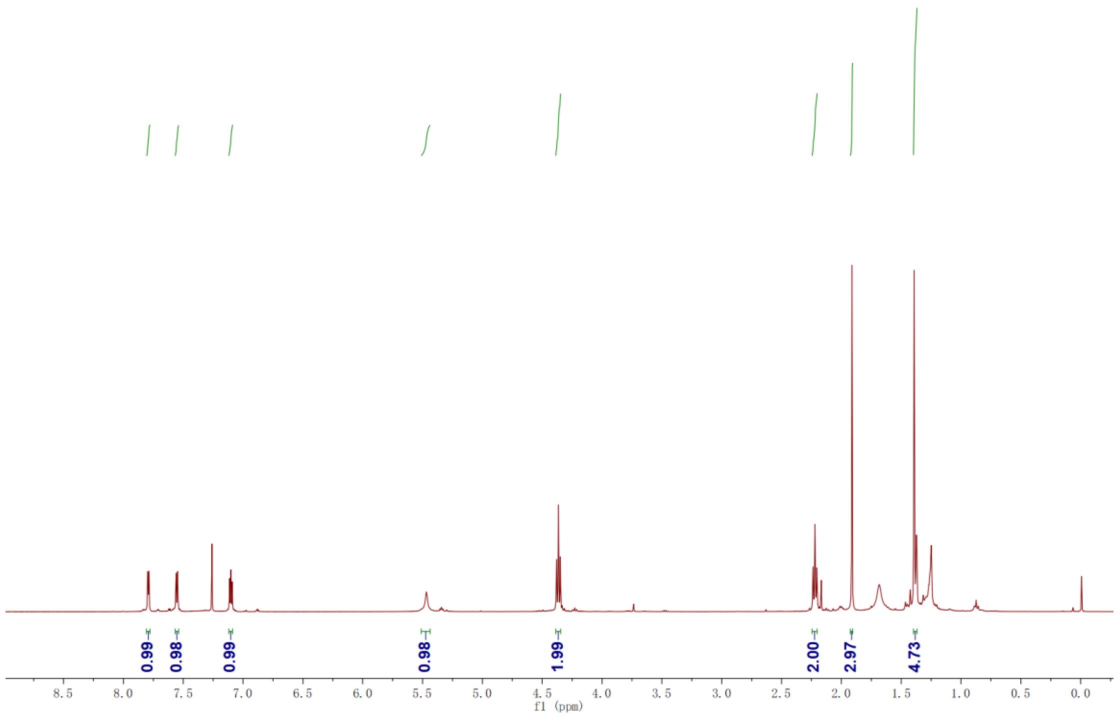

Supplementary Figure 82. ^{1}H NMR spectrum of 3u

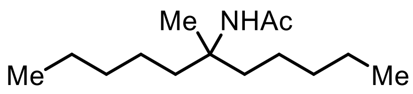

YYN-2-65-2, 3w, in the presence of D_2O

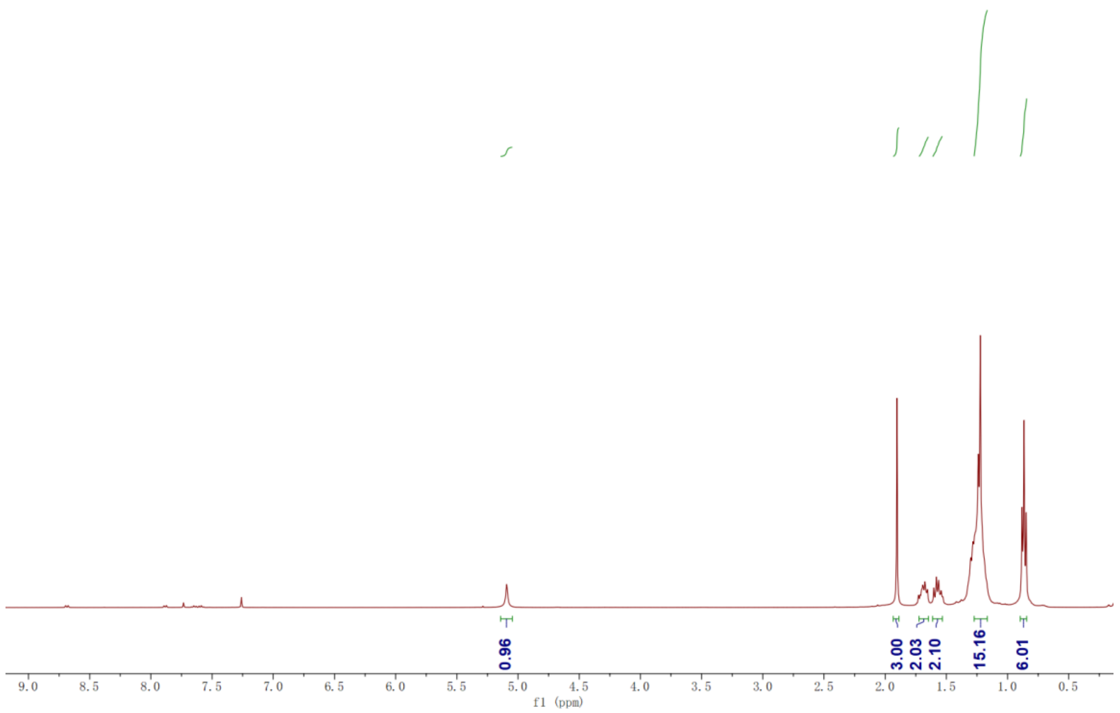

Supplementary Figure 83. ^{1}H NMR spectrum of 3w

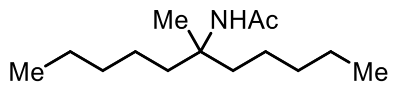

YYN-2-65-1, 3w, in the presence of H_2O

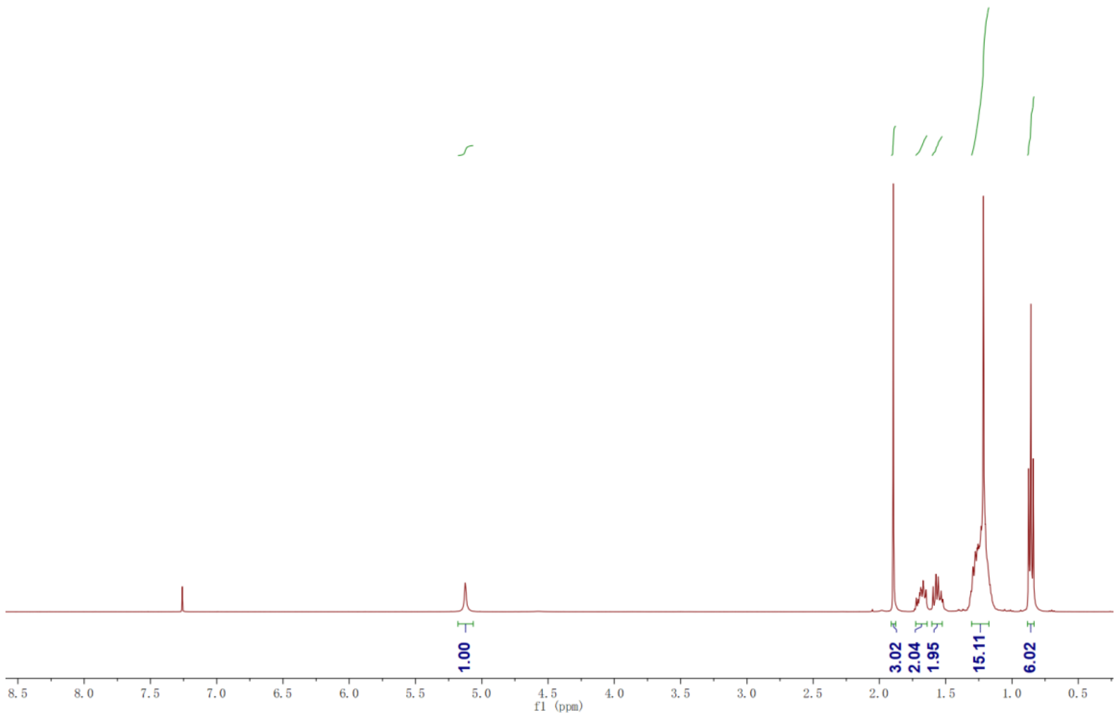

Supplementary Figure 84. ^{1}H NMR spectrum of 3w

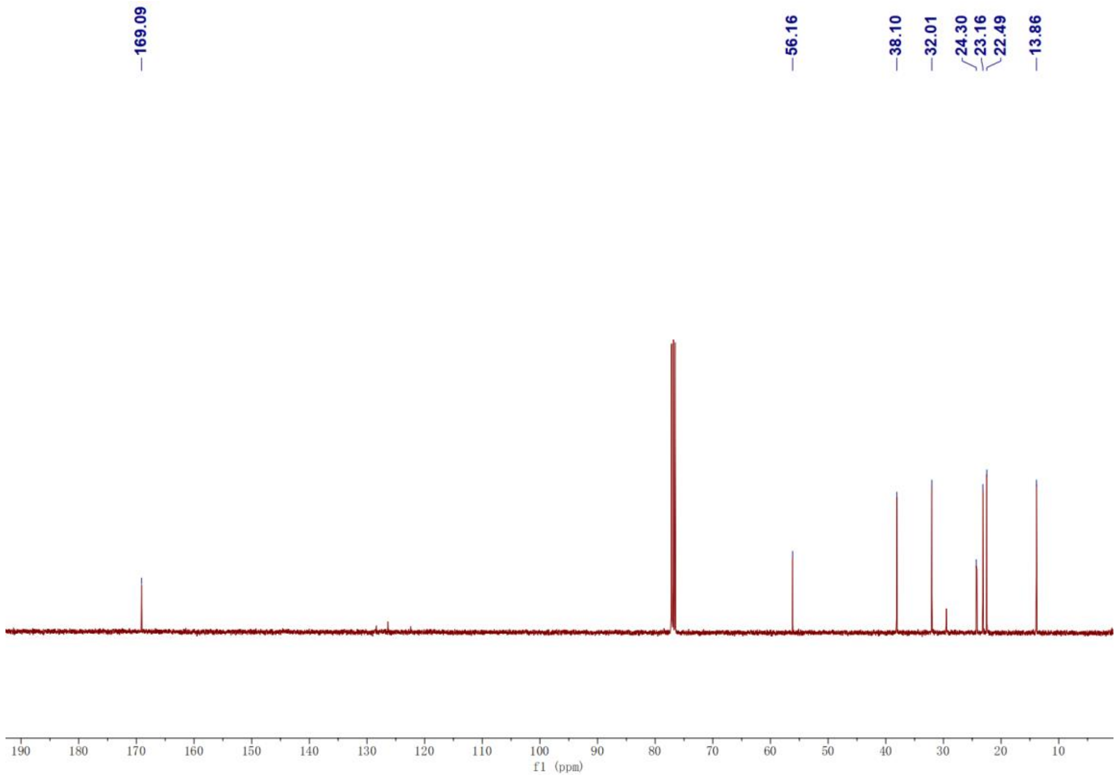

Supplementary Figure 85. ^{13}C NMR spectrum of 3w

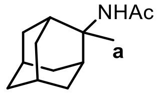

3c

a: 8% D

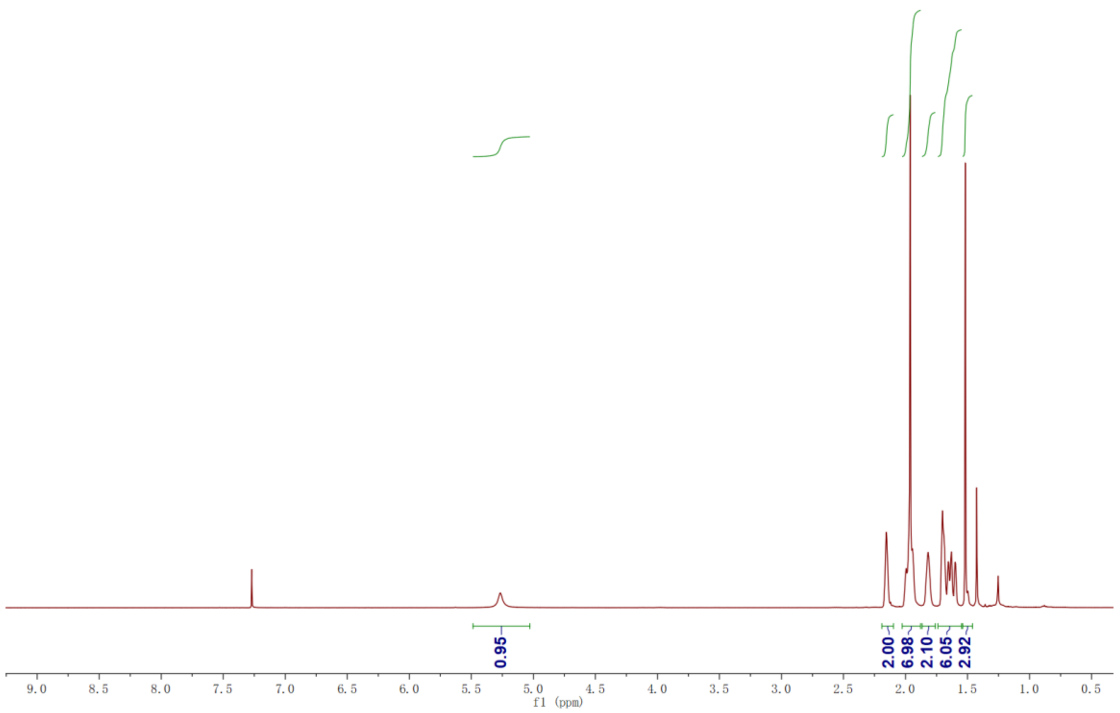

Supplementary Figure 86. ¹H NMR spectrum of 3c

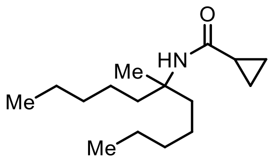

3w'

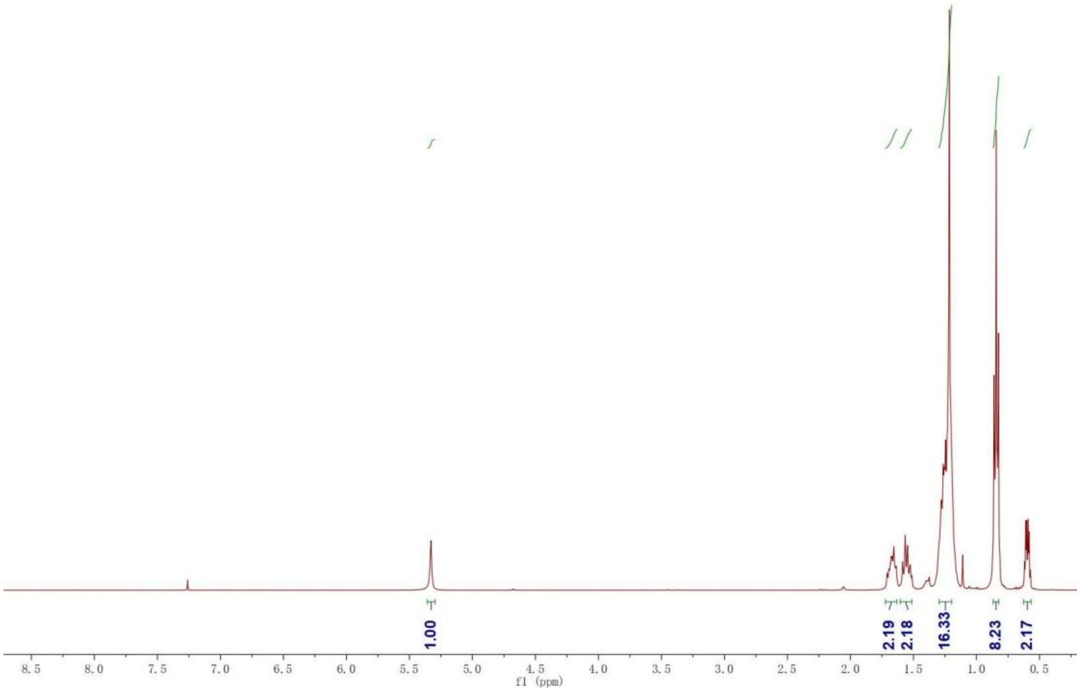

Supplementary Figure 87. ^{1}H NMR spectrum of 3w'

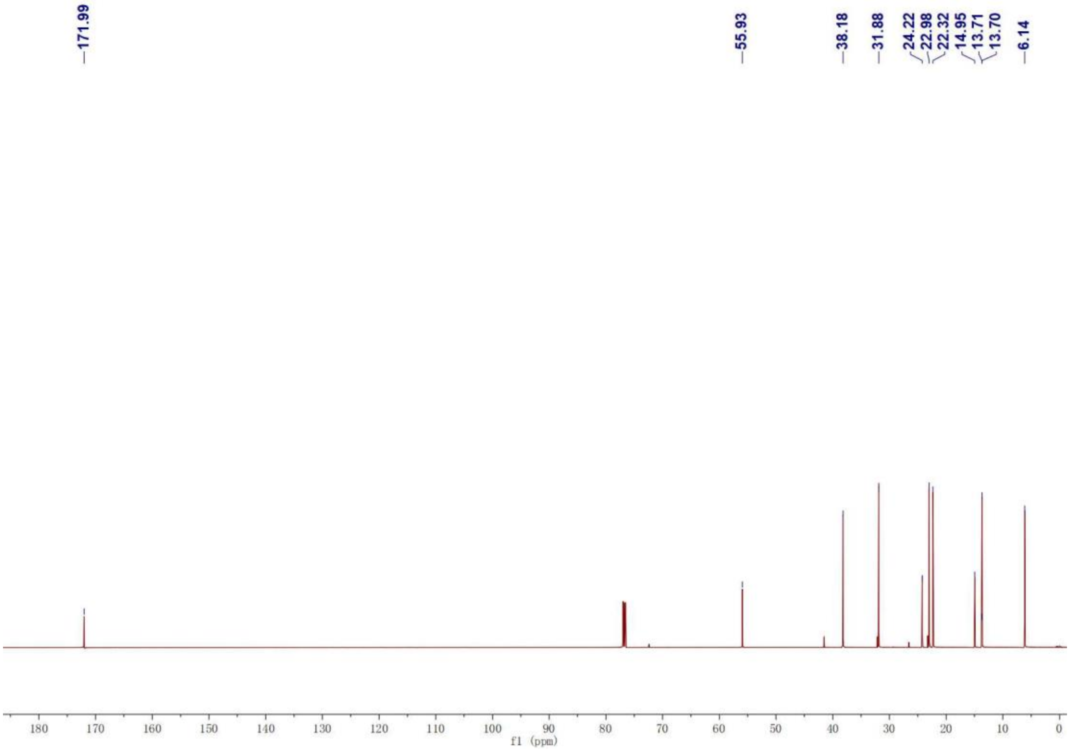

Supplementary Figure 88. ^{13}C NMR spectrum of 3w'
